# Supplementary material for: Repurposing Type I-A CRISPR-Cas3 for a robust diagnosis of human papillomavirus (HPV)
Source: Commun Biol. 2024 Jul 13;7:858. doi: 10.1038/s42003-024-06537-3 (PMC11246428; doi:10.1038/s42003-024-06537-3)
Supplement: Supplementary file 2 — Supplementary Information [file 42003_2024_6537_MOESM2_ESM.pdf]

# Supplementary Information

# Repurposing Type I-A CRISPR-Cas3 for a Robust Diagnosis of human papillomavirus (HPV)

Tao Hu[1], Quanquan Ji[2], Xinxin Ke[1], Hufeng Zhou[3], Senfeng Zhang[4], Shengsheng Ma[4], Chenlin Yu[5], Wenjun Ju[5], Meiling Lu[5], Yu Lin[6], Yangjing Ou[6], Yingsi Zhou[7], *, Yibei Xiao[5], *, Chunlong Xu[7,8,9,] *, Chunyi Hu[4,11,12, *, #]

[1] The Children's Hospital, Zhejiang University School of Medicine, National Clinical Research Center for Child Health, Zhejiang University, Hangzhou, Zhejiang, 310052, China.

[2] Cancer Science Institute of Singapore, National University of Singapore, Singapore, Singapore.

[3]Department of Biostatistics, Harvard T.H. Chan School of Public Health, Boston, MA, USA.

[4]Department of Biological Sciences, Faculty of Science, National University of Singapore, Singapore 117543, Singapore.

[5]Department of Biochemistry, School of Life Science and Technology, China Pharmaceutical University, Nanjing 211198, China.

[6]International Peace Maternity & Child Health Hospital, Shanghai Municipal Key Clinical Specialty, Institute of Embryo-Fetal Original Adult Disease, School of Medicine, Shanghai Jiao Tong University, Shanghai 200030, China.

[7]HuidaGene Therapeutics Inc., Shanghai, China.

[8] Lingang Laboratory, Shanghai, China.

[9]School of Life Sciences and Technology, ShanghaiTech University, Shanghai, China.

[10]Shanghai Center for Brain Science and Brain-Inspired Technology, Shanghai, China

[11]Department of Biochemistry, Yong Loo Lin School of Medicine, National University of Singapore, Singapore 117597, Singapore.

[12]Precision Medicine Translational Research Programme (TRP), Yong Loo Lin School of Medicine, National University of Singapore, Singapore 117597, Singapore.

# Lead contact.

* Correspondence: yingsizhou@huidagene.com, yibei.xiao@cpu.edu.cn, xucl@lglab.ac.cn, hu_dbs@nus.edu.sg

## Supplementary Figures

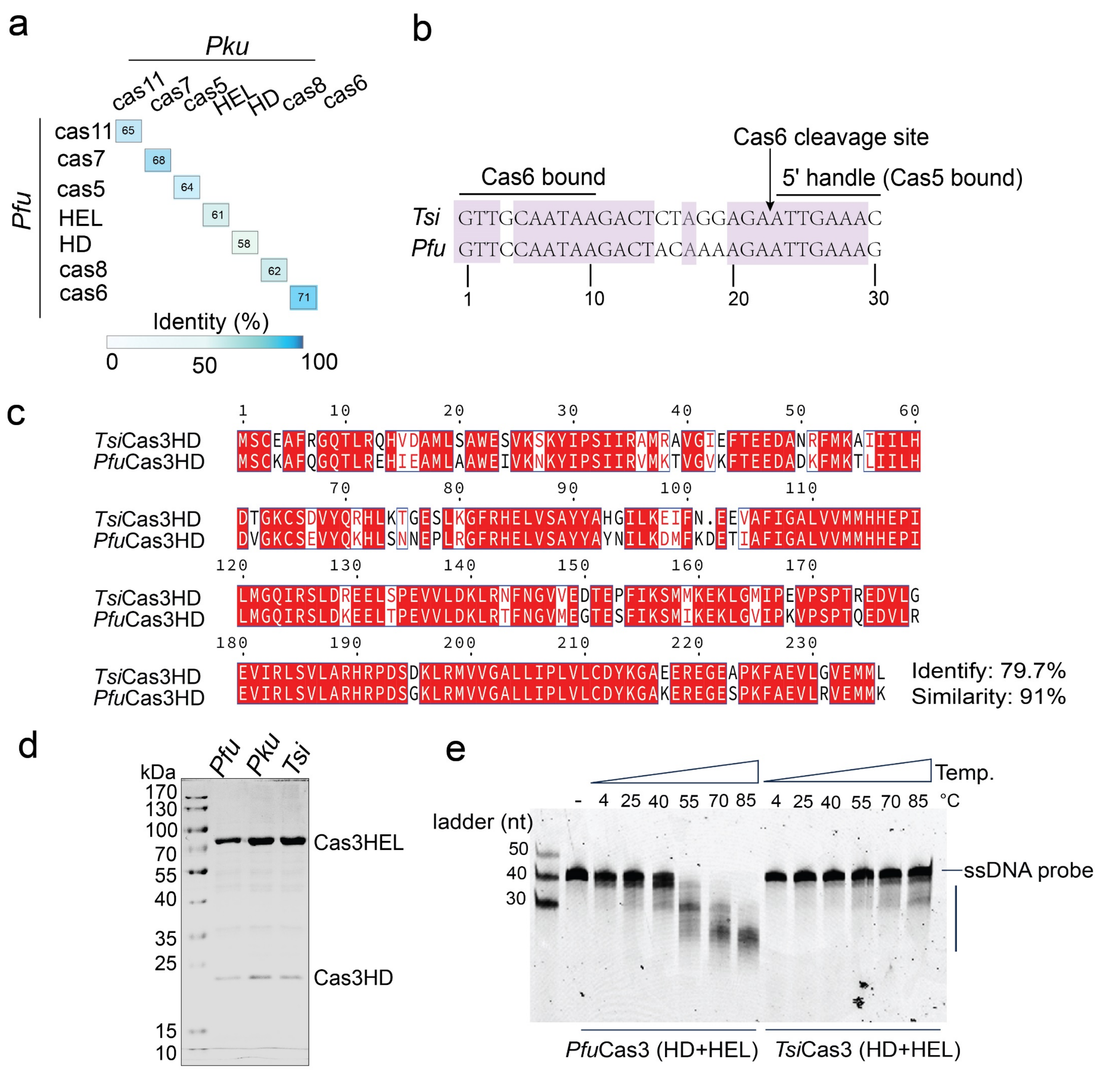


**Supplementary Figure S1. Identification and Analysis of the Type I-A CRISPR-Cas3 System.**

**a** Chart displaying the percentage identity of Type I-A CRISPR-Cas3 components between *Pfu* and *Pku*. **b** DNA sequence alignment for the Repeat region of crRNA between *Tsi* and *Pfu*. **c** Amino acid sequence alignment for Cas3HD between *Tsi* and *Pfu*. **d** Representative gel depicting the purification of the Cas3HEL/HD complex derived from *Pfu, Pku*, and *Tsi*. **e** Comparison of ssDNase activity between pfuCas3 and TsiCas3 across various Temperatures.

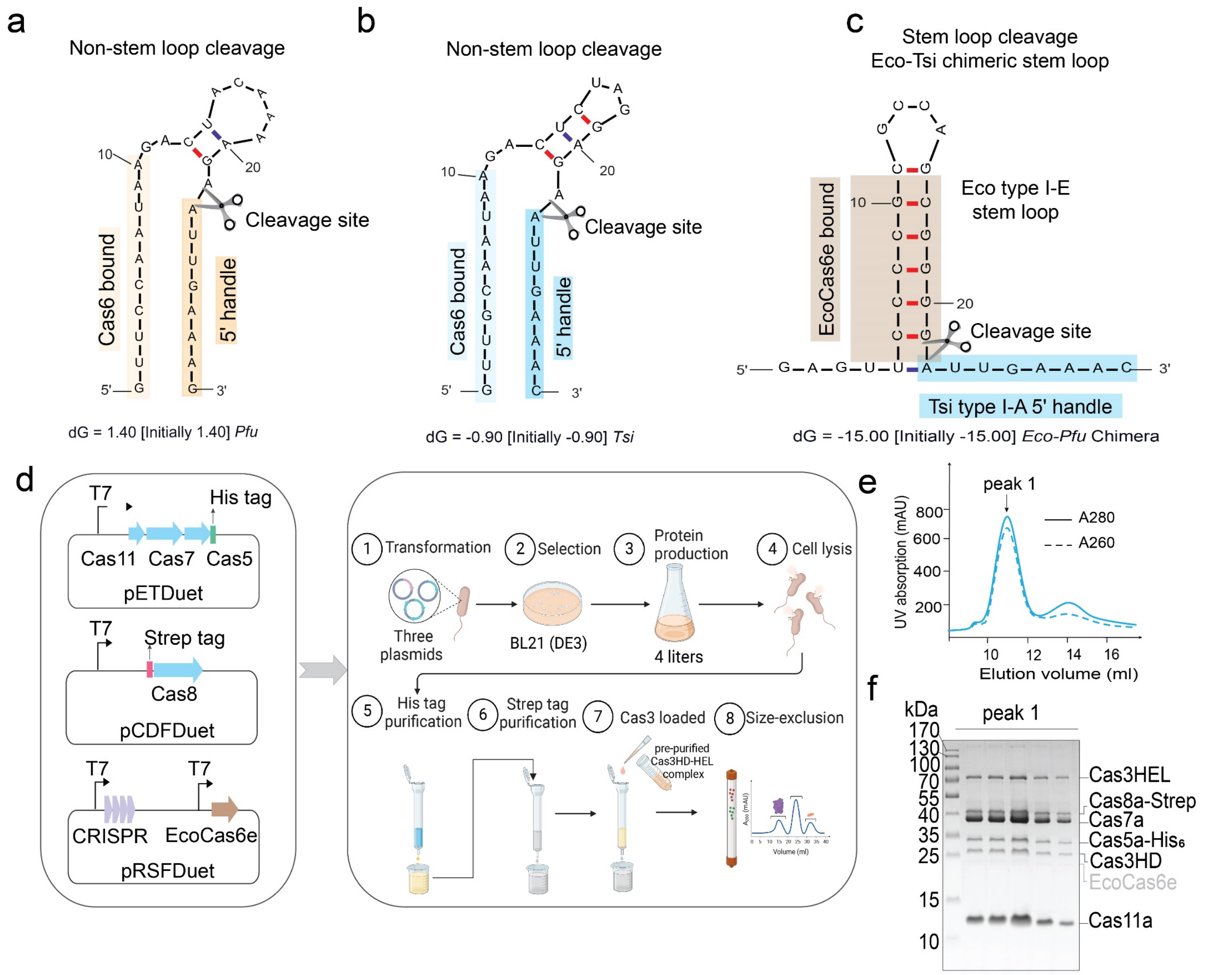


**Supplementary Figure S2. The strategy for the purification of Tsi Type I-A Cascade-Cas3 complex.** **a, b** Schematic representations of the crRNA Repeat structures from the *Pfu* (a) and *Tsi* (b) Type I-A systems, showing a non-stem loop-dependent cleavage by Cas6a. The cleavage sites are highlighted by scissor symbols. **c** Schematic representation of the chimeric structure of the *Eco* Type I-E system's crRNA Repeat, combined with the *Tsi* 5′ handle. This chimeric crRNA Repeat structure undergoes a stem loop-dependent cleavage by *Eco*Cas6e, with the cleavage site marked by a scissor symbol. **d** Left: Depiction of the plasmids used for purification. Right: The workflow for the protein purification. **e** The representative profile of gel-filtration of *Tsi*Cascade-Cas3 complex. **f** Representative SDS-PAGE gel analysis of the protein quality of the Cascade-Cas3 complex from panel **e**.

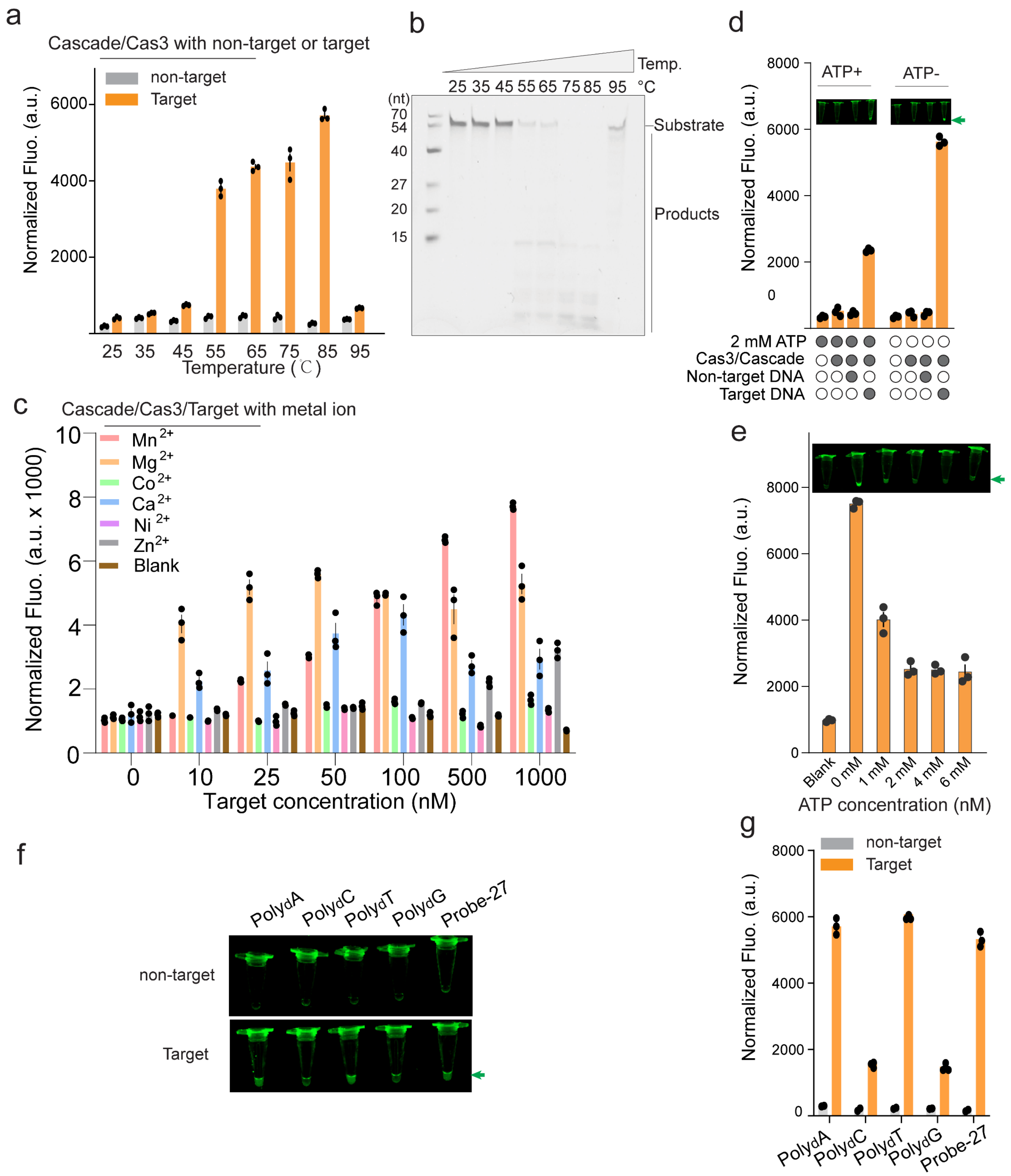


**Supplementary Figure S3: Analytical performance optimization of the TsiCascade-Cas3 System.** **a** Fluorescence analysis depicting the temperature-dependent nuclease activity of *Tsi*Cascade-Cas3 within the range of 25 °C to 95 °C, employing *Tsi*Cascade-Cas3 on F-Q ssDNA reporter. **b** Denaturing-PAGE assay illustrating the temperature titration of *Tsi*Cascade-Cas3 nuclease activity from 25 °C to 95 °C, employing *Tsi*Cascade-Cas3 on FAM labelled ssDNA substrate. **c** Assessment of divalent cation preferences for the *Tsi*Cascade-Cas3 mediated trans-cleavage system. **d** Analysis of *Tsi*Cascade-Cas3 trans-cleavage nuclease activity of F-Q ssDNA reporter in the presence of a 2 mM ATP reaction buffer

(+) compared to its activity in the absence of ATP buffer (-). **e** Titration of ATP concentrations and its impact on *Tsi*Cascade-Cas3 trans-cleavage nuclease activity on F-Q ssDNA reporter. **f, g** Fluorescence scanning (**f**) and quantitative data analysis (**g**) were performed to assess the collateral activity of the *Tsi*Cascade-Cas3 system using poly(d)A-FQ, poly(d)T-FQ, poly(d)G-FQ, poly(d)C-FQ, and probe-27 substrates.

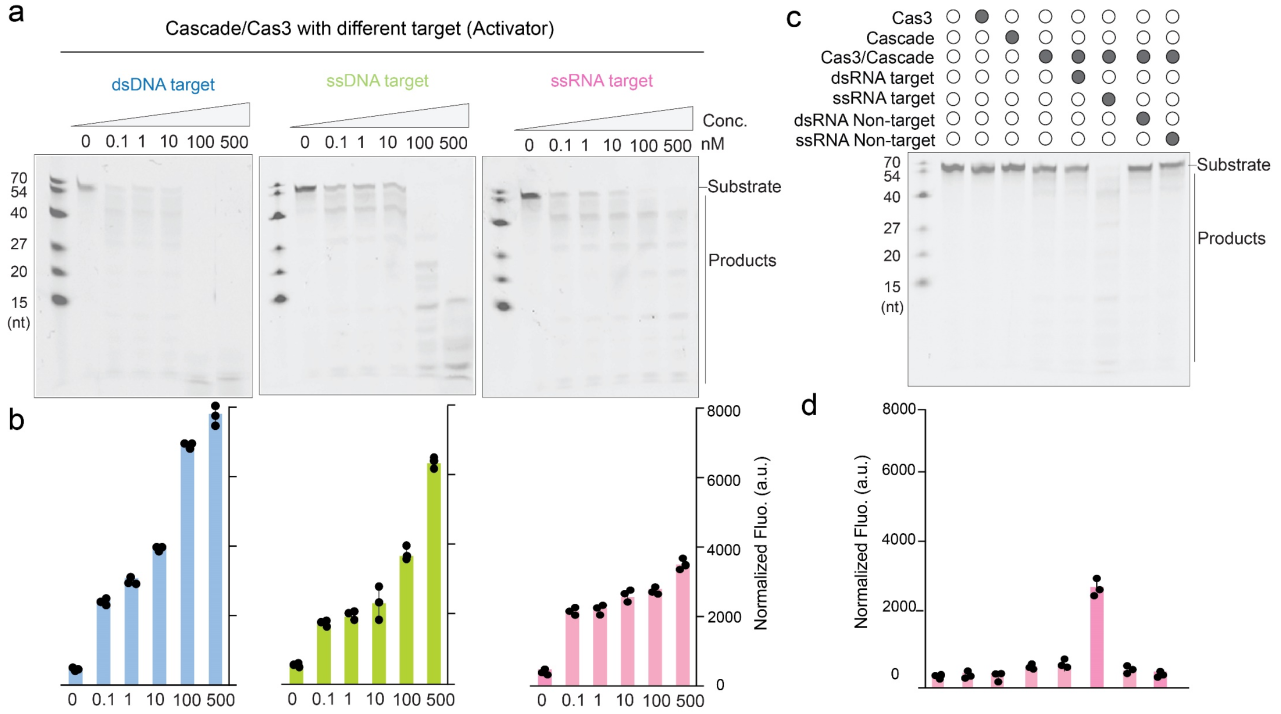


**Supplementary Figure S4: Comprehensive analysis of collateral ssDNA nuclease activity in the *Tsi* Type I-A CRISPR-Cas3 complex stimulated by DNA- and RNA-target Activators.**

**a** Denaturing PAGE analysis illustrating collateral trans-cleavage on the FAM-labeled ssDNA substrate with targeting of dsDNA, ssDNA, and ssRNA activators at varying concentrations (0, 0.1, 1, 10, 100, 500 nM). **b** Monitoring of fluorescence signals depicting collateral activity in the presence of different concentrations (0, 0.1, 1, 10, 100, 500 nM) of dsDNA, ssDNA, and ssRNA target activators as shown in panel **a**. **c** Denaturing PAGE results revealing the trans-cleavage activity of *Tsi*Cascade-Cas3 nuclease on the FAM-labeled ssDNA reporter when triggered by RNA-target activators. This analysis highlights that only ssRNA induces collateral trans-cleavage activity in *Tsi*Cascade-Cas3. **d** Fluorescence signal monitoring of the trans-cleavage activity of TsiCascade-Cas3 nuclease on the F-Q ssDNA reporter in response to RNA-target activators, corresponding to the observations in panel **c**.

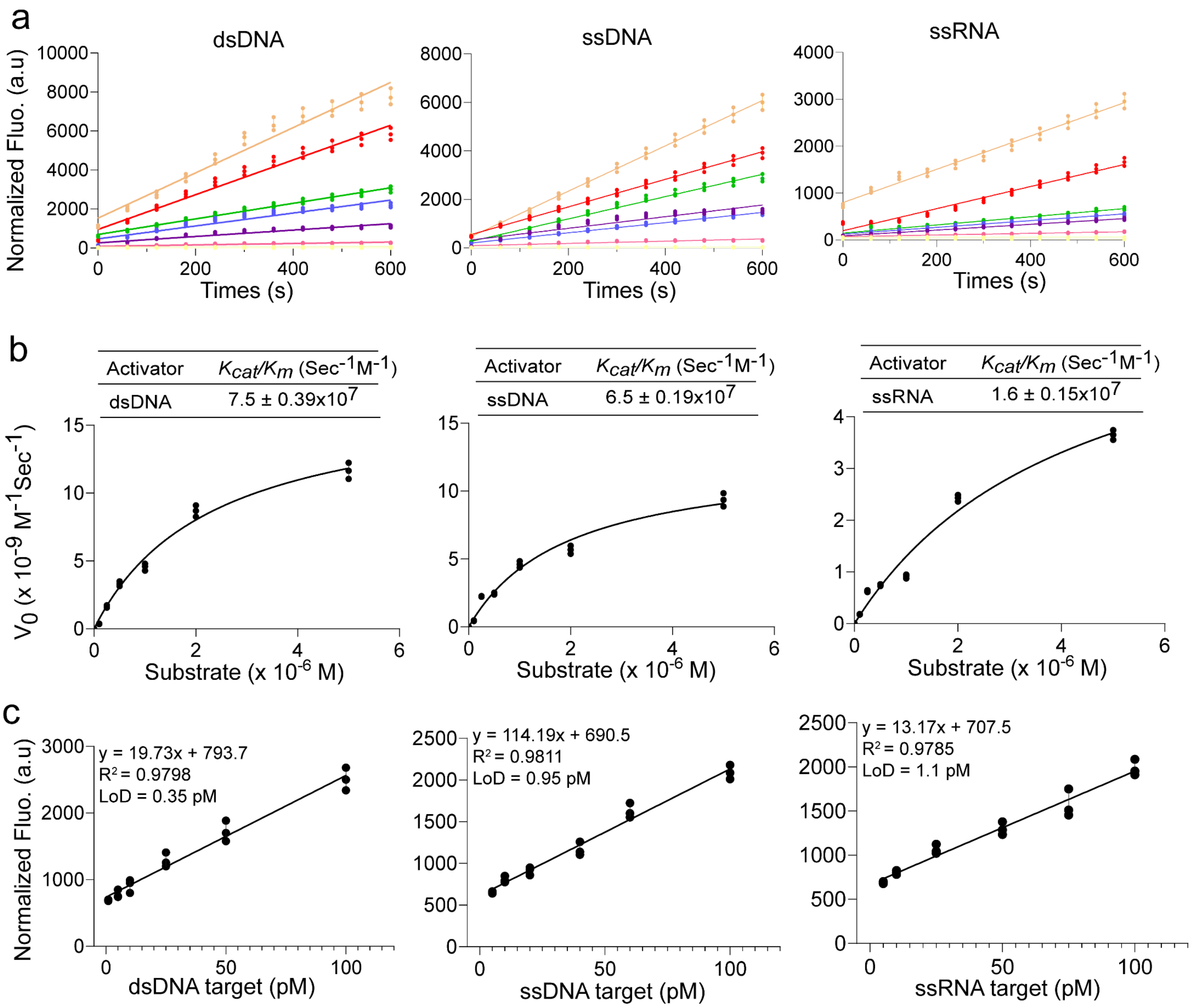


**Supplementary Figure S5. Michaelis-Menten analysis for quantification of trans-cleavage activity with DNA- and RNA-target activators.**

**a** Real-time fluorescence monitoring results displaying collateral trans-cleavage activity at varying concentrations of the F-Q reporter (1 nM - 5 μM) upon introduction of 10 nM dsDNA (left)-, ssDNA (middle)-, or ssRNA (right)-target activators. **b** Michaelis-Menten fits corresponding to the data in panel a, depicting dsDNA (left)-, ssDNA (middle)-, or ssRNA (right)- activators. **c** Calibration of the Type I-A CRISPR-Cas3 system across three targets. Error bars represent the standard deviation (SD) for n=3 measurements. The limit of detection (LOD), calculated using the 3σ/s method (where σ is the standard deviation of three blank samples and s is the slope of the calibration curve), was determined to be 0.35 pM for dsDNA (within a linear range of 1-100 pM), 0.95 pM for ssDNA (across a 5-100 pM range), and 1.1 pM for RNA (also within a 5-100 pM range), respectively.

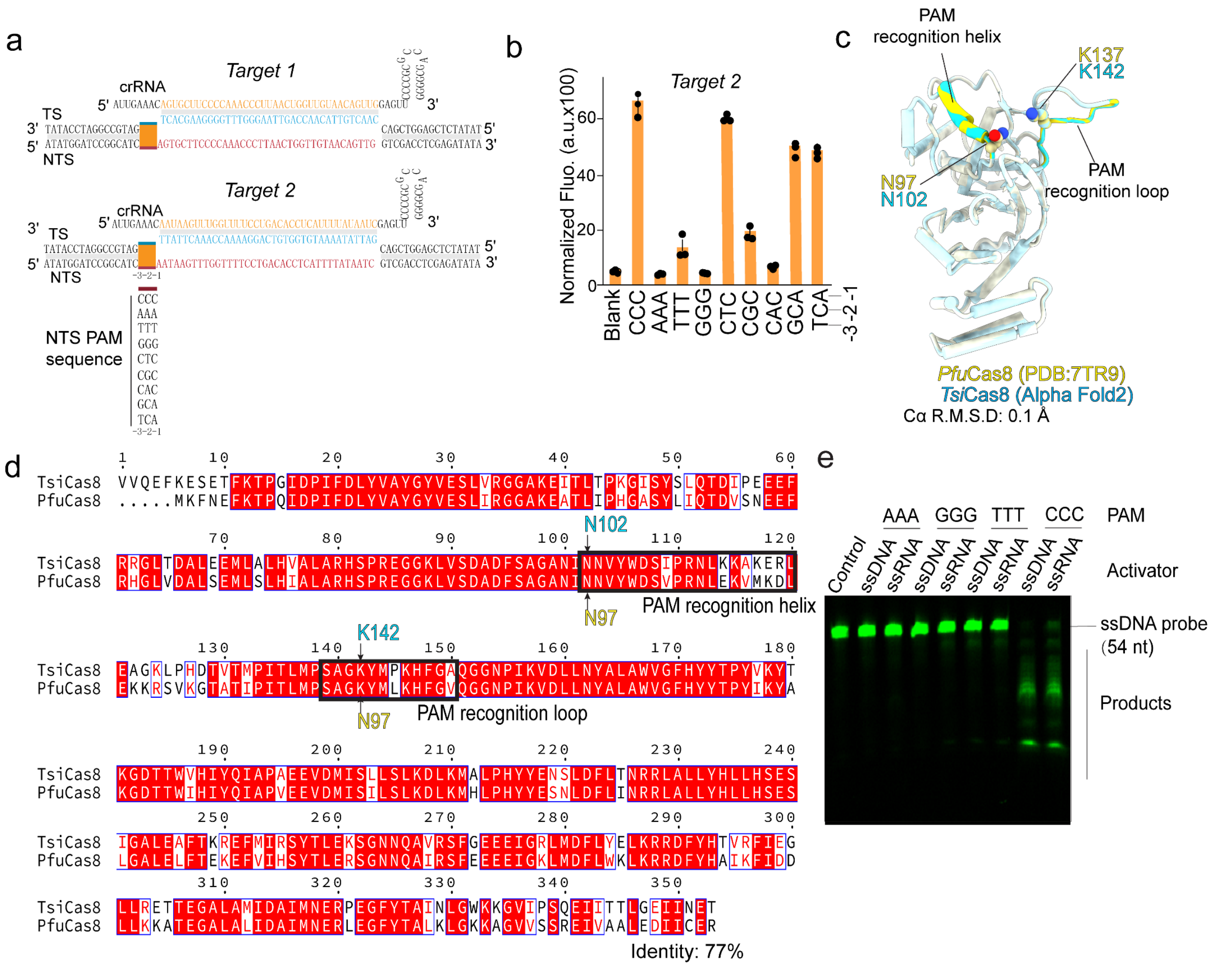


**Supplementary Figure S6. Comprehensive analysis of PAM recognition activity of *Tsi* Type I-A CRISPR-Cas3.**

**a** Schematic representation of R-loop formation and sequence display of target 1 and target 2, highlighting the PAM region in orange, and listing diverse PAM motifs. **b** Fluorescence monitoring assay illustrating the collateral activity of *Tsi*Cascade-Cas3 on the F-Q ssDNA reporter, with dsDNA target 2 featuring diverse PAM sequences. **c** Structural superposition between the AlphaFold2-predicted *Tsi*Cas8 and the determined *Pfu*Cas8 structure (PDB: 7TR9). Notable structural elements, including the theoretical PAM recognition helix, PAM recognition loop, and specific residues from both *Tsi*Cas8 and *Pfu*Cas8, are highlighted. **d** Amino acid sequence alignment between *Tsi*Cas8 and *Pfu*Cas8, highlighting shared PAM recognition residues for comparative analysis. **e** Denaturing-PAGE gel presenting the trans-cleavage activity of *Tsi*Cascade-Cas3 on the FAM labelled ssDNA probe, employing ssRNA targets with varying poly-deoxyribonucleotide PAM sequences.

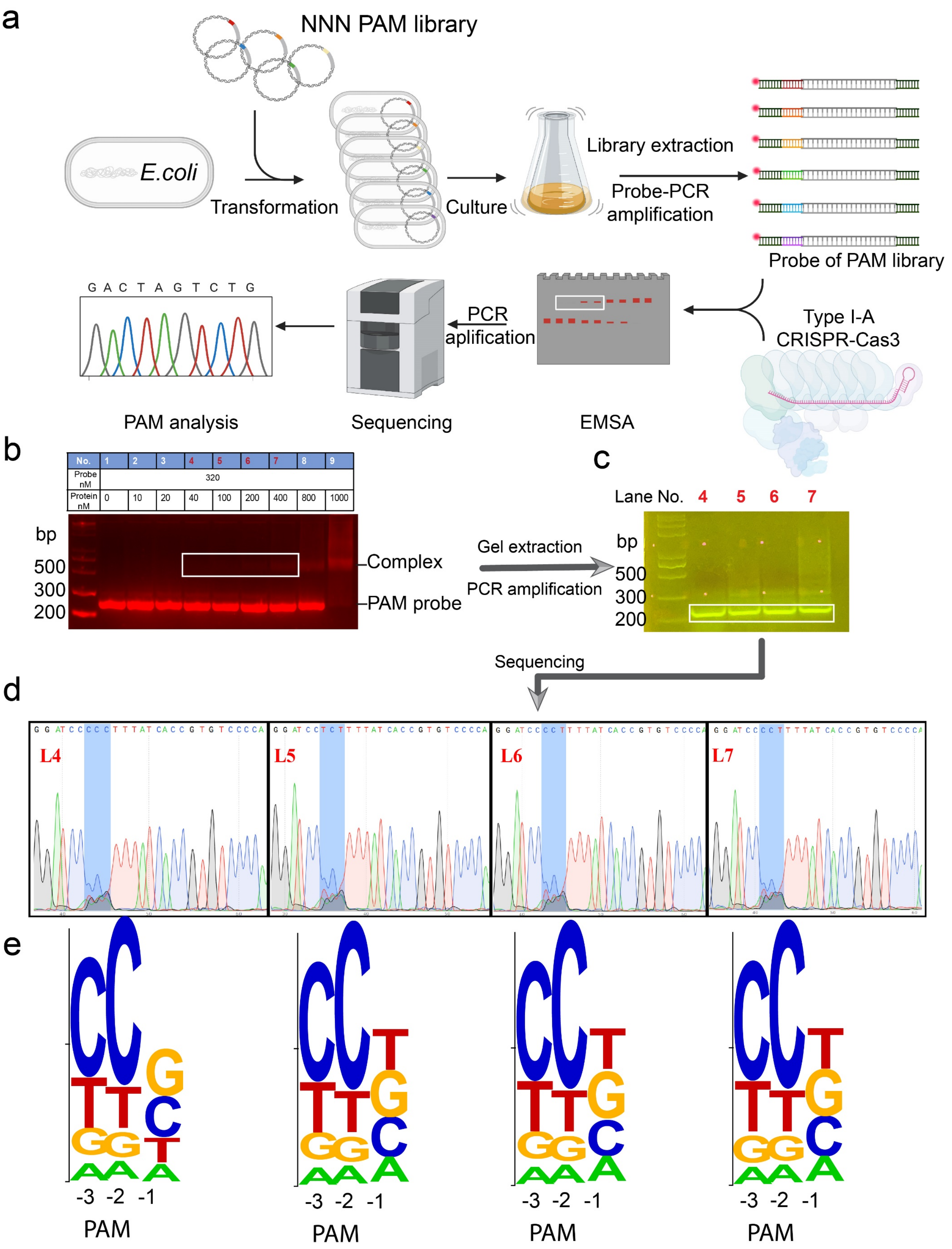


**Supplementary Figure S7. Biochemical analysis of PAM motif recognition in *Tsi* Type I-A CRISPR-Cas3.**

**a** Schematic representation of the method used for PAM motif determination through biochemical assays. A target plasmid library encompassing 64 PAM motifs was constructed. The Cy5-labeled probe of the PAM library was generated via PCR. After conducting an Electrophoretic mobility shift assay (EMSA)

assay with increasing concentrations of Cascade-Cas3, specific lanes were excised for further amplification and final sequencing. **b** EMSA showing the interaction between the PAM library probe and Cascade-Cas3 at varying concentrations. Highlighted white boxes indicate bands selected for sequencing. **c** Agarose gel displaying the excised probe bands after further PCR amplification. **d** DNA sequencing results corresponding to the selected lanes, with the PAM motif regions highlighted in blue boxes. **e** Computational logo generated from the sequencing results presented in panel **d**.

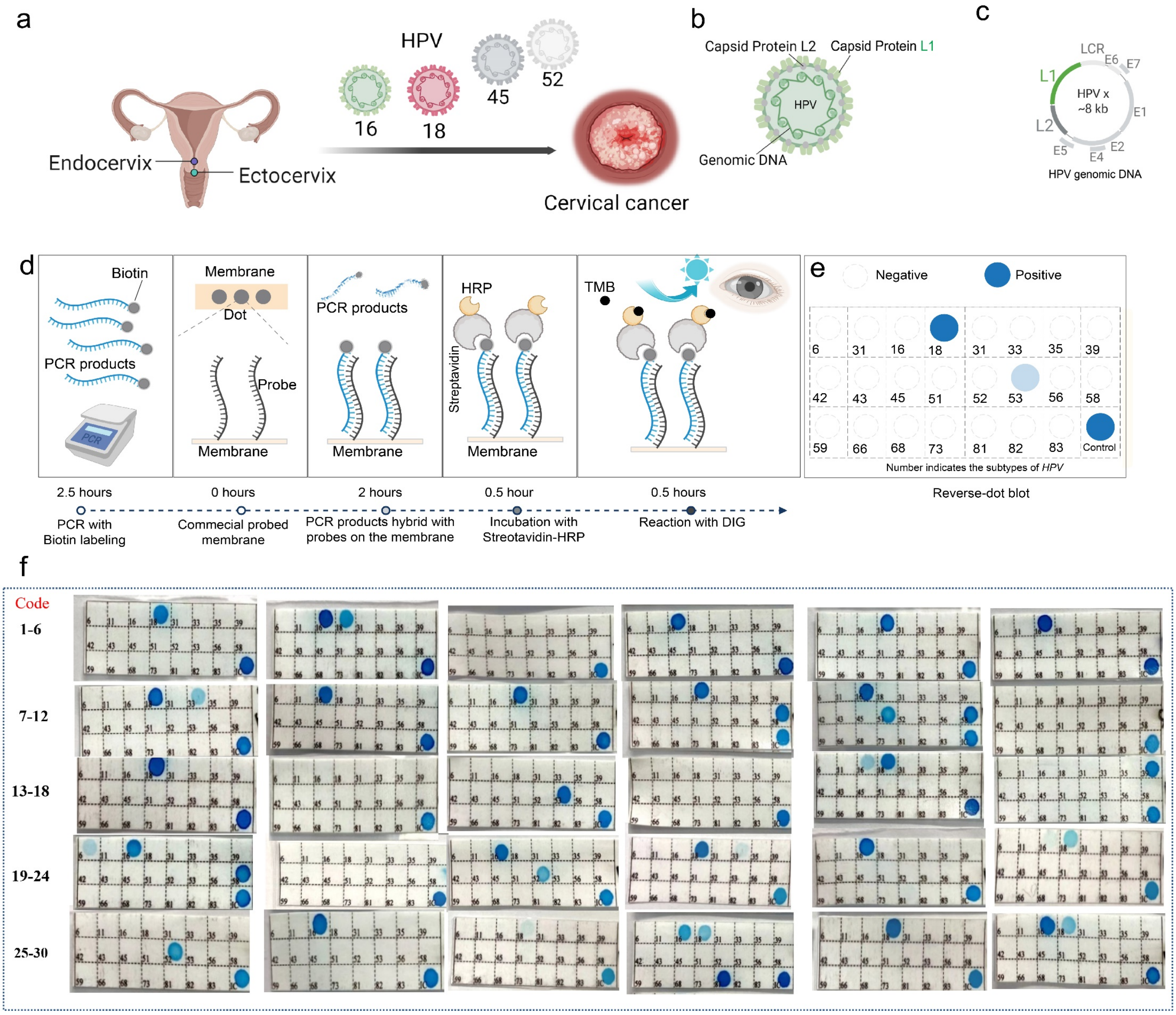


**Supplementary Figure S8. Selection of HPV clinical samples for assessment of the HAVE Platform.** **a** Illustration depicting the evolutionary progression of cervical cancer by HPV. The number showing different subtypes. **b** Visualization of the structural components of the HPV virus, including genomic DNA, capsid protein L1, and capsid protein L2. **c** Schematic representations of HPV genomic DNA fragments. **d** Workflow schemes outlining the PCR-Reverse Dot Blot array process. The five procedural steps, along with detailed procedures and time requirements, are delineated in the five boxes. **e** Visual representation of positive and negative results. In this diagram, 23 HPV subtypes were diagnosed, along with one positive control. Each box labeled the number corresponds to a detected HPV subtype, with positive results indicated by blue dots. **f** HPV virus identification results for 30 clinical samples, as determined by

PCR-Reverse Dot Blot analysis. The results pertaining to subtypes 16 and 18 were incorporated and presented in **Figure 6d**.

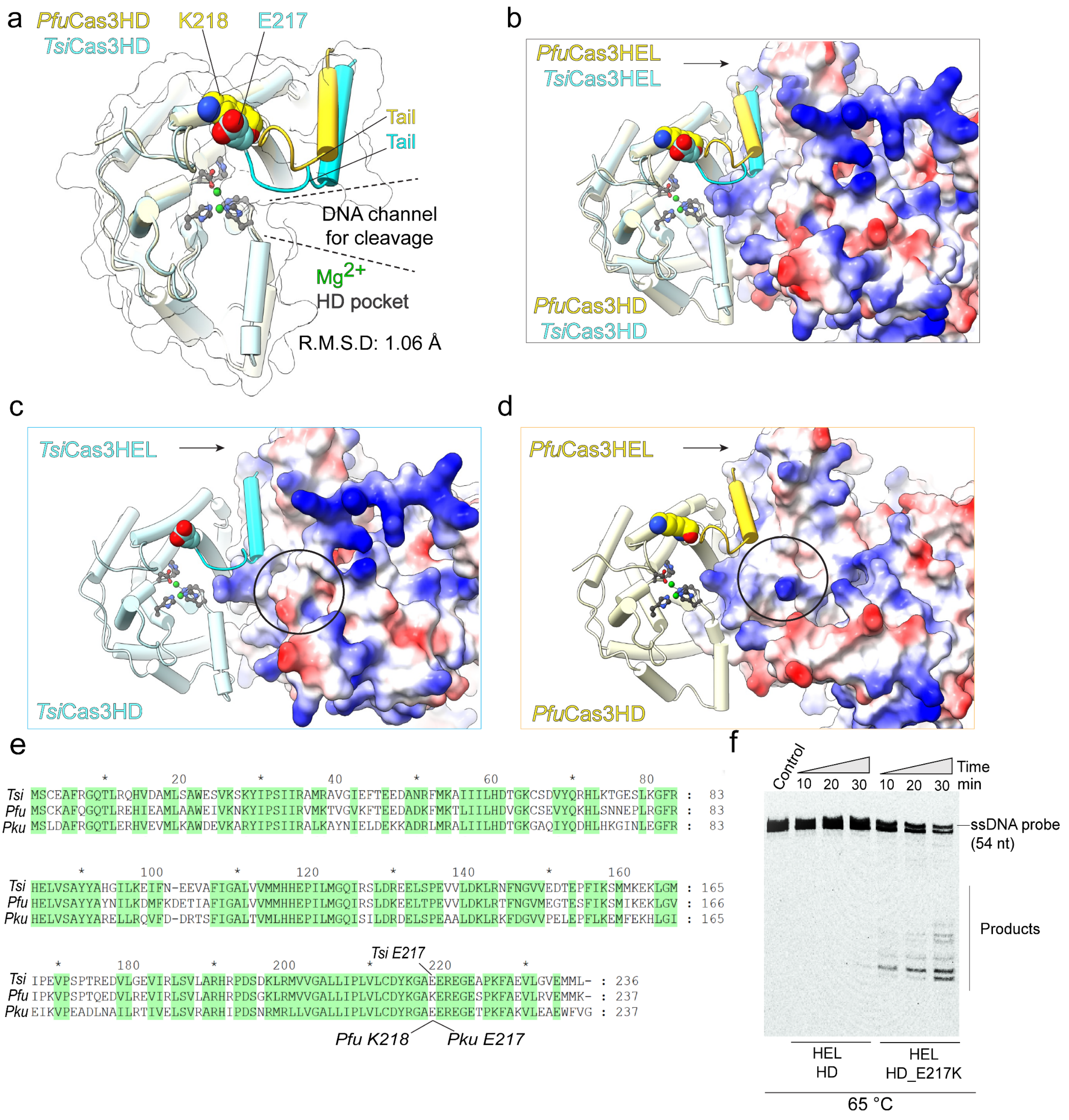


**Supplementary Figure S9. Hypothetical analysis of the auto-Inhibition mechanism in *Tsi*Cas3.**
**a** Structural superposition between *Pfu*Cas3HD and *Tsi*Cas3HD, with the HD nuclease pocket highlighted by gray-sticked-residues and green magnesium ions. The proposed "gate residues," responsible for modulating nuclease activity, are highlighted in sphere model. The C-terminal tails of *Tsi*Cas3HD and *Pfu*Cas3HD are colored cyan and yellow, respectively. **b** Structural superposition between *Pfu*Cas3HD-HEL complex and *Tsi*Cas3HD-HEL complex. The HD components are shown as a cartoon model, while HEL components are represented as electric potential surface. **c, d** Structural models of *Tsi*Cas3HD-HEL (**c**) and *Pfu*Cas3HD-HEL (**d**) from panel **b** The black circle highlights the opposite electric potential on

HEL between *Tsi* and *Pfu*. **e** Amino acid sequence alignment among Tsi, Pfu, and Pku Cas3HD Components. **f** ssDNA cleavage assay conducted on TsiCas3 wildtype and HD E317K mutant with HEL to validate the auto-inhibition hypothesis.

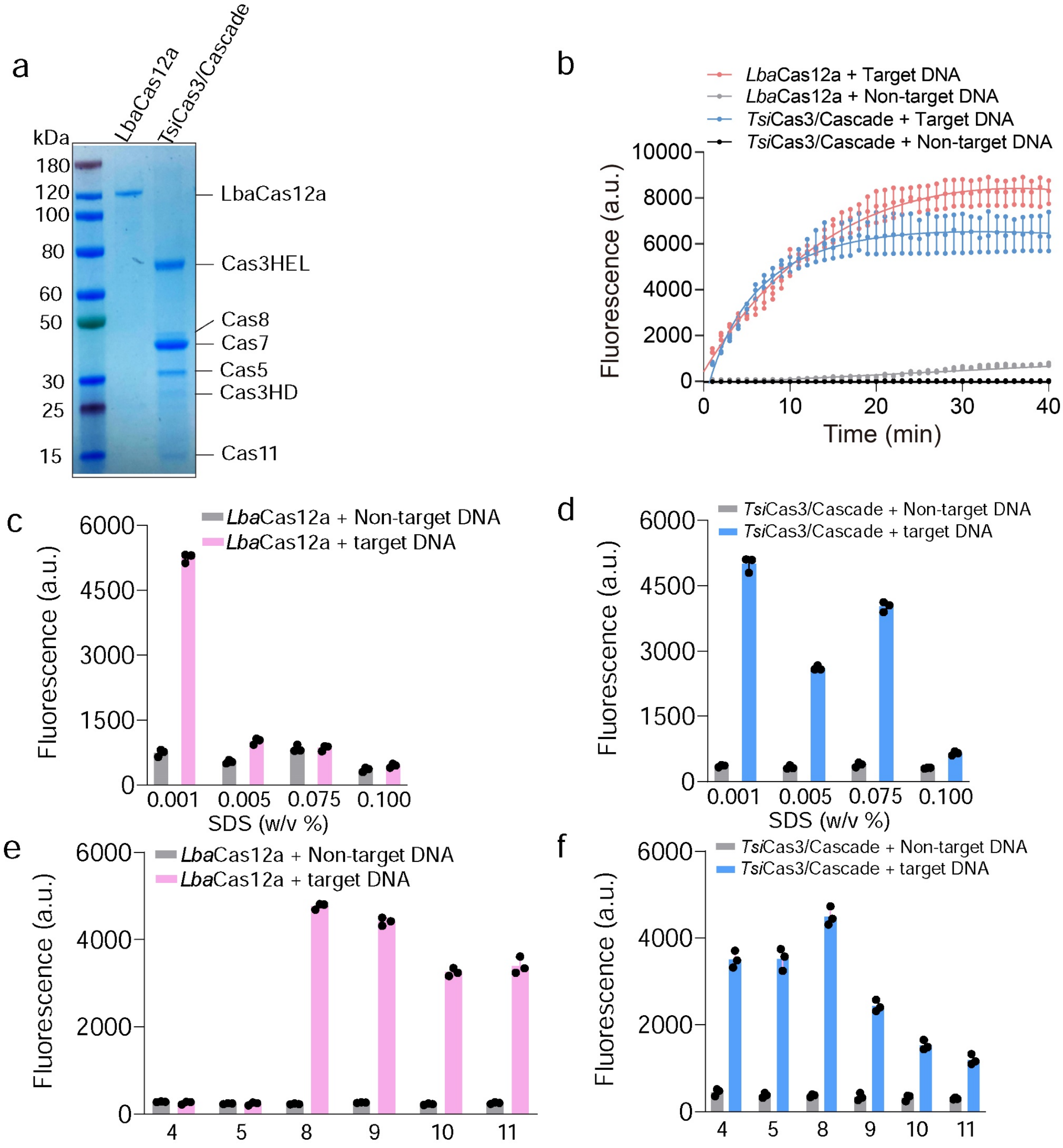


**Supplementary Figure S10. Comparative performance analysis of *Tsi*Cascade-Cas3 and *Lba*Cas12a Systems.**

**a** SDS-PAGE analysis presenting the purified *Lba*Cas12a protein and *Tsi*Cascade-Cas3 complex in a lane-by-lane format. **b** Real-time fluorescence monitoring of trans-cleavage activity, comparing *Tsi*Cascade-Cas3 and *Lba*Cas12a with identical 10 nM target DNA and non-target DNA activators. **c** Assessment of the Influence of SDS component in the reaction buffer on *Lba*Cas12a nuclease activity. **d** Impact of SDS component in the reaction buffer on the nuclease activity of *Tsi*Cascade-Cas3. **e**

Assessment of the Influence of pH values in the reaction buffer on *Lba*Cas12a nuclease activity. (f) Assessment of the Influence of pH values in the reaction buffer on *Tsi*Cascade-Cas3 nuclease activity.

## Supplementary Reference

(1) Broughton, J. P. *et al*. CRISPR-Cas12-based detection of SARS-CoV-2. *Nat Biotechnol* **38**, 870-874, doi:10.1038/s41587-020-0513-4 (2020).

(2) Nguyen, L. T. *et al*. Enhancement of trans-cleavage activity of Cas12a with engineered crRNA enables amplified nucleic acid detection. *Nat Commun* **11**, 4906, doi:10.1038/s41467-020-18615-1 (2020).

(3) Ramachandran, A. *et al*. "Electric field-driven microfluidics for rapid CRISPR-based diagnostics and its application to detection of SARS-CoV-2." *Proc Natl Acad Sci U S A* **117**, 29518-29525, doi:10.1073/pnas.2010254117 (2020).

(4) Ramachandran, A., Juan G. S. CRISPR Enzyme Kinetics for Molecular Diagnostics. *Anal Chem* **93**, 7456-7464, doi:10.1021/acs.analchem.1c00525 (2021).

(5) (1)Chen, J. S. *et al*. CRISPR-Cas12a target binding unleashes indiscriminate single-stranded DNase activity. *Science* **360**, 436-439. doi:10.1126/science.aar6245 (2018).

(6) Fozouni, P. *et al*. Amplification-free detection of SARS-CoV-2 with CRISPR-Cas13a and mobile phone microscopy. *Cell* **184**, 323-333.e9. doi:10.1016/j.cell.2020.12.001 (2021).

(7) Yue, H. *et al*. Droplet Cas12a Assay Enables DNA Quantification from Unamplified Samples at the Single-Molecule Level. *Nano Lett* **21**, 4643-4653. doi:10.1021/acs.nanolett.1c00715 (2021).

(8) Gootenberg, J. S. *et al*. Multiplexed and portable nucleic acid detection platform with Cas13, Cas12a, and Csm6. *Science* **360**, 439-444, doi:10.1126/science.aaq0179 (2018).
