# Supplementary material for: Repurposing Type I-A CRISPR-Cas3 for a robust diagnosis of human papillomavirus (HPV)
Source: Commun Biol. 2024 Jul 13;7:858. doi: 10.1038/s42003-024-06537-3 (PMC11246428; doi:10.1038/s42003-024-06537-3)
Supplement: Supplementary file 3 — Description of Additional Supplementary Files [file 42003_2024_6537_MOESM3_ESM.pdf]

Description of Additional Supplementary Files

File name: Supplementary Video 1

Description: Animated Overview of the article

File name: Supplementary Movie 1

Description: Detailed demonstration of device's practical detection capabilities using CRISPR-Cas3.

File name: Supplementary Data 1

Description: Contains all plasmid maps and sequences (DNA and protein sequences)

File name: Supplementary Data 2

Description: Contains oligonucleotide sequences and experimental data.(Resource data)
