# Supplementary material for: Repurposing Type I-A CRISPR-Cas3 for a robust diagnosis of human papillomavirus (HPV)
Source: Commun Biol. 2024 Jul 13;7:858. doi: 10.1038/s42003-024-06537-3 (PMC11246428; doi:10.1038/s42003-024-06537-3)
Supplement: Supplementary file 6 — Supplementary Data 1 [file 42003_2024_6537_MOESM6_ESM.pdf]

Oligonucleotides sequence used in this study

HPV16-RPA-F	GGGACAATGTGTCTGTAGATTATAAGCAGAC
HPV16-RPA-R	TCACCATCTTCCAAAACTGTGTTTTTAAGTTCT
HPV18-RPA-F	TTATAAGCAGACACAGTTATGTATTTTAGGC
HPV18-RPA-R	GCCCTGTGATAAAGGACGGGATTTACAAGCA
Trget DNA-NTS	ATATGGATCCGGCATCCCCAGTGCTTCCCCAAACCCTTAACTGGTTGT AACAGTTGGTCGACCTCGAGATATA
Target DNA-TS	TATATCTCGAGGTCGACCAACTGTTACAACCAGTTAAGGGTTTGGGG AAGCACTGGGGATGCCGGATCCATAT
RNA-NTS	GGCAUCCCCAGUGCUUCCCCAAACCCUUAACUGGUUGUAACAGUUG GUCG
RNA-TS	CGACCAACUGUUACAACCAGUUAAGGGUUUGGGGAAGCACUGGGG AUGCC
PAM1-DNA-NTS	ATATGGATCCGGCATCCCCAGTGCTTCCCCAAACCCTTAACTGGTTGT AACAGTTGGTCGACCTCGAGATATA
PAM1-DNA-TS	TATATCTCGAGGTCGACCAACTGTTACAACCAGTTAAGGGTTTGGGG AAGCACTGGGGATGCCGGATCCATAT
PAM2-DNA-NTS	ATATGGATCCGGCATCAAAAGTGCTTCCCCAAACCCTTAACTGGTTGT AACAGTTGGTCGACCTCGAGATATA
PAM2-DNA-TS	TATATCTCGAGGTCGACCAACTGTTACAACCAGTTAAGGGTTTGGGG AAGCACTTTTGATGCCGGATCCATAT
PAM3-DNA-NTS	ATATGGATCCGGCATCTTTAGTGCTTCCCCAAACCCTTAACTGGTTGT AACAGTTGGTCGACCTCGAGATATA
PAM3-DNA-TS	TATATCTCGAGGTCGACCAACTGTTACAACCAGTTAAGGGTTTGGGG AAGCACTAAAGATGCCGGATCCATAT
PAM4-DNA-NTS	ATATGGATCCGGCATCGGGAGTGCTTCCCCAAACCCTTAACTGGTTGT AACAGTTGGTCGACCTCGAGATATA
PAM4-DNA-TS	TATATCTCGAGGTCGACCAACTGTTACAACCAGTTAAGGGTTTGGGG AAGCACTCCCGATGCCGGATCCATAT
PAM5-DNA-NTS	ATATGGATCCGGCATCCTCAGTGCTTCCCCAAACCCTTAACTGGTTGT AACAGTTGGTCGACCTCGAGATATA
PAM5-DNA-TS	TATATCTCGAGGTCGACCAACTGTTACAACCAGTTAAGGGTTTGGGG AAGCACTGAGGATGCCGGATCCATAT
PAM6-DNA-NTS	ATATGGATCCGGCATCCGCAGTGCTTCCCCAAACCCTTAACTGGTTGT AACAGTTGGTCGACCTCGAGATATA
PAM6-DNA-TS	TATATCTCGAGGTCGACCAACTGTTACAACCAGTTAAGGGTTTGGGG AAGCACTGCGGATGCCGGATCCATAT
PAM7-DNA-NTS	ATATGGATCCGGCATCCACAGTGCTTCCCCAAACCCTTAACTGGTTGT AACAGTTGGTCGACCTCGAGATATA
PAM7-DNA-TS	TATATCTCGAGGTCGACCAACTGTTACAACCAGTTAAGGGTTTGGGG AAGCACTGTGGATGCCGGATCCATAT
PAM8-DNA-NTS	ATATGGATCCGGCATCGCAAGTGCTTCCCCAAACCCTTAACTGGTTGT AACAGTTGGTCGACCTCGAGATATA
PAM8-DNA-TS	TATATCTCGAGGTCGACCAACTGTTACAACCAGTTAAGGGTTTGGGG AAGCACTTGCGATGCCGGATCCATAT
PAM9-DNA-NTS	ATATGGATCCGGCATCTCAAGTGCTTCCCCAAACCCTTAACTGGTTGT AACAGTTGGTCGACCTCGAGATATA
PAM9-DNA-TS	TATATCTCGAGGTCGACCAACTGTTACAACCAGTTAAGGGTTTGGGG AAGCACTTGAGATGCCGGATCCATAT
**SsDNA-Reports**	
PolydA-FQ	FAM-AAAAAAAAAAAAAAA-BHQ1
PolydC-FQ	FAM-CCCCCCCCCCCCCCC-BHQ1
PolydG-FQ	FAM-AGGGGGGGGGGGGA-BHQ1
PolydT-FQ	FAM-TTTTTTTTTTTTTTTT-BHQ1
Probe-27	FAM-ATTTAGGATCCGTTATTGATCCCTTTT-BHQ1
ssDNA-Substrate-FAM	FAM-ACCTGGGGGAGTATTGCGGAGGAAGGTACCTGGGGGAGTATTGCGGA GGAAGGT
SsDNA-Substrate-FQ	FAM-ACCTGGGGGAGTATTGCGGAGGAAGGTACCTGGGGGAGTATTGCGGA GGAAGGT-BHQ1
Cas12a-FQ	FAM-TTTTAAATTTT-BHQ1

Mutation sequence (*Target 1*)	
M1-NTS	ATATGGATCCGGCATCCCCAGTGCTTCCCCAAACCCTTAACTGGTTGTAACAGTTGGTCGACCTCGAGATATA
M1-TS	TATATCTCGAGGTCGACCAACTGTTACAACCAGTTAAGGGTTTGGGGAAGCACTGGGGATGCCGGATCCATAT
M2-NTS	ATATGGATCCGGCATCCCCAGTGCTTCCCCAAACCCTTAACTGGTTGTAACAGTTGGTCGACCTCGAGATATA
M2-TS	TATATCTCGAGGTCGACCAACTGTTACAACCAGTTAAGGGTTTGGGGAAGCACTGGGGATGCCGGATCCATAT
M3-NTS	ATATGGATCCGGCATCCCCAGTGCTTCCCCAAACCCTTAACTGGTTGTAACAGTTGGTCGACCTCGAGATATA
M3-TS	TATATCTCGAGGTCGACCAACTGTTACAACCAGTTAAGGGTTTGGGGAAGCACTGGGGATGCCGGATCCATAT
M4-NTS	ATATGGATCCGGCATCCCCAGTGCTTCCCCAAACCCTTAACTGGTTGTAACAGTTGGTCGACCTCGAGATATA
M4-TS	TATATCTCGAGGTCGACCAACTGTTACAACCAGTTAAGGGTTTGGGGAAGCACTGGGGATGCCGGATCCATAT
M5-NTS	ATATGGATCCGGCATCCCCAGTGCTTCCCCAAACCCTTAACTGGTTGTAACAGTTGGTCGACCTCGAGATATA
M5-TS	TATATCTCGAGGTCGACCAACTGTTACAACCAGTTAAGGGTTTGGGGAAGCACTGGGGATGCCGGATCCATAT
M6-NTS	ATATGGATCCGGCATCCCCAGTGCTTCCCCAAACCCTTAACTGGTTGTAACAGTTGGTCGACCTCGAGATATA
M6-TS	TATATCTCGAGGTCGACCAACTGTTACAACCAGTTAAGGGTTTGGGGAAGCACTGGGGATGCCGGATCCATAT
M7-NTS	ATATGGATCCGGCATCCCCAGTGCTTCCCCAAACCCTTAACTGGTTGTAACAGTTGGTCGACCTCGAGATATA
M7-TS	TATATCTCGAGGTCGACCAACTGTTACAACCAGTTAAGGGTTTGGGGAAGCACTGGGGATGCCGGATCCATAT
M8-NTS	ATATGGATCCGGCATCCCCAGTGCTTCCCCAAACCCTTAACTGGTTGTAACAGTTGGTCGACCTCGAGATATA
M8-TS	TATATCTCGAGGTCGACCAACTGTTACAACCAGTTAAGGGTTTGGGGAAGCACTGGGGATGCCGGATCCATAT
M9-NTS	ATATGGATCCGGCATCCCCAGTGCTTCCCCAAACCCTTAACTGGTTGTAACAGTTGGTCGACCTCGAGATATA
M9-TS	TATATCTCGAGGTCGACCAACTGTTACAACCAGTTAAGGGTTTGGGGAAGCACTGGGGATGCCGGATCCATAT
M10-NTS	ATATGGATCCGGCATCCCCAGTGCTTCCCCAAACCCTTAACTGGTTGTAACAGTTGGTCGACCTCGAGATATA
M10-TS	TATATCTCGAGGTCGACCAACTGTTACAACCAGTTAAGGGTTTGGGGAAGCACTGGGGATGCCGGATCCATAT
M11-NTS	ATATGGATCCGGCATCCCCAGTGCTTCCCCAAACCCTTAACTGGTTGTAACAGTTGGTCGACCTCGAGATATA
M11-TS	TATATCTCGAGGTCGACCAACTGTTACAACCAGTTAAGGGTTTGGGGAAGCACTGGGGATGCCGGATCCATAT
M12-NTS	ATATGGATCCGGCATCCCCAGTGCTTCCCCAAACCCTTAACTGGTTGTAACAGTTGGTCGACCTCGAGATATA
M12-TS	TATATCTCGAGGTCGACCAACTGTTACAACCAGTTAAGGGTTTGGGGAAGCACTGGGGATGCCGGATCCATAT
M13-NTS	ATATGGATCCGGCATCCCCAGTGCTTCCCCAAACCCTTAACTGGTTGTAACAGTTGGTCGACCTCGAGATATA
M13-TS	TATATCTCGAGGTCGACCAACTGTTACAACCAGTTAAGGGTTTGGGGAAGCACTGGGGATGCCGGATCCATAT
M14-NTS	ATATGGATCCGGCATCCCCAGTGCTTCCCCAAACCCTTAACTGGTTGTAACAGTTGGTCGACCTCGAGATATA
M14-TS	TATATCTCGAGGTCGACCAACTGTTACAACCAGTTAAGGGTTTGGGGAAGCACTGGGGATGCCGGATCCATAT
M15-NTS	ATATGGATCCGGCATCCCCAGTGCTTCCCCAAACCCTTAACTGGTTGTAACAGTTGGTCGACCTCGAGATATA
M15-TS	TATATCTCGAGGTCGACCAACTGTTACAACCAGTTAAGGGTTTGGGGAAGCACTGGGGATGCCGGATCCATAT
M16-NTS	ATATGGATCCGGCATCCCCAGTGCTTCCCCAAACCCTTAACTGGTTGTAACAGTTGGTCGACCTCGAGATATA
M16-TS	TATATCTCGAGGTCGACCAACTGTTACAACCAGTTAAGGGTTTGGGGAAGCACTGGGGATGCCGGATCCATAT

M17-NTS	ATATGGATCCGGCATCCCCAGTGCTTCCCCAAACCCTTAACTGGTTGTAACAGTTGGTCGACCTCGAGATATA
M17-TS	TATATCTCGAGGTCGACCAACTGTTACAACCAGTTAAGGGTTTGGGGAAGCACTGGGGATGCCGGATCCATAT
M18-NTS	ATATGGATCCGGCATCCCCAGTGCTTCCCCAAACCCTTAACTGGTTGTAACAGTTGGTCGACCTCGAGATATA
M18-TS	TATATCTCGAGGTCGACCAACTGTTACAACCAGTTAAGGGTTTGGGGAAGCACTGGGGATGCCGGATCCATAT
M19-NTS	ATATGGATCCGGCATCCCCAGTGCTTCCCCAAACCCTTAACTGGTTGTAACAGTTGGTCGACCTCGAGATATA
M19-TS	TATATCTCGAGGTCGACCAACTGTTACAACCAGTTAAGGGTTTGGGGAAGCACTGGGGATGCCGGATCCATAT
M20-NTS	ATATGGATCCGGCATCCCCAGTGCTTCCCCAAACCCTTAACTGGTTGTAACAGTTGGTCGACCTCGAGATATA
M20-TS	TATATCTCGAGGTCGACCAACTGTTACAACCAGTTAAGGGTTTGGGGAAGCACTGGGGATGCCGGATCCATAT
M21-NTS	ATATGGATCCGGCATCCCCAGTGCTTCCCCAAACCCTTAACTGGTTGTAACAGTTGGTCGACCTCGAGATATA
M21-TS	TATATCTCGAGGTCGACCAACTGTTACAACCAGTTAAGGGTTTGGGGAAGCACTGGGGATGCCGGATCCATAT
M22-NTS	ATATGGATCCGGCATCCCCAGTGCTTCCCCAAACCCTTAACTGGTTGTAACAGTTGGTCGACCTCGAGATATA
M22-TS	TATATCTCGAGGTCGACCAACTGTTACAACCAGTTAAGGGTTTGGGGAAGCACTGGGGATGCCGGATCCATAT
M23-NTS	ATATGGATCCGGCATCCCCAGTGCTTCCCCAAACCCTTAACTGGTTGTAACAGTTGGTCGACCTCGAGATATA
M23-TS	TATATCTCGAGGTCGACCAACTGTTACAACCAGTTAAGGGTTTGGGGAAGCACTGGGGATGCCGGATCCATAT
M24-NTS	ATATGGATCCGGCATCCCCAGTGCTTCCCCAAACCCTTAACTGGTTGTAACAGTTGGTCGACCTCGAGATATA
M24-TS	TATATCTCGAGGTCGACCAACTGTTACAACCAGTTAAGGGTTTGGGGAAGCACTGGGGATGCCGGATCCATAT
M25-NTS	ATATGGATCCGGCATCCCCAGTGCTTCCCCAAACCCTTAACTGGTTGTAACAGTTGGTCGACCTCGAGATATA
M25-TS	TATATCTCGAGGTCGACCAACTGTTACAACCAGTTAAGGGTTTGGGGAAGCACTGGGGATGCCGGATCCATAT
M26-NTS	ATATGGATCCGGCATCCCCAGTGCTTCCCCAAACCCTTAACTGGTTGTAACAGTTGGTCGACCTCGAGATATA
M26-TS	TATATCTCGAGGTCGACCAACTGTTACAACCAGTTAAGGGTTTGGGGAAGCACTGGGGATGCCGGATCCATAT
M27-NTS	ATATGGATCCGGCATCCCCAGTGCTTCCCCAAACCCTTAACTGGTTGTAACAGTTGGTCGACCTCGAGATATA
M27-TS	TATATCTCGAGGTCGACCAACTGTTACAACCAGTTAAGGGTTTGGGGAAGCACTGGGGATGCCGGATCCATAT
M28-NTS	ATATGGATCCGGCATCCCCAGTGCTTCCCCAAACCCTTAACTGGTTGTAACAGTTGGTCGACCTCGAGATATA
M28-TS	TATATCTCGAGGTCGACCAACTGTTACAACCAGTTAAGGGTTTGGGGAAGCACTGGGGATGCCGGATCCATAT
M29-NTS	ATATGGATCCGGCATCCCCAGTGCTTCCCCAAACCCTTAACTGGTTGTAACAGTTGGTCGACCTCGAGATATA
M29-TS	TATATCTCGAGGTCGACCAACTGTTACAACCAGTTAAGGGTTTGGGGAAGCACTGGGGATGCCGGATCCATAT
M30-NTS	ATATGGATCCGGCATCCCCAGTGCTTCCCCAAACCCTTAACTGGTTGTAACAGTTGGTCGACCTCGAGATATA
M30-TS	TATATCTCGAGGTCGACCAACTGTTACAACCAGTTAAGGGTTTGGGGAAGCACTGGGGATGCCGGATCCATAT
M31-NTS	ATATGGATCCGGCATCCCCAGTGCTTCCCCAAACCCTTAACTGGTTGTAACAGTTGGTCGACCTCGAGATATA
M31-TS	TATATCTCGAGGTCGACCAACTGTTACAACCAGTTAAGGGTTTGGGGAAGCACTGGGGATGCCGGATCCATAT
M32-NTS	ATATGGATCCGGCATCCCCAGTGCTTCCCCAAACCCTTAACTGGTTGTAACAGTTGGTCGACCTCGAGATATA
M32-TS	TATATCTCGAGGTCGACCAACTGTTACAACCAGTTAAGGGTTTGGGGAAGCACTGGGGATGCCGGATCCATAT

M33-NTS	ATATGGATCCGGCATCCCCAGTGCTTCCCCAAACCCTTAACTGGTTGTAACAG TTGGTCGACCTCGAGATATA
M33-TS	TATATCTCGAGGTCGACCAACTGTTACAACCAGTTAAGGGTTTGGGGAAGCA CTGGGGATGCCGGATCCATAT
M34-NTS	ATATGGATCCGGCATCCCCAGTGCTTCCCCAAACCCTTAACTGGTTGTAACAG TTGGTCGACCTCGAGATATA
M34-TS	TATATCTCGAGGTCGACCAACTGTTACAACCAGTTAAGGGTTTGGGGAAGCA CTGGGGATGCCGGATCCATAT
M35-NTS	ATATGGATCCGGCATCCCCAGTGCTTCCCCAAACCCTTAACTGGTTGTAACAG TTGGTCGACCTCGAGATATA
M35-TS	TATATCTCGAGGTCGACCAACTGTTACAACCAGTTAAGGGTTTGGGGAAGCA CTGGGGATGCCGGATCCATAT
M36-NTS	ATATGGATCCGGCATCCCCAGTGCTTCCCCAAACCCTTAACTGGTTGTAACAG TTGGTCGACCTCGAGATATA
M36-TS	TATATCTCGAGGTCGACCAACTGTTACAACCAGTTAAGGGTTTGGGGAAGCA CTGGGGATGCCGGATCCATAT
M37-NTS	ATATGGATCCGGCATCCCCAGTGCTTCCCCAAACCCTTAACTGGTTGTAACAG TTGGTCGACCTCGAGATATA
M37-TS	TATATCTCGAGGTCGACCAACTGTTACAACCAGTTAAGGGTTTGGGGAAGCA CTGGGGATGCCGGATCCATAT
M1-2-NTS	ATATGGATCCGGCATCCCCAGTGCTTCCCCAAACCCTTAACTGGTTGTAACAG TTGGTCGACCTCGAGATATA
M1-2-TS	TATATCTCGAGGTCGACCAACTGTTACAACCAGTTAAGGGTTTGGGGAAGCA CTGGGGATGCCGGATCCATAT
M3-4-NTS	ATATGGATCCGGCATCCCCAGTGCTTCCCCAAACCCTTAACTGGTTGTAACAG TTGGTCGACCTCGAGATATA
M3-4-TS	TATATCTCGAGGTCGACCAACTGTTACAACCAGTTAAGGGTTTGGGGAAGCA CTGGGGATGCCGGATCCATAT
M5-6-NTS	ATATGGATCCGGCATCCCCAGTGCTTCCCCAAACCCTTAACTGGTTGTAACAG TTGGTCGACCTCGAGATATA
M5-6-TS	TATATCTCGAGGTCGACCAACTGTTACAACCAGTTAAGGGTTTGGGGAAGCA CTGGGGATGCCGGATCCATAT
M7-8-NTS	ATATGGATCCGGCATCCCCAGTGCTTCCCCAAACCCTTAACTGGTTGTAACAG TTGGTCGACCTCGAGATATA
M7-8-TS	TATATCTCGAGGTCGACCAACTGTTACAACCAGTTAAGGGTTTGGGGAAGCA CTGGGGATGCCGGATCCATAT
M9-10-NTS	ATATGGATCCGGCATCCCCAGTGCTTCCCCAAACCCTTAACTGGTTGTAACAG TTGGTCGACCTCGAGATATA
M9-10-TS	TATATCTCGAGGTCGACCAACTGTTACAACCAGTTAAGGGTTTGGGGAAGCA CTGGGGATGCCGGATCCATAT
M11-12-NTS	ATATGGATCCGGCATCCCCAGTGCTTCCCCAAACCCTTAACTGGTTGTAACAG TTGGTCGACCTCGAGATATA
M11-12-TS	TATATCTCGAGGTCGACCAACTGTTACAACCAGTTAAGGGTTTGGGGAAGCA CTGGGGATGCCGGATCCATAT
M13-14-NTS	ATATGGATCCGGCATCCCCAGTGCTTCCCCAAACCCTTAACTGGTTGTAACAG TTGGTCGACCTCGAGATATA
M13-14-TS	TATATCTCGAGGTCGACCAACTGTTACAACCAGTTAAGGGTTTGGGGAAGCA CTGGGGATGCCGGATCCATAT
M15-16-NTS	ATATGGATCCGGCATCCCCAGTGCTTCCCCAAACCCTTAACTGGTTGTAACAG TTGGTCGACCTCGAGATATA
M15-16-TS	TATATCTCGAGGTCGACCAACTGTTACAACCAGTTAAGGGTTTGGGGAAGCA CTGGGGATGCCGGATCCATAT
M17-18-NTS	ATATGGATCCGGCATCCCCAGTGCTTCCCCAAACCCTTAACTGGTTGTAACAG TTGGTCGACCTCGAGATATA
M17-18-TS	TATATCTCGAGGTCGACCAACTGTTACAACCAGTTAAGGGTTTGGGGAAGCA CTGGGGATGCCGGATCCATAT
M19-20-NTS	ATATGGATCCGGCATCCCCAGTGCTTCCCCAAACCCTTAACTGGTTGTAACAG TTGGTCGACCTCGAGATATA
M19-20-TS	TATATCTCGAGGTCGACCAACTGTTACAACCAGTTAAGGGTTTGGGGAAGCA CTGGGGATGCCGGATCCATAT
M21-22-NTS	ATATGGATCCGGCATCCCCAGTGCTTCCCCAAACCCTTAACTGGTTGTAACAG TTGGTCGACCTCGAGATATA
M21-22-TS	TATATCTCGAGGTCGACCAACTGTTACAACCAGTTAAGGGTTTGGGGAAGCA CTGGGGATGCCGGATCCATAT

M23-24-NTS	ATATGGATCCGGCATCCCCAGTGCTTCCCCAAACCCTTAACTGGTTGTAACAGTTGGTCGACCTCGAGATATA
M23-24-TS	TATATCTCGAGGTCGACCAACTGTTACAACCAGTTAAGGGTTTGGGGAAGCACTGGGGATGCCGGATCCATAT
M25-26-NTS	ATATGGATCCGGCATCCCCAGTGCTTCCCCAAACCCTTAACTGGTTGTAACAGTTGGTCGACCTCGAGATATA
M25-26-TS	TATATCTCGAGGTCGACCAACTGTTACAACCAGTTAAGGGTTTGGGGAAGCACTGGGGATGCCGGATCCATAT
M27-28-NTS	ATATGGATCCGGCATCCCCAGTGCTTCCCCAAACCCTTAACTGGTTGTAACAGTTGGTCGACCTCGAGATATA
M27-28-TS	TATATCTCGAGGTCGACCAACTGTTACAACCAGTTAAGGGTTTGGGGAAGCACTGGGGATGCCGGATCCATAT
M29-30-NTS	ATATGGATCCGGCATCCCCAGTGCTTCCCCAAACCCTTAACTGGTTGTAACAGTTGGTCGACCTCGAGATATA
M29-30-TS	TATATCTCGAGGTCGACCAACTGTTACAACCAGTTAAGGGTTTGGGGAAGCACTGGGGATGCCGGATCCATAT
M31-32-NTS	ATATGGATCCGGCATCCCCAGTGCTTCCCCAAACCCTTAACTGGTTGTAACAGTTGGTCGACCTCGAGATATA
M31-32-TS	TATATCTCGAGGTCGACCAACTGTTACAACCAGTTAAGGGTTTGGGGAAGCACTGGGGATGCCGGATCCATAT
M33-34-NTS	ATATGGATCCGGCATCCCCAGTGCTTCCCCAAACCCTTAACTGGTTGTAACAGTTGGTCGACCTCGAGATATA
M33-34-TS	TATATCTCGAGGTCGACCAACTGTTACAACCAGTTAAGGGTTTGGGGAAGCACTGGGGATGCCGGATCCATAT
M35-37-NTS	ATATGGATCCGGCATCCCCAGTGCTTCCCCAAACCCTTAACTGGTTGTAACAGTTGGTCGACCTCGAGATATA
M35-37-TS	TATATCTCGAGGTCGACCAACTGTTACAACCAGTTAAGGGTTTGGGGAAGCACTGGGGATGCCGGATCCATAT

**Mutation sequence (*Target 2*)**	
M1-NTS	ATATGGATCCGGCATCCCCTTGCTATTGGGGAACACTGGGCTAAAGGCACTGCTTGGTCGACCTCGAGATATA
M1-TS	TATATCTCGAGGTCGACCAAGCAGTGCCTTTAGCCCAGTGTTCCCCAATAGCAAGGGGATGCCGGATCCATAT
M2-NTS	ATATGGATCCGGCATCCCCCGGCTATTGGGGAACACTGGGCTAAAGGCACTGCTTGGTCGACCTCGAGATATA
M2-TS	TATATCTCGAGGTCGACCAAGCAGTGCCTTTAGCCCAGTGTTCCCCAATAGCCGGGGGATGCCGGATCCATAT
M3-NTS	ATATGGATCCGGCATCCCCCTCCTATTGGGGAACACTGGGCTAAAGGCACTGCTTGGTCGACCTCGAGATATA
M3-TS	TATATCTCGAGGTCGACCAAGCAGTGCCTTTAGCCCAGTGTTCCCCAATAGGAGGGGGATGCCGGATCCATAT
M4-NTS	ATATGGATCCGGCATCCCCCTGATATTGGGGAACACTGGGCTAAAGGCACTGCTTGGTCGACCTCGAGATATA
M4-TS	TATATCTCGAGGTCGACCAAGCAGTGCCTTTAGCCCAGTGTTCCCCAATATCAGGGGGATGCCGGATCCATAT
M5-NTS	ATATGGATCCGGCATCCCCCTGCAATTGGGGAACACTGGGCTAAAGGCACTGCTTGGTCGACCTCGAGATATA
M5-TS	TATATCTCGAGGTCGACCAAGCAGTGCCTTTAGCCCAGTGTTCCCCAATTGCAGGGGGATGCCGGATCCATAT
M6-NTS	ATATGGATCCGGCATCCCCCTGCTATTGGGGAACACTGGGCTAAAGGCACTGCTTGGTCGACCTCGAGATATA
M6-TS	TATATCTCGAGGTCGACCAAGCAGTGCCTTTAGCCCAGTGTTCCCCAATAGCAGGGGGATGCCGGATCCATAT
M7-NTS	ATATGGATCCGGCATCCCCCTGCTATTGGGGAACACTGGGCTAAAGGCACTGCTTGGTCGACCTCGAGATATA
M7-TS	TATATCTCGAGGTCGACCAAGCAGTGCCTTTAGCCCAGTGTTCCCCAATAGCAGGGGGATGCCGGATCCATAT
M8-NTS	ATATGGATCCGGCATCCCCCTGCTATTGGGGAACACTGGGCTAAAGGCACTGCTTGGTCGACCTCGAGATATA
M8-TS	TATATCTCGAGGTCGACCAAGCAGTGCCTTTAGCCCAGTGTTCCCCAATAGCAGGGGGATGCCGGATCCATAT

M9-NTS	ATATGGATCCGGCATCCCCCTGCTATTGGGGAACACTGGGCTAAAGGCACTG CTTGGTCGACCTCGAGATATA
M9-TS	TATATCTCGAGGTCGACCAAGCAGTGCCTTTAGCCCAGTGTTCCCCAATAGCA GGGGGATGCCGGATCCATAT
M10-NTS	ATATGGATCCGGCATCCCCCTGCTATTGCGGAACACTGGGCTAAAGGCACTGC TTGGTCGACCTCGAGATATA
M10-TS	TATATCTCGAGGTCGACCAAGCAGTGCCTTTAGCCCAGTGTTCCGCAATAGCA GGGGGATGCCGGATCCATAT
M11-NTS	ATATGGATCCGGCATCCCCCTGCTATTGGGGAACACTGGGCTAAAGGCACTG CTTGGTCGACCTCGAGATATA
M11-TS	TATATCTCGAGGTCGACCAAGCAGTGCCTTTAGCCCAGTGTTCCCCAATAGCA GGGGGATGCCGGATCCATAT
M12-NTS	ATATGGATCCGGCATCCCCCTGCTATTGGGAAACACTGGGCTAAAGGCACTG CTTGGTCGACCTCGAGATATA
M12-TS	TATATCTCGAGGTCGACCAAGCAGTGCCTTTAGCCCAGTGTTTCCCAATAGCA GGGGGATGCCGGATCCATAT
M13-NTS	ATATGGATCCGGCATCCCCCTGCTATTGGGGTACACTGGGCTAAAGGCACTGC TTGGTCGACCTCGAGATATA
M13-TS	TATATCTCGAGGTCGACCAAGCAGTGCCTTTAGCCCAGTGTACCCCAATAGCA GGGGGATGCCGGATCCATAT
M14-NTS	ATATGGATCCGGCATCCCCCTGCTATTGGGGAGCACTGGGCTAAAGGCACTG CTTGGTCGACCTCGAGATATA
M14-TS	TATATCTCGAGGTCGACCAAGCAGTGCCTTTAGCCCAGTGCTCCCCAATAGCA GGGGGATGCCGGATCCATAT
M15-NTS	ATATGGATCCGGCATCCCCCTGCTATTGGGGAACACTGGGCTAAAGGCACTG CTTGGTCGACCTCGAGATATA
M15-TS	TATATCTCGAGGTCGACCAAGCAGTGCCTTTAGCCCAGTGTTCCCCAATAGCA GGGGGATGCCGGATCCATAT
M16-NTS	ATATGGATCCGGCATCCCCCTGCTATTGGGGAACTCTGGGCTAAAGGCACTGC TTGGTCGACCTCGAGATATA
M16-TS	TATATCTCGAGGTCGACCAAGCAGTGCCTTTAGCCCAGAGTTCCCCAATAGCA GGGGGATGCCGGATCCATAT
M17-NTS	ATATGGATCCGGCATCCCCCTGCTATTGGGGAACACTGGGCTAAAGGCACTG CTTGGTCGACCTCGAGATATA
M17-TS	TATATCTCGAGGTCGACCAAGCAGTGCCTTTAGCCCAGTGTTCCCCAATAGCA GGGGGATGCCGGATCCATAT
M18-NTS	ATATGGATCCGGCATCCCCCTGCTATTGGGGAACACCGGGCTAAAGGCACTG CTTGGTCGACCTCGAGATATA
M18-TS	TATATCTCGAGGTCGACCAAGCAGTGCCTTTAGCCCGGTGTTCCCCAATAGCA GGGGGATGCCGGATCCATAT
M19-NTS	ATATGGATCCGGCATCCCCCTGCTATTGGGGAACACTGGGCTAAAGGCACTG CTTGGTCGACCTCGAGATATA
M19-TS	TATATCTCGAGGTCGACCAAGCAGTGCCTTTAGCCCAGTGTTCCCCAATAGCA GGGGGATGCCGGATCCATAT
M20-NTS	ATATGGATCCGGCATCCCCCTGCTATTGGGGAACACTGCGCTAAAGGCACTGC TTGGTCGACCTCGAGATATA
M20-TS	TATATCTCGAGGTCGACCAAGCAGTGCCTTTAGCGCAGTGTTCCCCAATAGCA GGGGGATGCCGGATCCATAT
M21-NTS	ATATGGATCCGGCATCCCCCTGCTATTGGGGAACACTGGCCTAAAGGCACTGC TTGGTCGACCTCGAGATATA
M21-TS	TATATCTCGAGGTCGACCAAGCAGTGCCTTTAGGCCAGTGTTCCCCAATAGCA GGGGGATGCCGGATCCATAT
M22-NTS	ATATGGATCCGGCATCCCCCTGCTATTGGGGAACACTGGGGTAAAGGCACTG CTTGGTCGACCTCGAGATATA
M22-TS	TATATCTCGAGGTCGACCAAGCAGTGCCTTTACCCCAGTGTTCCCCAATAGCA GGGGGATGCCGGATCCATAT
M23-NTS	ATATGGATCCGGCATCCCCCTGCTATTGGGGAACACTGGGCTAAAGGCACTG CTTGGTCGACCTCGAGATATA
M23-TS	TATATCTCGAGGTCGACCAAGCAGTGCCTTTAGCCCAGTGTTCCCCAATAGCA GGGGGATGCCGGATCCATAT
M24-NTS	ATATGGATCCGGCATCCCCCTGCTATTGGGGAACACTGGGCTAAAGGCACTG CTTGGTCGACCTCGAGATATA
M24-TS	TATATCTCGAGGTCGACCAAGCAGTGCCTTTAGCCCAGTGTTCCCCAATAGCA GGGGGATGCCGGATCCATAT

M25-NTS	ATATGGATCCGGCATCCCCCTGCTATTGGGGAACACTGGGCTAAAGGCACTGCTTGGTCGACCTCGAGATATA
M25-TS	TATATCTCGAGGTCGACCAAGCAGTGCCTTTAGCCCAGTGTTCCCCAATAGCAGGGGGATGCCGGATCCATAT
M26-NTS	ATATGGATCCGGCATCCCCCTGCTATTGGGGAACACTGGGCTAACGGCACTGCTTGGTCGACCTCGAGATATA
M26-TS	TATATCTCGAGGTCGACCAAGCAGTGCCGTTAGCCCAGTGTTCCCCAATAGCAGGGGGATGCCGGATCCATAT
M27-NTS	ATATGGATCCGGCATCCCCCTGCTATTGGGGAACACTGGGCTAAAAGCACTGCTTGGTCGACCTCGAGATATA
M27-TS	TATATCTCGAGGTCGACCAAGCAGTGCTTTTAGCCCAGTGTTCCCCAATAGCAGGGGGATGCCGGATCCATAT
M28-NTS	ATATGGATCCGGCATCCCCCTGCTATTGGGGAACACTGGGCTAAAGCCACTGCTTGGTCGACCTCGAGATATA
M28-TS	TATATCTCGAGGTCGACCAAGCAGTGGCTTTAGCCCAGTGTTCCCCAATAGCAGGGGGATGCCGGATCCATAT
M29-NTS	ATATGGATCCGGCATCCCCCTGCTATTGGGGAACACTGGGCTAAAGGCACTGCTTGGTCGACCTCGAGATATA
M29-TS	TATATCTCGAGGTCGACCAAGCAGTGCCTTTAGCCCAGTGTTCCCCAATAGCAGGGGGATGCCGGATCCATAT
M30-NTS	ATATGGATCCGGCATCCCCCTGCTATTGGGGAACACTGGGCTAAAGGCACTGCTTGGTCGACCTCGAGATATA
M30-TS	TATATCTCGAGGTCGACCAAGCAGTGCCTTTAGCCCAGTGTTCCCCAATAGCAGGGGGATGCCGGATCCATAT
M31-NTS	ATATGGATCCGGCATCCCCCTGCTATTGGGGAACACTGGGCTAAAGGCACTGCTTGGTCGACCTCGAGATATA
M31-TS	TATATCTCGAGGTCGACCAAGCAGTGCCTTTAGCCCAGTGTTCCCCAATAGCAGGGGGATGCCGGATCCATAT
M32-NTS	ATATGGATCCGGCATCCCCCTGCTATTGGGGAACACTGGGCTAAAGGCACAGCTTGGTCGACCTCGAGATATA
M32-TS	TATATCTCGAGGTCGACCAAGCTGTGCCTTTAGCCCAGTGTTCCCCAATAGCAGGGGGATGCCGGATCCATAT
M33-NTS	ATATGGATCCGGCATCCCCCTGCTATTGGGGAACACTGGGCTAAAGGCACTCCTTGGTCGACCTCGAGATATA
M33-TS	TATATCTCGAGGTCGACCAAGGAGTGCCTTTAGCCCAGTGTTCCCCAATAGCAGGGGGATGCCGGATCCATAT
M34-NTS	ATATGGATCCGGCATCCCCCTGCTATTGGGGAACACTGGGCTAAAGGCACTGCTTGGTCGACCTCGAGATATA
M34-TS	TATATCTCGAGGTCGACCAAGCAGTGCCTTTAGCCCAGTGTTCCCCAATAGCAGGGGGATGCCGGATCCATAT
M35-NTS	ATATGGATCCGGCATCCCCCTGCTATTGGGGAACACTGGGCTAAAGGCACTGCATGGTCGACCTCGAGATATA
M35-TS	TATATCTCGAGGTCGACCATGCAGTGCCTTTAGCCCAGTGTTCCCCAATAGCAGGGGGATGCCGGATCCATAT
M36-NTS	ATATGGATCCGGCATCCCCCTGCTATTGGGGAACACTGGGCTAAAGGCACTGCTTGGTCGACCTCGAGATATA
M36-TS	TATATCTCGAGGTCGACCAAGCAGTGCCTTTAGCCCAGTGTTCCCCAATAGCAGGGGGATGCCGGATCCATAT
M37-NTS	ATATGGATCCGGCATCCCCCTGCTATTGGGGAACACTGGGCTAAAGGCACTGCTTCGTCGACCTCGAGATATA
M37-TS	TATATCTCGAGGTCGACGAAGCAGTGCCTTTAGCCCAGTGTTCCCCAATAGCAGGGGGATGCCGGATCCATAT
M1-2-NTS	ATATGGATCCGGCATCCCCAAGCTATTGGGGAACACTGGGCTAAAGGCACTGCTTGGTCGACCTCGAGATATA
M1-2-TS	TATATCTCGAGGTCGACCAAGCAGTGCCTTTAGCCCAGTGTTCCCCAATAGCTTGGGGATGCCGGATCCATAT
M3-4-NTS	ATATGGATCCGGCATCCCCCTTTTATTGGGGAACACTGGGCTAAAGGCACTGCTTGGTCGACCTCGAGATATA
M3-4-TS	TATATCTCGAGGTCGACCAAGCAGTGCCTTTAGCCCAGTGTTCCCCAATAAAAGGGGGATGCCGGATCCATAT
M5-6-NTS	ATATGGATCCGGCATCCCCCTGCCGTTGGGGAACACTGGGCTAAAGGCACTGCTTGGTCGACCTCGAGATATA
M5-6-TS	TATATCTCGAGGTCGACCAAGCAGTGCCTTTAGCCCAGTGTTCCCCAACGGCAGGGGGATGCCGGATCCATAT

M7-8-NTS	ATATGGATCCGGCATCCCCCTGCTACCGGGGAACACTGGGCTAAAGGCACTGCTTGGTCGACCTCGAGATATA
M7-8-TS	TATATCTCGAGGTCGACCAAGCAGTGCCTTTAGCCCAGTGTTCCCCGGTAGCAGGGGGATGCCGGATCCATAT
M9-10-NTS	ATATGGATCCGGCATCCCCCTGCTATTAAGGAACACTGGGCTAAAGGCACTGCTTGGTCGACCTCGAGATATA
M9-10-TS	TATATCTCGAGGTCGACCAAGCAGTGCCTTTAGCCCAGTGTTCCTTAATAGCAGGGGGATGCCGGATCCATAT
M11-12-NTS	ATATGGATCCGGCATCCCCCTGCTATTGGATAACACTGGGCTAAAGGCACTGCTTGGTCGACCTCGAGATATA
M11-12-TS	TATATCTCGAGGTCGACCAAGCAGTGCCTTTAGCCCAGTGTTATCCAATAGCAGGGGGATGCCGGATCCATAT
M13-14-NTS	ATATGGATCCGGCATCCCCCTGCTATTGGGGCGCACTGGGCTAAAGGCACTGCTTGGTCGACCTCGAGATATA
M13-14-TS	TATATCTCGAGGTCGACCAAGCAGTGCCTTTAGCCCAGTGCGCCCCAATAGCAGGGGGATGCCGGATCCATAT
M15-16-NTS	ATATGGATCCGGCATCCCCCTGCTATTGGGGAAGGCTGGGCTAAAGGCACTGCTTGGTCGACCTCGAGATATA
M15-16-TS	TATATCTCGAGGTCGACCAAGCAGTGCCTTTAGCCCAGCCTTCCCCAATAGCAGGGGGATGCCGGATCCATAT
M17-18-NTS	ATATGGATCCGGCATCCCCCTGCTATTGGGGAACAAAGGGCTAAAGGCACTGCTTGGTCGACCTCGAGATATA
M17-18-TS	TATATCTCGAGGTCGACCAAGCAGTGCCTTTAGCCCTTTGTTCCCCAATAGCAGGGGGATGCCGGATCCATAT
M19-20-NTS	ATATGGATCCGGCATCCCCCTGCTATTGGGGAACACTATGCTAAAGGCACTGCTTGGTCGACCTCGAGATATA
M19-20-TS	TATATCTCGAGGTCGACCAAGCAGTGCCTTTAGCATAGTGTTCCCCAATAGCAGGGGGATGCCGGATCCATAT
M21-22-NTS	ATATGGATCCGGCATCCCCCTGCTATTGGGGAACACTGGTTTAAAGGCACTGCTTGGTCGACCTCGAGATATA
M21-22-TS	TATATCTCGAGGTCGACCAAGCAGTGCCTTTAAACCAGTGTTCCCCAATAGCAGGGGGATGCCGGATCCATAT
M23-24-NTS	ATATGGATCCGGCATCCCCCTGCTATTGGGGAACACTGGGCGGAAGGCACTGCTTGGTCGACCTCGAGATATA
M23-24-TS	TATATCTCGAGGTCGACCAAGCAGTGCCTTCCGCCCAGTGTTCCCCAATAGCAGGGGGATGCCGGATCCATAT
M25-26-NTS	ATATGGATCCGGCATCCCCCTGCTATTGGGGAACACTGGGCTACGGGCACTGCTTGGTCGACCTCGAGATATA
M25-26-TS	TATATCTCGAGGTCGACCAAGCAGTGCCCGTAGCCCAGTGTTCCCCAATAGCAGGGGGATGCCGGATCCATAT
M27-28-NTS	ATATGGATCCGGCATCCCCCTGCTATTGGGGAACACTGGGCTAAAACCACTGCTTGGTCGACCTCGAGATATA
M27-28-TS	TATATCTCGAGGTCGACCAAGCAGTGGTTTTAGCCCAGTGTTCCCCAATAGCAGGGGGATGCCGGATCCATAT
M29-30-NTS	ATATGGATCCGGCATCCCCCTGCTATTGGGGAACACTGGGCTAAAGGTTCTGCTTGGTCGACCTCGAGATATA
M29-30-TS	TATATCTCGAGGTCGACCAAGCAGAACCTTTAGCCCAGTGTTCCCCAATAGCAGGGGGATGCCGGATCCATAT
M31-32-NTS	ATATGGATCCGGCATCCCCCTGCTATTGGGGAACACTGGGCTAAAGGCATAGCTTGGTCGACCTCGAGATATA
M31-32-TS	TATATCTCGAGGTCGACCAAGCTATGCCTTTAGCCCAGTGTTCCCCAATAGCAGGGGGATGCCGGATCCATAT
M33-34-NTS	ATATGGATCCGGCATCCCCCTGCTATTGGGGAACACTGGGCTAAAGGCACTAATTGGTCGACCTCGAGATATA
M33-34-TS	TATATCTCGAGGTCGACCAATTAGTGCCTTTAGCCCAGTGTTCCCCAATAGCAGGGGGATGCCGGATCCATAT
M35-37-NTS	ATATGGATCCGGCATCCCCCTGCTATTGGGGAACACTGGGCTAAAGGCACTGCCCAGTCGACCTCGAGATATA
M35-37-TS	TATATCTCGAGGTCGACTGGGCAGTGCCTTTAGCCCAGTGTTCCCCAATAGCAGGGGGATGCCGGATCCATAT

Comparative Summary of Kinetic Parameters for Published Cas Enzymes and Our System.

Cas Enzyme	Target	k_{cat} (s^{-1})	k_M (M)	k_{cat}/k_M (M^{-1} S^{-1})	Refs
LbCas12a	dsDNA	0.38	3.70×10^{-7}	1.0×10^{6}	(1)
	ssDNA	0.58	3.10×10^{-7}	1.9×10^{6}	
	dsDNA	0.32	2.60×10^{-7}	1.2×10^{6}	(2)
	ssDNA	0.27	1.80×10^{-7}	1.4×10^{6}	
	dsDNA	0.078	8.20×10^{-8}	9.5×10^{5}	(3)
	ssDNA	0.098	1.20×10^{-7}	8.5×10^{5}	
	dsDNA	0.56	4.90×10^{-7}	1.1×10^{6}	(4)
	ssDNA	0.30	2.60×10^{-7}	1.2×10^{6}	
	dsDNA	0.028	8.70×10^{-7}	3.3×10^{4}	(5)
	ssDNA	0.022	4.50×10^{-7}	4.9×10^{4}	
LubCas13a	RNA	23	5.80×10^{-6}	4.0×10^{6}	(6)
	RNA	4850	5.8×10^{-7}	8.4×10^{9}	(7)
LwaCas13a	RNA	2.0	1.8×10^{-6}	1.1×10^{6}	(8)
TsiCas3	dsDNA	18.75	2.50×10^{-7}	$7.5 \pm 0.39 \times 10^{7}$	This work
	ssDNA	13.43	2.06×10^{-7}	$6.5 \pm 0.19 \times 10^{7}$	
	RNA	7.08	4.35×10^{-7}	$1.6 \pm 0.15 \times 10^{7}$	

Please reach out to the Reference in the Supplementary Information document.

Nucleotide sequences used in this study

>CRISPR-associated protein Cas7/Csa2

atgatgtacgtccgcataagcggtaggataaggctgaacgcccactccctcaacgcccaaggtggcggaggaaccaactacattgagat
aaccaagaccaaggtaacagtaaagaccggaaacggctgggccgtcgttgaagtgcccgcgataaccggcaacatgctgaagcactgg
catttcacaggcttcgtggactacttcaagaccactccatacgggatgaacctcacagagcgagccttgagatacaacggaacgaggttt
ggacgggcgagactactgcaaccaaagccaatagtgcaactgtccaattaaacgatgaggccacaataattggggaattggctgatgcag
atgttcacggcttcctggccccgaagacaggcgtaagaagagtggctctagtcaaggttccatttatactcccagcggaggactttatccgc
gaagtggagggggagcgtctcataactgcaataaagcacaacagggttgacgtcaacgagagcggtgccataagcagcggtaacgaca
ccgcgcagatgctgttcagcagggaatacgccactggtctctacggcttctccatcgtccttgacctgggtcttgtgggcgttcctcagagct
caccggtaaaattcgagaagaaccagctaaagcccaacatcgtcatagaccctaacgagagaaaggcaagaatcgaaagcgccctca
aggccctagtaccaatgctcagtggctacctcggagcgaacctcgcccgttcgttcccggtattcaaggttgaggaactaattgccattgct
agcgaagagccgattcccgctctcgtccacggattctacaatgactacatagaggctaacaaggccatcataaagaacgcaagggcgct
tggattcaacgttaaggcctttacttacaaagtcaaccttggggaagatgttgaagccaccgaagtctcctccgtggaagagctcgttgctg
aactgctcaacgttgaagggggtatggagtga

>CRISPR-associated protein Cas5

atgaatgttctcctcgttcgcctccgctttccatttttctcggtggcaaggcgctcctatcaggtgagaacatccctcctcctcccaccaccat
catctctaaaaggagcactggcaaagggacttatcctactgaatcctgagaagtatgccagtgattcactcgacaaagccgccagcaaag
ctgtaggggaaattgaaagcaagctcgtgaacgttaaagccgtgagcgtcgctccgataagccccctcgtcaagaacgctttcctgctcaa
acgcctaaggaatctggaatccggctccaaggccgaaaaaggcgacgccatgcgaagggaatacaccttcaccagagagctgctcgtt
gcctatgtcttcaaggacctaactcgagaagagaaggaacgctacctaaaggcggcaatgctcattgacatcgtcggggacacggagag
catagccacgcccctttgggcgtcctttgtggagcccgaagagaaaatagctccccttgccttctcggcaccatactccgaaatctcatcc
ctgctgttcccgaacatacgggcaaagggcaaaataaaggtgtacacggaaagggcatgggtatcgccggattactccagagccataca
ggctaagagaaaaaggggcaagggaaagaaaggcccagaggaggagaccttctacttacccattgaagaacgccgccataagagaac
tgtgtactatacgaggacagtctattctcccaatgttggaaaggcgctgaccattgatggggaggtgcttgggatatggatacccaagaactc
ttccgagagctga

>CRISPR-associated protein Cas8a2/Csa4

gtggttcaggagtttaaggaatctgaaacatttaaaacaccgggtattgacccgatcttcgacctctacgtcgcctacggttacgtggagtcc
cttgtgagaggaggagcaaaggagatcaccctaacacccaagggtatttcgtactcacttcaaacggacatcccagaggaagaatttcga
cgtggactcacagacgcccttgaggagatgctggcactccatgtggcgctggcaagacactctccaagagaaggcggcaagctggttagt
gatgcagacttcagtgcaggagccaacatcaacaatgtgtactgggacagcatacccagaaacctgaaaaaggcaaaagaacgccttg
aagcgggaaaactcccacatgatactgtaaccatgcctataacactcatgccatcggcgggcaagtatatgccgaagcacttcggggca
caaggagggaaccccataaaggttgatctgctcaactatgccctagcctgggtgggattccactattacacaccatacgtaaagtacaca
aaaggcgacaccacgtgggtccacatataccagatagcaccggctgaggaagttgacatgatatcactgctttcccttaaagacctcaag
atggcccttccgcactactacgagaatagcctggactttcttaccaaccgcaggcttgcactcctctatcaccttctccacagcgagagtat
cggagcgctggaggcttttaccaagagggagttcatgatccgctcttacactctcgaaaagtccgggaacaaccaggcggtgcgctccttt
ggtgaggaggaaattggaaggctcatggactttctctatgagctgaaaagaagagatttctaccacacagtgaggttcatcgaaggtctgctt
agggaaaccactgagggagctttggccatgattgacgctataatgaacgaaaggccggagggattctacacggccattaacctcggatgg
aagaagggggtaatccccagccaggagattatcaccactttgggggagatcatcaatgagacttaa

>CRISPR-associated endoribonuclease Cas6 (Ecoli)

atgtatctcagtaaagtcatcattgccagggcctggagcagggatctttaccaacttcaccagggattatggcatttatttccaaacagaccggatgctgctcg
tgattttctttttcatgttgagaagcgaaacacaccagaaggctgtcatgttttattgcagtcagcgcaaatgcctgtttcaactgccgttgcgacagtcattaaa
actaaacaggttgaatttcaacttcaggttggtgttccactctattttcggcttcgggcaaatccgatcaaaactattctcgacaatcaaaagcgcctggacagt
aaagggaatattaaacgctgtcgggttccgttaataaaagaagcagaacaaatcgcgtggttgcaacgtaaattgggcaatgcggcgcgcgttgaagatgt
gcatcccatatcggaacggccacagtatttttctggtgatggtaaaagtggaaagatccaaacggtttgctttgaaggtgtgctcaccatcaacgacgcgcc
agcgttaatagatcttgtacagcaaggtattgggccagctaaatcgatgggatgtggcttgctatctttggctccactgtga

>Cas6e-HPV16-guide

CCATGGATTAATAATGAGAGTTCCCCGCGCCAGCGGGGATTGAAACattaaaaaacctaacaataacaaaatatt
agttcctaGAGTTCCCCGCGCCAGCGGGGATTGAAACattaaaaaacctaacaataacaaaatattagttcctaGAGTTCC
CCGCGCCAGCGGGGATTGAAACattaaaaaacctaacaataacaaaatattagttcctaGAGTTCCCCGCGCCAGCG
GGGATTGAAACattaaaaaacctaacaataacaaaatattagttcctaGAGTTCCCCGCGCCAGCGGGGATTGAAACa
ttaaaaaacctaacaataacaaaatattagttcctaGAGTTCCCCGCGCCAGCGGGGATTGAAACattaaaaaacctaacaataac
aaaatattagttcctaGAGTTCCCCGCGCCAGCGGGGATTGAAACattaaaaaacctaacaataacaaaatattagttcctaGA
GTTCCCCGCGCCAGCGGGGGTCGAC

>Cas6e-HPV18-guide

CCATGGATTAATAATGAGAGTTCCCCGCGCCAGCGGGGATTGAAACctgctattggggaacactgggctaaagg
cactgcttgGAGTTCCCCGCGCCAGCGGGGATTGAAACctgctattggggaacactgggctaaaggcactgcttgGAGTT
CCCCGCGCCAGCGGGGATTGAAACctgctattggggaacactgggctaaaggcactgcttgGAGTTCCCCGCGCCA
GCGGGGATTGAAACctgctattggggaacactgggctaaaggcactgcttgGAGTTCCCCGCGCCAGCGGGGATTG
AAACctgctattggggaacactgggctaaaggcactgcttgGAGTTCCCCGCGCCAGCGGGGATTGAAACctgctattggg
gaacactgggctaaaggcactgcttgGAGTTCCCCGCGCCAGCGGGGATTGAAACctgctattggggaacactgggctaaag
gcactgcttgGAGTTCCCCGCGCCAGCGGGGGTCGAC

>Cas6e-Target DNA-guide

CCATGGATTAATAATGAGAGTTCCCCGCGCCAGCGGGGATTGAAACagtgcttccccaaacccttaactggttgt
aacagttgGAGTTCCCCGCGCCAGCGGGGATTGAAACagtgcttccccaaacccttaactggttgtaacagttgGAGTTCC
CCGCGCCAGCGGGGATTGAAACagtgcttccccaaacccttaactggttgtaacagttgGAGTTCCCCGCGCCAGCG
GGGATTGAAACagtgcttccccaaacccttaactggttgtaacagttgGAGTTCCCCGCGCCAGCGGGGATTGAAACa
gtgcttccccaaacccttaactggttgtaacagttgGAGTTCCCCGCGCCAGCGGGGATTGAAACagtgcttccccaaacccta
actggttgtaacagttgGAGTTCCCCGCGCCAGCGGGGATTGAAACagtgcttccccaaacccttaactggttgtaacagttgG
AGTTCCCCGCGCCAGCGGGGGTCGAC

>hypothetical protein (Cas11)

atggatgagtgggtcaaaaatattggcaggtacctgagctacctcgtggatgagacgtttgaggaatacgcctacgacattgttgacagcatagcaaaggc
aagaatccaggaggagcttctcgaaggtgtctacaaggccctcaggcttgctccgaagctcaagaagaaggccgattctaaaggctgcttcttccaaaaac
cttcccccaaggacatcgaagcactcgaagacagggttgaggagctgtccaacactaaggacctcaggaaactggcggtaagcttggccctttgggcctt
tgcctactggaaccactgcccgaaaaaggagaacaaaactgaaggaggtgttgaatga

>CRISPR-associated endonuclease Cas3-HD

atgagctgtgaagcattcaggggacaaacactgagacaacacgttgatgcgatgctctccgcatgggagagtgttaagagcaaatatatcccctcgatcat
cagagcaatgagggcagttggaattgaattcaccgaagaagatgccaacaggttcatgaaagccatcataatactgcacgacaccggaaagtgtagtgat
gtataccagagacacttaaagactggagaatcactgaaaggattcagacatgaactcgtaagcgcttattatgcacacgggattctgaaagagatattcaat
gaagaagtggctttcattggggcccttgtggtcatgatgcaccatgagccaattttaatggggcagatacgctcccttgacagggaggagctctcccccga
ggttgtcctcgacaagctccgcaacttcaacggagttgtggaggatacagagcccttcataaagagcatgatgaaagagaagcttggaatgattccagagg
tcccatcccccacccgggaagacgttcttggggaagtcatcaggcttagcgtgctcgccagacacagacccgactctgacaagttgagaatggtggtcgg

agcactgctcattccgctggttctctgtgactacaagggggccgaggaaagagagggcgaagccccaaagttcgccgaggtccttggagtcgagatgat
gctgtga

>CRISPR-associated endonuclease/helicase Cas3

atggatacccaagaactcttccgagagctgaccggctttgagccatacgattaccagattcgcgcgtggaagaacatcgagaaaatcatggaaaacggcg
gaaaggccgttatcgaagttcccacggcgggaggaaagactgaaaccgctgtaatgccgttcctctctggagtctatcacaacagctggcccgttgccag
gctgatctacgtcctgccaacgagatcactcgtggagaagcaggcggacagagtgaggaacctggtttctgggcttcttcagctgaaaggaaaatcgggg
gaagaggcgaaaaagctcgccagggaagttgttatcgttgaatacggtcttgagaaaacccacgcgttcctcggctggatagttgtgataacgtgggatgc
gttcctctatggccttgcagcccacaggacagtcgggaacaggttcacattcccagcgggggcaatagctcagtctctcgtcgtatttgatgagattcagatg
tatcaggacgaatcaatgtacatgccacgccttctttcacttgtcgttggaattctcgaaaaggccaacgtgccggtcattgtaacgagcgcaactattccttca
aagctgagagagatgatagtagagggcacggaagtcataacagtcgaagacggggacaaaaacaagccgtcaaggggggaacgtgaaggtcagagta
gttgagggagatactgcgggagttctcaatgacattaaaaaggccctggaagaaggaagaaaagttctggttgtgcgtaacacagttaggaaagccgttga
aacttaccagtttctgaaaggtgagctacacgacatcctgattgatccctcggatgcgctgctcatacacagcaggttcacagttggcgacagaagagaga
aagagagagctcttgactcagcaaggctgatcgttgcgactcaggtggtggaggctggactcgacctgccgaacgtgggattggtagtaaccgatatcgc
accactcgatgccctcatccagcgcattggtagatgcgcaagacggccaggagaggaaggagagggcatagtactcctaccggtggaagatgacattg
agtccgaaaaggtcgtgaggggactttcggagctgaagaaaaaggtaggtaaagacagtgtcgtctttgcaactgttacaaacacaaaggagtatggcag
ggttgcggaggtccattacggagaagacaagaaggattctgtgcacgttggtgacatcaacctcgccagaaaaatcctaggggaaaagaagggaagaa
gaaatccaaaactgcccaaggacctttacataataccctactcaacggctccctacgacccgctggtgcttctcacgacatatgacgagctatcaaatatcga
ggagtatctaaacaacacggcaaaagcaagggaagcgctcgataaggtttaccgcttccactacgagaacaacatcataccgagggagtttgcctccgcc
tatatctacttcaaggagctgaaactgttctcagctccgcccgaatacgagctccgctccagaccagagctctacatgatgttgtatccaacaagcgagaag
aagatggaaaaacttaacatgaacagaatcattaggatcagctacgacaggaacaggctctacgcatggaaaaaggatgaaatcatcgttggaaggctca
gagagatatggggcgaaaccagcagggatgtagcaggttgggaagtccagaaaacattcacaccaagcccctacggtatatatgcaatgaacccaagct
attatactcaagaactcggttttgtgcctggcactctcggttccaaagatgatatcgacacatcaaatgaaagagggaactcgaactcaaaagaaccatcgg
aagattccacagagaagtcagcaggaaatacctccaagaaaatgggggatcaaggtaaacaagtgagtctc
gaaagatggggggtgagagtatga

Homologue search for based on *Pfu*Cas3HD:

Gene ID	Locus Tag	Product Name	Scaffold ID	Genome
638162626	SSO1403	CRISPR-associated nuclease, Cas3 family	AE006641	Saccharolobus solfataricus P2
638169912	PAE0207	CRISPR-associated nuclease, Cas3 family	AE009441	Pyrobaculum aerophilum IM2
638173162	PF0639	CRISPR-associated nuclease, Cas3 family	AE009950	Pyrococcus furiosus DSM 3638
638201689	MJ0384	CRISPR-associated nuclease, Cas3 family	NC_000909	Methanocaldococcus jannaschii DSM 2661
641613515	Kcr_0434	CRISPR-associated nuclease, Cas3 family	NC_010482	Candidatus Korarchaeum cryptofilum OPF8
641668284	Tneu_1133	CRISPR-associated HD domain protein	NC_010525	Pyrobaculum neutrophilum V24Sta
643828671	M1425_0862	CRISPR-associated nuclease, Cas3 family	NC_012588	Sulfolobus islandicus M.14.25
643842666	M1627_0927	CRISPR-associated nuclease, Cas3 family	NC_012632	Sulfolobus islandicus M.16.27
644808596	TGAM_1295	CRISPR-associated nuclease, Cas3 family	NC_012804	Thermococcus gammatolerans EJ3
646418632	Tcur_0935	CRISPR-associated helicase, Cas3 family	NC_013510	Thermomonospora curvata DSM 43183
646612990	Ferp_1863	CRISPR-associated nuclease, Cas3 family	NC_013849	Ferroglobus placidus AEDII12DO, DSM 10642
646944875	Ssol_2280	CRISPR-associated nuclease, Cas3 family	CP001800	Saccharolobus solfataricus 98/2
650023445	Ssol98_010100000235	CRISPR-associated nuclease, Cas3 family	NZ_ACUK01000006	Saccharolobus solfataricus 98/2
650831767	PNA2_1817	CRISPR-associated nuclease, Cas3 family	NC_015474	Pyrococcus sp. NA2
2264867995	A471O8DRAFT_00996	CRISPR-associated helicase Cas3/CRISPR-associated		

endonuclease Cas3-HD A471O8DRAFT_contig_11_0.12 Crenarchaeota archaeon SCGC AAA471-O08 (contamination screened)
2264868846 A471B5DRAFT_00560 CRISPR-associated helicase Cas3/CRISPR-associated endonuclease Cas3-HD A471B5DRAFT_contig_7_0.8 Crenarchaeota archaeon SCGC AAA471-B05 (contamination screened)
2264870194 A471B23DRAFT_00951 CRISPR-associated helicase Cas3/CRISPR-associated endonuclease Cas3-HD A471B23DRAFT_contig_26_0.27 Crenarchaeota archaeon SCGC AAA471-B23 (contamination screened)
2264896305 A3IWDRAFT_00522 CRISPR-associated endonuclease Cas3-HD A3IWDRAFT_NODE-unique_42_len_2818.42 Crenarchaeota archaeon SCGC AAA261-G18 (unscreened)
2265120379 A471L13DRAFT_00465 CRISPR-associated helicase Cas3/CRISPR-associated endonuclease Cas3-HD A471L13DRAFT_contig_8_0.9 Crenarchaeota archaeon SCGC AAA471-L13 (contamination screened)
2265121156 A471L14DRAFT_00233 CRISPR-associated endonuclease Cas3-HD A471L14DRAFT_contig_3_0.4 Crenarchaeota archaeon SCGC AAA471-L14 (contamination screened)
2504153610 Desac_0091 CRISPR-associated endonuclease Cas3-HD Dace11109_unknown1 Desulfobacca acetoxidans ASRB2, DSM 11109
2511627530 TTX_1254 CRISPR-associated nuclease, Cas3 family NC_016070 Thermoproteus tenax Kra1
2512378622 Pogu_1144 CRISPR-associated endonuclease Cas3-HD CP003316 Pyrobaculum oguniense TE7, DSM 13380
2517227900 PFC_02330 CRISPR-associated nuclease, Cas3 family CP003685 Pyrococcus furiosus COM1
2522052361 Py04_0801 CRISPR-associated nuclease, Cas3 family CP003534 Pyrococcus sp. ST04
2523845491 H528DRAFT_01836 CRISPR-associated helicase, Cas3 family H528DRAFT_scaffold00025.25 Thermodesulfatator atlanticus DSM 21156
2524568206 NAG2ff85r04_01613 CRISPR-associated nuclease, Cas3 family NAG2ff85r04_contig00342.68 NAG2_ff85-r04
2527531862 YNPFFACOM1_01266 CRISPR-associated helicase Cas3/CRISPR-associated endonuclease Cas3-HD YNPFFACOM1_contig_13_0.14 Crenarchaeota archaeon SCGC AAA471-B05 (contamination screened) (contamination screened) v2
2533542706 CRISPR-associated endonuclease/helicase Cas3 CAOS01000006 Desulforamulus hydrothermalis Lam5
2549767849 M1612DRAFT_00985 CRISPR-associated nuclease, Cas3 family M1612DRAFT_AHJL01000001_1.1 Sulfolobus islandicus M.16.12
2549770957 M1613DRAFT_00958 CRISPR-associated nuclease, Cas3 family M1613DRAFT_AHJM01000001_1.1 Sulfolobus islandicus M.16.13
2549774010 M1622DRAFT_00976 CRISPR-associated nuclease, Cas3 family M1622DRAFT_AHJN01000001_1.1 Sulfolobus islandicus M.16.22
2549777118 M1630DRAFT_00957 CRISPR-associated nuclease, Cas3 family M1630DRAFT_AHJP01000001_1.1 Sulfolobus islandicus M.16.30
2549780190 M1646DRAFT_01038 CRISPR-associated nuclease, Cas3 family M1646DRAFT_AHJS01000001_1.1 Sulfolobus islandicus M.16.46
2559588888 OMM_04547 CRISPR-associated endonuclease Cas3-HD ATBP01000943 Candidatus Magnetoglobus multicellularis Araruama
2587762900 EJ92DRAFT_02629 CRISPR-associated endonuclease/helicase Cas3 EJ92DRAFT_scaffold00034.34 Desulforamulus hydrothermalis Lam5
2587808271 EJ24DRAFT_01427 CRISPR-associated endonuclease/helicase Cas3 EJ24DRAFT_scaffold00031.31 Caloranaerobacter azorensis DSM 13643
2588252189 EJ28DRAFT_01465 CRISPR-associated endonuclease/helicase Cas3 EJ28DRAFT_scaffold00009.9 Caminicella sporogenes DSM 14501
2620073532 Ga0070342_10331 CRISPR-associated endonuclease Cas3-HD Ga0070342_1033 Aigarchaeota archaeon JGI MDM2 JNZ-1-H10 (unscreened)
2621225351 Ga0061022_102028 CRISPR-associated endonuclease/helicase Cas3

Ga0061022_1020 Caloranaerobacter azorensis H53214
2636760083 Ga0081690_11484 CRISPR-associated nuclease, Cas3 family Ga0081690_11 Saccharolobus solfataricus 98/2 SULC
2637142231 Ga0081679_11484 CRISPR-associated nuclease, Cas3 family Ga0081679_11 Saccharolobus solfataricus SULB
2643433219 Ga0097913_10255 hypothetical protein Ga0097913_1025 unclassified "LHC4-2-B" archaeon JGI MDM2 LHC4sed-1-B14 (unscreened)
2648418494 Ga0097871_10223 CRISPR-associated helicase Cas3/CRISPR-associated endonuclease Cas3-HD Ga0097871_102 unclassified Euryarchaeota archaeon JGI MDM2 SSWsed-3-L11 (unscreened)
2649207289 Ga0081668_11486 CRISPR-associated nuclease, Cas3 family Ga0081668_11 Saccharolobus solfataricus SULA
2657030176 Ga0071124_1523 CRISPR-associated nuclease, Cas3 family Ga0071124_12 Thermococcus thioreducens OGL-20
2682505067 Ga0128351_101735 CRISPR/Cas system-associated endonuclease Cas3-HD Ga0128351_101 Vulcanisaeta distributa JCM 11213
2682523895 Ga0128321_10830 CRISPR/Cas system-associated endonuclease Cas3-HD Ga0128321_108 Vulcanisaeta souniana JCM 11219
2682526968 Ga0128353_10275 CRISPR-associated endonuclease Cas3-HD Ga0128353_102 Thermococcus sp. JCM 11816
2682526969 Ga0128353_10276 CRISPR-associated endonuclease Cas3-HD Ga0128353_102 Thermococcus sp. JCM 11816
2682537477 Ga0128371_102288 CRISPR-associated endonuclease Cas3-HD Ga0128371_102 Vulcanisaeta sp. JCM 16159
2682544996 Ga0128346_11021 CRISPR-associated nuclease, Cas3 family Ga0128346_110 Sulfolobus sp. JCM 16833
2682561660 Ga0128453_100555 CRISPR-associated endonuclease/helicase Cas3 Ga0128453_10055 Thermobifida fusca NBRC 14071, JCM 3263
2684057382 Ga0124707_10678 CRISPR-associated nuclease, Cas3 family Ga0124707_106 Thermococcus thioreducens DSM 14981
2688750946 Ga0133540_111254 CRISPR-associated helicase, Cas3 family Ga0133540_11 Methanoculleus bourgensis MBBA
2689041556 Ga0133342_11786 CRISPR-associated nuclease, Cas3 family Ga0133342_11 Pyrococcus kukulkanii NCB100
2714875563 Ga0125334_10114 CRISPR-associated helicase, Cas3 family Ga0125334_101 Desulfolucianica intricata NBRC 109411
2717094174 Ga0170752_1046 CRISPR-associated endonuclease Cas3-HD Ga0170752_104 Thaumarchaeota archaeon SCGC AD-291-D14
2724283233 Ga0181714_10904 hypothetical protein Ga0181714_1090 Unclassified archaeon JZ bin_50
2724283847 Ga0181712_11123 CRISPR-associated helicase, Cas3 family Ga0181712_111 Unclassified archaeon DRTY7 bin_35
2729863776 Ga0181037_105274 CRISPR-associated Cas3 family helicase Ga0181037_105 Allonocardiopsis opalescens DSM 45601
2730041368 Ga0180936_10651 CRISPR-associated nuclease, Cas3 family Ga0180936_1065 Archaeoglobus sp. JdFR-32
2730044867 Ga0180935_10143 CRISPR-associated nuclease, Cas3 family Ga0180935_1014 Archaeoglobus sp. JdFR-31
2730674046 Ga0180975_10517 CRISPR-associated Cas3 family helicase Ga0180975_105 Actinomadura viridilutea DSM 44433
2730742759 Ga0036909_11970 CRISPR-associated nuclease, Cas3 family Ga0036909_11 Sulfolobus islandicus M.16.47
2730768369 Ga0173049_11410 CRISPR-associated nuclease, Cas3 family Ga0173049_11 Saccharolobus solfataricus P1
2732265458 Ga0128527_100911 CRISPR-associated endonuclease/helicase Cas3 Ga0128527_1009 Actinomadura rubrobrunea NBRC 15275
2752495578 Ga0213453_10323 CRISPR-associated helicase Cas3/CRISPR-associated

endonuclease Cas3-HD Ga0213453_1032 Unclassified Geoarchaeota GBS-NAG1
2755814153 Ga0213556_13822 CRISPR-associated Cas3 family helicase Ga0213556_138 Halanaerobium saccharolyticum WC1
2758667978 Ga0226574_111484 CRISPR-associated Cas3 family nuclease Ga0226574_11 Thermococcus siculi RG-20
2771144415 Ga0254923_10253 CRISPR-associated endonuclease Cas3-HD Ga0254923_1025 Caldivirga sp. Obs2_genome_027 Obs2_genome_027
2771197259 Ga0254949_1178 CRISPR-associated Cas3 family nuclease Ga0254949_117 unclassified Archaeon Obs3_genome_053 Obs3_genome_053
2774029930 Ga0263258_11175 CRISPR-associated Cas3 family nuclease Ga0263258_111 Unclassified Archaeoglobales GMQP bin_32
2775399572 Ga0226170_111640 CRISPR-associated Cas3 family nuclease Ga0226170_11 Thermococcus thioreducens OGL-20P
2807056317 Ga0314309_129625 CRISPR-associated Cas3 family helicase Ga0314309_1296 Thermobifida halotolerans DSM 44931
2807325993 Ga0154130_118524 CRISPR-associated Cas3 family helicase Ga0154130_1185 Paenibacillus darwinianus CE1
2828765395 Ga0392504_448 CRISPR-associated endonuclease Cas3-HD Ga0392504_02 Thermococcus sp. LMOA06
2828768751 Ga0392521_1638 CRISPR-associated endonuclease Cas3-HD Ga0392521_01 Thermococcus sp. LMOA07
2829975503 Ga0373264_988 CRISPR-associated endonuclease/helicase Cas3 Ga0373264_01 Spirosoma lacussanchae DSM 101771
2830813125 Ga0394040_2807 CRISPR-associated endonuclease/helicase Cas3 Ga0394040_351 Obscuribacterales bacterium Service_water_21
2831516021 Ga0365486_1337 CRISPR-associated endonuclease/helicase Cas3 Ga0365486_12 Caminicella sporogenes AM 1114
2839666852 Ga0335788_2663 CRISPR-associated endonuclease/helicase Cas3 Ga0335788_18 Thermobifida halotolerans YIM 90462
2904614814 Ga0450666_06_78715_81162 CRISPR-associated endonuclease/helicase Cas3 Ga0450666_06 Fervidibacter sacchari PD1
2913344159 Ga0451167_03_44250_44966 CRISPR-associated endonuclease Cas3-HD Ga0451167_03 Thermococcus stetteri DSM 5262
2923402884 Ga0039428_01_777900_778748 CRISPR/Cas system-associated endonuclease Cas3-HD Ga0039428_01 Aeropyrum pernix K1
2923984953 Ga0477847_59_25057_27366 CRISPR-associated endonuclease/helicase Cas3 Ga0477847_59 Parabacteroides sp. 52
2940372979 Ga0505229_12_24308_26617 CRISPR-associated endonuclease/helicase Cas3 Ga0505229_12 Parabacteroides sp. PM5-20
2989478066 Ga0487045_15_208126_211140 CRISPR-associated endonuclease/helicase Cas3 Ga0487045_15 Nonomuraea glycinis CGMCC 4.7430
2999171050 Ga0490602_01_2047145_2047654 CRISPR-associated endonuclease Cas3-HD Ga0490602_01 Candidatus Atribacteria bacterium RT761

Sequence alignment:

>2807325993.Ga0154130_118524

------------------------MK--------QRI--------LAKGVSKEQHVPAFATLAGHAANVADAGDKLARWL
GGGALR-HFGLAD-RESDLRMVLRWFGWTHDWGKAGNQ--FQQMLQG------G---
NPLQLMRHEVVSALLLRH-----
-----HPVL--S--RLTEAL---GKELANALLCAVLGHHRKFPAPYQMADIRER--------------E--DLS------
----------------V------------------D--INHSDFREMLTAMYR---------------------------DL
GLSEPMGEIEPLKITKHSVTHGTLDYWWADDKLV----RLKDDLADWWEQN---
KTDEPVRFIALSKAFAIAADVAGSAL
PDPEERTQLIRSASETGITPDDMHGL-------IERWAKNRGI--------P-TPFRPRDFQRRVA----------ESES

RLTLVEAGCGSGKSVAAYMWAQRWAERFKTDGHSPPHFFFCMPTMGTATEQFRDYAM---
----NADVDAILEHSKANLDLDSIRNFLTAEEGESDTER---------FIRQESTIEAFR----------
LWSAPMIVCT
ADTVLGSMVNARK-------------SLYHFPVVANGLIVFDEVHAMDNRV---FRHLLIFLRHM--------
PHLPVLL
MTASLSEYRRQMLTEARQEPMAVIS---------DRES---GQAELPRYWLNRVD------------
DKRPLTDAISRTV
AA--G-----G-NVLWVCNQVERAAENYEEMQKRFGRE----
GVRVFVYHSRFRYGDRSKLHRRAVDSF-------GDGQ
GPVILIATQVAEMSLDLSADLLVTDLAPIPSLIQRLGRLNRRALPEHRPEPKTAYILKP----------ESS----
----
------------L----------------------------PYKDEELEQAAKWIASLQSGERSLA-QRDLAQVFEQMEG
---SVDRNPRLEWREA-----FKNAVFFSGKWETPVHVLRETGHTVSIILENDLRGFEAKQG----REP-----
------
-----G-RDWIG------------------------------EHEVPILLKSEVF-----SWKRIAGRP------------
------IA--
-P------------------------------VERIHYDYDESTGRGRGAAWVD------------------------------
----

>2758667978.Ga0226574_111484

------------------------------------MSCE--------AFRGQTLRQHVDAMLSAWES------VKSKYIPSI
-IRAMR-AVGIEF-TEEDANRFMKAIIILHDTGKCSDV--YQRHL-K------TG--
ESLKGFRHELVSAYYAHG-----
-----I--L--K--EIFNE----EVAFIG-A-LVVMMHHE----PILMGQIRSL--------------DREELSPE----
-------VVLDKL-RNF--------------------N--GVVEDTEPFIKSMMK------------------------EKL
GMI---------------PE------------VP----SP--------------TREDVLGE-------VIRLSVLARHR
PDSDKLRM-VVGALLIPLVLCDYKGA------------EEREG-------------EAPKFAEVL-----------GVEM
--------------------ML---

>2714875563.Ga0125334_10114

----------------------------M---ISFYAKP--------DQTYREHIEDVYSAWQETVA---SKKQLIERM
----SR-Q--YGF-SVNRFLKGSLLTVVLHDIGKMSNT--FQAMMKALRDNKKFN---
YKNNYRHEILSFVLTVM-----
-ASLELSKK---DGVL-------TQ--IPVEALAVAGHHR----PLNTDLTSFERESRSP-------------VP----
------ALDPYGLRVA-------------------------------L-------------------------VLAEEI
FA-REGWNFPTIDEKAVQ-E----NSYKRLAQLV----DPY---NNLIGKL-------------------------LDR
DGYEKARD-LYVLVKGILHYADWHGSGKAKVNYFI---EKP------PER-----LEEE-LAKRC---
QEKKISFTGLRP
FQKSMGQC-----------SGHLLAVAPTGSGKTEGSLLWALKNIQEM-----
DGAKIIYLLPTMNTANQIWERLV-KMF
---G--IENVGLTHSTANLFL--------AEESEEES---W------------------ADRRDL-LFHQSFIKPVTVAT
VDQWLTCGFNAGRWVL------------KEI-NASNAVIILDEVHSYDGWTLGLIISAIRHFT-A--------
RGSRFLL
MSATMPQGLVKLFENVLSDIKVIK--E-----E--------NLLSASRSVYKVVDAPIE------------------EAE
KTIYEAVSAGR-KVLVVVNTVEKCQQLAEHF--A--------GLNPVCYHSRFILKHRKEIEEGL------------
--D
QSRFVIATQVIEVSLDIDFDWLFTECAPPDAIAQRAGRVNRYRDRDRDSRVLIYQPSNQ-----------
SGKIYNPLD-
-NPQLLKRSYKE-FQNRQGLLCER------DLIEIIEK---VYTGYRID---KTEP-FQVAID------IYR--------
------------QARKRRMAIFDSRLDEDEQE---------------------VT-RQSEYD----TVSVIPYCFYE---
-----------EVINLSPW----------------ERKWYE-----VKVPLWYF----------------IKNK--KALN
S--GLCFCDLEYVRQIGALLKPAKSKVALW---

>2264870194.A471B23DRAFT_00951

--------MTLNSIY--LELCNERAW------------------------------KPRYFIEETLN-------------
---KLENL------LKENKRFVLIARLPTGYGKTTITNVLARAALKN----
NPYFCRVIHVLPMRSIADDVCLELQK---
--------DKM---ISNHVAVQHLLT--PGSPYFAKKCIVTT
LDTFLLNFFKIPTPELKKVFQYDTAHSEFPRAMIYLSIVIFDEFHLFAGLG-
DISNEGKALTSVIASMICLLKAKVPIII
MGAT----IPDVLIDKIREEISIVG----------------GEVESVNYVFDKDLEFDEK-----LRKKKKRIHIEEKDV
MEILNNIDSSK-SVLIVVNTVKKAKEIYNSITNKEEVG---------FLHGKIPEA-----IRAEIIKK-------
IKNS
KPRILIATQVIEAGVNISYDILITEPCPIDRLIQRAGRVCRFDEEEGD-----IYISKF----------
EKDYIYDENF-
-----VKTTLEVLNPNKGIERKES------NVLTF------EFANQAINEVYKI------------PIKELKEIDYDMKG
FLQELDALPILTSKYAK-------------------------------ELIIRYKGFTENFG----IVSCFNEKYLNKLY
AIPVSEKEGWELIKKHGKL-----IDANYRLIDFKAKLR-EVESLSIYL----------LDKGYQGIA------------
------
IPEEEYIEIIGFRPKIDIKTSASASKNISSSPSHHEISKPCAFIGQSLIKHTENTLEKALKMKYSFETMSK
RFE
ALGININAERLEKLIKAACILHDLGKAADEYQEDFYNSCECKG--
KVSFYLHEVASAYYAYKKLKSIEDYELDTKERQLI
TLAILFHIAGKNLYDLKDGVKEKKMKWTFNKYWKSFEHLLNKHRIKVDYKSSSINTEEAMNFLEE
IEEIIRKRESYYLKL
YNLIYSAICVGDNLDSYEFRRTDISESRKIFIEELKEVIANA
>2657030176.Ga0071124_1523

------------------------------MSLL--------AFQGQTLRKHVNAMLNAWES------VKGKYIPSI
-IRAMK-AWGVEL-SREDADRLMKALIILHDSGKGAEL--YQDYL-E------GK--
ARLSGFRHELVSAYYALK-----
-----I--L--P--QIFDE----KTAFVG-S-LVVMLHHE----PILMGQVANL--------------DRDSLSAE----
-------VALDRL-KNF------------------D--GVVPELNEFLENSFR------------------------EHL
GVE---------------IT-----------VL----SA--------------SPDEVVRA-------VVELSVRARHL
PDAGRLRL-IVGALLIPLVLCDYKGA------------EEREG------------ETPKFAEVL-----------EAEW
------------------LGVV---

>2549767849.M1612DRAFT_00985

--------------------------MT----CWA--------FYGMETFKQHSLGILNFFRDN-------FSYII--
PIISY--RTGI---DKETVRKSVEIGVSLHDIGKTSKH-------------------YDRSYFGHEFYGGYLVYK-----
-ILRECCD-----SE-----------LKPLVALAAMSHHQ----GMGGRTLNEMILKGNYT------------------
---RI----------PPTYELREECR--NDILKILVEI
GVE---------------IKD--------FPQKV----TR-SDVKSWFQKL-------NIKW------------------
-------KNLYVIILGPLMVSDTVVA-----------NKNRGG--------N-QYNRI--IEEYE-----------KWIN
------------------------VQ---

>2807056317.Ga0314309_129625

------------------------VNPL---NRIWAKSASP-GQSWGELLTEHLDATLTALDLLRH----------RV
----GR-I-A--A-VPDRFWTWAALACLFHDAGKIPEG--FQRMVGNPR----PA---
QVWGLRHEIYSLGFVDH-----
-VLAHLDED---E-------------RQWIALGVLTHHR----PLSGGARSIRKQKGSLR-------------TP----
------QAVTDAFG-----------------------PVDEQTANALTA-------------------------WLAQRC
YA-------PV------PKPVTATDLGKATHRLL----TTV--LDHWAE-----------------------------E
SPDNEAGL-HAVLLQGAVTLADHVASAHTTLLTD------------HPLD--AEYPER-LRK------

RLTNQGATLFP
HQEAAARA-----------
SGHLLLRAPTGKGKTEASLLWALTQIDQVRATTGGQPRLFYTLPYLASINAMADRLGEELD
DPE---RQSIGVTHSKAADYH--------LRRAINDDHDETEPLEH-----------ATRAVAKANA-
SRLFRELVRVTT
PYQLMRAALAGPAHSS------------TLI-DSVNSVFVFDELHAYDTHRLGIILAMTGMWA-R--------
LGGRIGV
VSATLPDALAELIEKTLGEPLAEV--A-----PDS------GQAWPRRHRLHLDETHLT----------------STESI
AAITEQLVQGN-SVLVVANNVADAQNIYDTLAPTARELYGD--
DGAILLHARFRAKDRGEIERRILERYGT-----NQKH
HPGLVVATQVVEVSLDLDFDILHTSAAPLEALIQRFGRVNRLNGRDQ-
SAPVVVHRPDYAPRARGGGDEYADKVYAAEP-
-----TRLGWDILIRHDGDPLDER------LFTDWLNE---VYTSPWGQ---RWRSDVERVRT------EFT--------
----------------RRFLTFDPPFDDRSDL---------------------AAAFDQMFD----GAEGILAEDLE---
-----------AYREALDQGR----DPQT-----RKAARLLASGYLISLPDH------------------ARRL--GRWD
KEFGLIVIDADYTEEKGLGALNRDDRTSYVMGEVL---

>2724283233.Ga0181714_10904

--MTRWRCEMEN---------
-----------CP-LRGVSKVCGDGKDGPVVVSYHPS--------NGNKECYVRHSVLCWEEWKR------
VFDRYIRSI
-HRVFRKTLFINV-DLKVIENICGVAVLHHDVGKLCAD--YQSA----------------RFYRHEMISSFLIHD---
--
-----YVVGMLKNSSLDEEK---AELLSSIVSAAVYLHHEGLQ----IS---HE--------------YYEMRAPT----
-YGYL--LNLLAG-REF------------------R---MVESWMLISSELEK------------------------YAF
GR--------------------TLGYFANV---S----SV--------------SGYEVTNVLG-------SVITLVDGA
PDPLPLRL-AVASILHPITITDNLAS------------QKRGG-------------KPSLLSSFLG----------VTER

>2682526969.Ga0128353_10276

---MLAAWER------VKDKYIPSM
-IRAMR-AVGIEF-TEEDADRFMRTLIILHDVGKCSDI--YQKHL-N------NN--
EPLRGFRHELVSAYYAHK-----
-----I--L--K--EVFKDE---NVAFLGGA-LVVMMHHE----PILMGQIRSL--------------DRDELSPL----
-------R---

>2682537477.Ga0128371_102288

------------------------------MGACYSY-N---KE--GKVERYEDHIKAAVAVWR-I-----IKPYYGKVL
-DRVL----------GKLKFDPIAVSLAVHDLGKLTNA--YEKS---------------PRDFRHEIFSGYAMYK-----
-ILEKT-----------------PEDVRMALSLAVTLHHE----NILI-GVY----VGKLGERY----------------
--------------------------------------LTISNIKRILNIFKDELKPNCDVKQ--------TFNELLKNF
LVR------------EGFVND----E-----LTN----YVMGFIDHWMNDGVK--IDDIIYSIKD-----VIAWASVGPL
EDLLIRRA-KVASVIHLITLTDSIAANVLRG------GGMEDE--------G-NWIVERVSSGAE---------
PVSIDE
VKNAINKVV-------------GNAP--

>2549770957.M1613DRAFT_00958

----------------------------MT----CWA--------FYGMETFKQHSLGILNFFRDN-------FSYII--
PIISY--RTGI---DKETVRKSVEIGVSLHDIGKTSKH-------------------YDRSYFGHEFYGGYLVYK-----
-ILRECCD-----SE-----------LKPLVALAAMSHHQ----GMGGRTLNEMILKGNYT-------------------
---RI----------PPTYELREECR--NDILKILVEI
GVE---------------IKD--------FPQKV----TR-SDVKSWFQKL-------NIKW------------------
-------KNLYVIILGPLMVSDTVVA-----------NKNRGG--------N-QYNRI--IEEYE-----------KWIN
--------------------------VQ--

>2828768751.Ga0392521_1638

---------------------------------MTLL--------AFRGQTLEEHVEAMLQAWEE------VKGKYIPSI
-IRAMK-AYGIEL-DWDEADRLMRALIILHDSGKGARL--YQDYL-R------GG--
EKLRGFRHELVSAYYALK-----
-----I--L--P--QLFGE----RVAFVG-S-LAVMLHHE----PILMGQVASL--------------DRDSLSAE----
-------VALDKL-RNF------------------D--GVVPELEEFLRESFR------------------------KHL
GAD---------------VD------------VP----GA--------------EAGDVVGA-------VVELSVKARHL
PDAGKLRL-IVGALLIPLVLCDYRGA------------EEREG-------------EAPKFAEVL-----------EAEW
--------------------LGVV--

>2517227900.PFC_02330

---------------------------------MSCK--------AFQGQTLREHIEAMLAAWEI------VKNKYIPSI
-IRVMK-TVGVKF-TEEDADKFMKTLIILHDVGKCSEV--YQKHL-S------NN--
EPLRGFRHELVSAYYAYN-----
-----I--L--K--DMFKDE---TIAFIG-A-LVVMMHHE----PILMGQIRSL--------------DKEELTPE----
-------VVLDKL-RTF------------------N--GVMEGTESFIKSMIK------------------------EKL
GVI---------------PK------------VP----SP--------------TQEDVLRE-------VIRLSVLARHR
PDSGKLRM-VVGALLIPLVLCDYKGA------------KEREG-------------ESPKFAEVL-----------RVEM
--------------------MK--

>2724283847.Ga0181712_11123

------------------------------MQHDQLL--------ARPNQKLIDHLREI-------------KNKI----
---------------PKNISELEALVFLLHDIGKVSDK--FQKKLEK------N----ENFRFRHPLASLPIIEY-----
-IIKEYNIT---NQRSI------NEEDIDIYLMLVYYHHG----PISFDMLQNDLVHFSIQKNNINIIEKDELIRV-
---

-KQILQSLDPNYG-KYFDEEKFIYYYLNSNGSDYNR--LYNEKLKEILKRIYN-----------------------K-F
KMV-----------------------------SP----EVKKKF---------------------------------------
-----------ASLYSIFVELDWYSTSDSNKEILPDWNKIESS-------------FKKKFKERKEERVD---KDLEAES
IRNKIEDLL-----IKNIDDDRIFICAPTGIGKTELSLKWALSNAKKLN-----
ARKIIYVLPYRNLINDLYNRFTFYF-
---GDENVDKWD------------SNWITD-----KTID---------YASELSDIEKFNFYLN--ARKYFMEKPIIITT
ADQILMSFLNLER-------------YPIRYGMLMNSVIVFDEIQAYGMDM---RNLMYKLISEI--------
ANIKD--
---INGEYTCKLAITTATPPFEISVSEFKDYIAIDGIP---
FKLLYDKYWWSFHKKRDANVLISEIDKNSQIVDIIKQKV
NE--SKNEENS-RICIIVNTIKNAVDLYRLLEKKNAKDVIKENYEIALIHSSLINEH----KQKELDKI------
--NKS
KKLIAISTQVLEAGVDISFDCIIRLISPIPSLVQSAGRVNRDGKSRNAE---FIILLPP----------SDK--------
------------VGESGGKEESENSENNKKNKSNINF--YGPYDREEINKVIE---
LLRENKGNLIKKSEFSEEFIKK--
---EID-TSKFEYSQSKDYIDFVNNLVAYTIWSVSS---------LSSILP---QGFRYSLG----
KVEVFVEDESNKKK
DIEDKL-KNYIKLSK--------------------------ENDYSEYIRKYLQ-----LLREYQGRF------------
------IL--
-TYLDSKID---T------------IYIEKYKENFYPQHENSDK------------------------------------
----

>2830813125.Ga0394040_2807

------------------------MQDATL---SAIWAKSAKPDGKV-GESLVAHTANVVSRLYDFSK----------IY
----PQ-L-HIVT-DQPEFWQQAYWACVLHDFGKAASG--FQQQLRE------NS---
RSWGHRHEVLSLAFVQW-----
-ILPELDDE---W---------------LAWISAGIVSHHK----DLEVITRRYPFLDDDI--------------------
----------EAIAQLP-----------T--------DISIENINALAK---------------------FVEDDL
GSWNQRWQFPGTQIKSQLSADPSTDFFEHAEKRIRKNLTMVA-LLKRRL-------------------------------K
REDSKLRL-AGIAMRGIVTLSDHIGSAHVRPSAF-------------AFTS--ESQLL------------ELLPPQTRYK
HQELSAAA-----------KGNTILVAPTGSGKTESALLWLENQQKEK-----
TVGRLLYVLPYQASLNAMFLRLGSLF-
------PANIGLQHSRAAQSL--------FKSLLDKGYSPEQAAHN---------------ARKERSLAKLYKYGTRILT
PYQLLRAAFQLKGFEA------------LLT-DCTGAHLVLDEIHAYEAKRLGMILGFMEYLSKN--------
WQCKFFV
MSATLPTVLKNHLAESLDDCGELR--A-----AQA------LFQKFVRHQIHLIDSSIE----------------AADTI
TRIAFLAQSGQ-AVLVVCNTVKRARAVCDILKQTCQD-----
SITIDLLHSRFNSRDRLRKERQLLSQMGTKTRS--AGS
RGTILVATQVVEVSLDIDFDALFTETAPLEALIQRFGRVNRGRKHSFCPVYVLTEPKD------------
GQGVYNDDL-
-----IARVLELLPKLDGKFVDES------EISTWLDF---VYEGPLLT---KLEAELISAKR------EFQ--------
----------------AACIDALISFDSSEEL---------------------SDKFDKMFD----GSEVLPLQFLD---
-----------RYKALS------------------SRDPLIAAELLVPISYQQL-----SRIRRGGKLQCDG--------
---YLNIADVPYSEDFGLII--

>2549774010.M1622DRAFT_00976

----------------------------MT----CWA--------FYGMETFKQHSLGILNFFRDN-------FSYII--
PIISY--RTGI---DKETVRKSVEIGVSLHDIGKTSKH-------------------YDRSYFGHEFYGGYLVYK-----
-ILRECCD-----SE-----------LKPLVALAAMSHHQ----GMGGRTLNEMILKGNYT--------------------

---RI----------PPTYELREECR--NDILKILVEI
GVE---------------IKD--------FPQKV----TR-SDVKSWFQKL-------NIKW------------------
-------KNLYVIILGPLMVSDTVVA-----------NKNRGG--------N-QYNRI--IEEYE-----------KWIN
--------------------------VQ--

>650023445.Ssol98_010100000235

----------------------------MT----CWA--------FYGMETFKQHSLGILNFFRDN-------FSYII--
PIISY--RTGI---DKETVRKSVEIGVSLHDIGKTSKY-------------------YDMSYFGHEFYSGYLVYK-----
-ILRECCD-----SE-----------LKPLIALAAMSHHQ----GMEGRTLNEMILKGNYT-------------------
---RI----------PSFYELREECR--NDIVEILGEI
GVK---------------VKD--------FPQKV----TR-SDVKSWFQKL-------NIKW------------------
-------KNLYVIILGPLMISDTVVA-----------NKNRGG--------D-QYNKI--IEEYE-----------KWIN
--------------------------VK--

>2527531862.YNPFFACOM1_01266

--------MTLNSIY--LELCNERAW------------------------------KPRYFIEETLN-------------
---KLENL------LKENKRFVLIARLPTGYGKTTITNVLARAALKN----
NPYFCRVIHVLPMRSIADDVCLELQK---
--------DKM---ISNHVAVQHLLT--PGSPYFAKKCIVTT
LDTFLLNFFKIPTPELKKVFQYDTAHSEFPRAMIYLSIVIFDEFHLFAGLG-
DISNEGKALTSVIASMICLLKAKVPIII
MGAT----IPDVLIDKIREEISIVG----------------GEVESVNYVFDKDLEFDEK-----LRKKKKRIHIEEKDV
MEILNNIDSSK-SVLIVVNTVKKAKEIYNSITNKEEVG---------FLHGKIPEA-----IRAEIIKK-------
IKNS

KPRILIATQVIEAGVNISYDILITEPCPIDRLIQRAGRVCRFDEEEGD-----IYISKF----------
EKDYIYDENF-
-----VKTTLEVLNPNKGIERKES------NVLTF------EFANQAINEVYKI------------PIKELKEIDYDMKG
FLQELDALPILTSKYAK-------------------------------ELIIRYKGFTENFG----IVSCFNEKYLNKLY
AIPVSEKEGWELIKKHGKL-----IDANYRLIDFKAKLR-EVESLSIYL----------LDKGYQGIA------------
------
IPEEEYIEIIGFRPKIDIKTSASASKNISSSPSHHEISKPCAFIGQSLIKHTENTLEKALKMKYSFETMSK
RFE
ALGININAERLEKLIKAACILHDLGKAADEYQEDFYNSCECKG--
KVSFYLHEVASAYYAYKKLKSIEDYELDTKERQLI
TLAILFHIAGKNLYDLKDGVKEKKMKWTFNKYWKSFEHLLNKHRIKVDYKSSSINTEEAMNFLEE
IEEIIRKRESYYLKL
YNLIYSAICVGDNLDSYEFRRTDISESRKIFIEELKEVIANA
>2689041556.Ga0133342_11786

---------------------------------MSLD--------AFRGQTLERHVEVMLKAWDE------VKARYIPSI
-IRALK-AYNIEL-DEKKADRLMRALIILHDTGKGAQI--YQDHL-H------KG--
INLEGFRHELVSAYYARE-----
-----L--L--R--QVFDD----RTSFIG-A-LTVMLHHE----PILMGQISIL--------------DRDELSPE----
-------AALDKL-RKF------------------D--GVVPELEPFLKEMFE------------------------KHL
GIE---------------IK------------VP----EA--------------DLNAILRT-------IVELSVRARHI
PDSNRMRL-LVGALLIPLVLCDYRGA------------EEREG-------------ETPKFAKVL-----------EAEW
--------------------FVG---

>638169912.PAE0207

-------------------------------MTCHAW-K---GD-Q-CVEEYGRHIKRALTVWE-K-----LRPIYFPAV
-RRAI----------GV-DLDVVEYAIVVHDLGKLAKA--YQVG--------------RRGEYTHEVVSAYFAYK-----
-GLEPVV----------------GDEIAAVVAAAVLLHHE----PILT-SAY----ISGLGERY----------------
--------------------------------------LPIYAVRKMLEE--HDLSPACDPLA--------EAGDAVN--
-------------------------------KYP----ELKKELERWGNGGLT--PRDMLEVVKE-----IVVQTAVGDS
ARLHAMRA-KAAAVLYPLVVSDSVAAHVGRSLCG--CDERDKK--------G-TKIVDLALKGAE---------
PLDVEA
LRRELC--

>2755814153.Ga0213556_13822

--------------------------------MKELL-SH--YS-SDNRKKLKDHLLEVAKKSRELIL------------
-----DKDLNLSILSKNKLADISYLIGVLHDFGKTTTY--FQNYLIY-------G--ESSNLSHHGLISAIAGYC-
----
-LLKNQYNG---------------RLAMLG-YMVIKKHHGNLESPLESNERIYF----------------DLQ------
-KQ-LDNIKKNNF----EKLEAIY----------N---NLLADFDIK------------------------IKDVIEDI
EDF-----------------------------LF----DEEDIGEYFREEIIMNKFKDESK------------------
--AVELFL-INNLLYSVLIDSDKKNAARVNDDYFEGATKEKIE------------V-
KDYLDFCRENNPDKFNPEKLMNS
LRNDFFDDV-VNNSEISLNQYLYTLTAPTGIGKTFASFAFANKINSFH----
ENGRRVIYCLPYTSIIDQNYAEFEKII-
---KFNLKDKYDVKPTKYLLRHHYLSPLKIEE-NIKNKD------------
DSKESKDLDNYLANKLLLESWESGNIVTT
FVQLLESIIGNKNSYL------------KKFHNIVNSVVILDEVQNIPVKFYQVVGKVVNIFAEK--------
FNTYILL
MTATQPDILSGNEL------VN--------------------LIDERKYAEDPVFDRVNLKLLNELNP-FS----INDFI
DYFYSKFNSDN--CLIVCNTINSALNIYDKLKDKSN------DYKVYSLTTNLIPIDRKD-RINNIDEE------
-LKAG
EKVIVISTQLIEAGVDLSFKEVYRDFGPLDSIVQVAGRCNRNSEYENKGKVNIIKLKDE------
KDREFSKRIYDNKL-
-----LDICEEVLSENTS-----FLEMSKSYFAKVNDSFTRD-NKYLMNAIQNLN--
YSKKRDKEIPIKDFTIIE-----
---ER-AG----KEDIIICIDEGVEL
KV----RNLIELYEEIKETKD---NKKLNRLIAQKELINKELANYRVSVYRNQ------LEKYYSDYQIVNE-
F--KYLK
YVDYEDQKEYLYDENIGFLRDPKKPPASSLVF--

>2649207289.Ga0081668_11486

----------------------------MT----CWA--------FYGMETFKQHSLGILNFFRDN-------FSYII--
PIISY--RTGI---DKETVRKSVEIGVSLHDIGKTSKY------------------YDMSYFGHEFYSGYLVYK-----
-ILRECCD-----SE-----------LKPLIALAAMSHHQ----GMEGRTLNEMILKGNYT------------------
---RI----------PSFYELREECR--NDIVEILGEI
GVK---------------VKD--------FPQKV----TR-SDVKSWFQKL-------NIKW-----------------
-------KNLYVIILGPLMISDTVVA-----------NKNRGG--------D-QYNKI--IEEYE-----------KWIN
--------------------------VK--

>2512378622.Pogu_1144

------------------------------MSKCLAY-S---KE-G-CSESFVDHVERVVAVWD-M-----LRPYYLLPV
-QRAL----------GV-GGDPVLYAAVAHDFGKLALA--YQDQGK------------KRHFYRHELLSAYFAYK----
-
-CFEGPE----------------E--AKVAVSAAVWLHHE----PIIT-SAY----ITQLGESY----------------
--------------------------------------MPLYVVKRTLEFLQDELVVGCDYQE--------VSSHVRK--
-------------------------------WNS----CLAESLEQWAEKGLD--IAEVMRILKR-----VLVKATVGGI
ERAHVMRA-KIAAVLYPLIVADGVAAYVGRAVCG--RCGEEQR--------G-TKVIRAALCGAE---------
PLDKAA
LLK-LCR---

>2989478066.Ga0487045_15_208126_211140
-
MSSVPGPVQAVRVDLYAPVASFRDPMFPGVSRCLPVPPPSTVRGMLAAATGRASEPIQLGLAAFAE
GRGLDAETYHPIA
ADGSNPAVAGRVAPGKGGMTIRDRPFLIGVRLTIWIPAPEGDRIAQALRRPVWGLRLGRSQDLVHV
VSIREVTLHPTERA
RVGHALAPVGGHEAPLASTLRLAESISLDRGRTEYRSYQWCSEPA-GEHAVVGAYRD--
GDQAVWLSSPASAD-------
------------------------EHDAEL---SQVLAKSASGSGLGRPELLTEHSLAVRDATRTIAC----------RI
----GS-P-GVLA-SHPRFWSWVETAALLHDAGKVAEG--FQRQLRP------GG---
ESWGERHEVLSLAYVDL-----
-LTRELDVQ---E--------------RKMIAAGVAFHHR----PLTSHRSGDLRS--MYP-------------PV----
------AAWERKFGRDP-----------SPVLGRPRIQVPAARHRAWLG-------------------------WLAHQL
DT-------P-------VPAN-DRRLWELARDVF----AWL--SAEWIN------------------------------P
VD-DEDGL-VAVLLQGAVTLADHSGSAHVPLQTH-------------MPLP--RDFLGR-L--------------PTPYP
HQRQAADI-----------AEHLVLVAPTGSGKTEAGLSWASRQLDDL----
PGQPRLVWLLPYRASIDAARDRFIKHLD
PAPGQEHPDIGVLHATAARTL--------LARAIADDCTPGPVDAR-------------RARARAGA-
MRLFSQRVRVAT
PHQLLRAAIAGTRYSS------------VLL-EQANALMVLDELHAYDPATFGRICACMRLWQ-R--------
LGSRVAV
LSATLAKPMIELIRDTLG-SVEVR--R-----A-P------AGTAPARHRLTLDEDPID----------------SDASL
GRILGWLDEGQ-SVLVIANTVAMAQELFKRLD----------
HPGALLLHSRFRSRDRAEIERRILQRYPEREPGETAP-
RGGLVVATQAVEVSLCLDFDRGATELAPIEAIAQRAGRINRRGRHPDGLVEFRVHTEVI-----------
PNRPYDEGA-
-----LNASLTALMTAPGPELSEE------TIEIWLDI---AYSTEWGL---NWAVEAERHRD------EFS--------
----------------RDFLTFPDPFHDRSEF---------------------ADKLDAAFD----TVEVLRRADVD---
-----------EYRIE---A---------------EKDPLLAAGFLIPVRYTQL-----AALRAAGRAEFIK--------
RDLRLWVIDADYDPVLGLDLSLRPA--A-VPETVL---

>2588252189.EJ28DRAFT_01465

------------------------------LYSFKLL-SH-------PETLLVDHLEKVYNYGMEILK------------
-----ENKLFL---E---DRDFLKIILIGHDLGKATSY--FQDYIKK------IE--SHGFIKNHGFLSALFTYV-----
-ILQKKFDK--------------DK--ALKGFLIVKRHHGNIQNMYDELSMKKI-----------------EVE------
-SQ-IEILDKQLKTIDFKELNSIL----------EK--FNLPCVKS------------------------EEIKDIFIEL
F-------------------------------------DEMEAVIYY--------EDDMLL------------------
--NIEEYF-KLKYFYSILIYSDKFSVIFDKKR------EKNKE------------IKIEKLEEFIEKLPK----CKNIIN
DKRNEAKIN-
VESKIENLNKKIFTLTLPTGMGKTLNSLNFALKLREKLYKEKGIHYNIIYTFPFTSIIDQTYEIFQKIF-
---GN---------RTSDVLKHHYLSKVEY-------KD---------------SE-DYFETEKSKFLIETWDSKIVVII
FIKLLNCIFSNKNSEL------------LKFNKLANSIVILDEVQNIPYKYWKLIRHSFKVLSNL--------
LNTYFIL
MTATQPLIFKGDVE------L---------------------VENT-REYFKIFKRT--KLNIDLKE-RS----IDEFI
EEIESIIKRAN-KIMIVLNTVKSAQEVYKQIKDIT-------DKKVVFLSASVIPKDRKE-RILEIKKL--------
---
DKYILVSTQVVEAGVDIDNDVVIRDIGPWDSIVQCAGRCNRNNEKD-IGNVYIYKLKG-------
EKSVFANIVYGRFL-
-----ISKTEKILKDNSYIFESDYFDYSKYYFEEINKDKSDDYSNRIIEDINQLN--FSEVDNQ------
FKLIE-----
--NNSLY----IMPVFIEKDKEAAY
--------IWTKYEELFDI-----KDRFKRMNKFLE-IKEKFLSYVININIK-------------DFPFMRD-R--FYGK
I--PIENLDYYYSNEYGFITNNDQTMFF--

>2730674046.Ga0180975_10517
MTHDERIPIDAVRIELYAPVASFRDPMFPGVSRCLPVPPPSTVRGMLAAATGRPTESVVLGMSAHAT
GRGTDVETYHLVW
ADGGDPPIGGRGGKRKGSTTVRERPFLTGVHVTLWIPLPDGARIAAALRRPVWGLRLGRSQDLVH
VRSVTNVTLHPAEDA
HIGHALAPVGGHDAPQGTLLRLADHVSSDRLRTGFADFLWCAEPPEHSRPVTGAYRD--
GDQAVWLLAPSSVDPADTLEP
EGDDEPPDVGREPHQSGQEQS--RLDEAEL---
TQVLGKSKGASKLGRPELLTEHSETVRDAARAVAG----------RI
----AS-P-GVLA-ARPGFWRQVETAALLHDAGKVAEG--FQRQLRP------GG---
EPWGERHEVLSLAYVDL-----
-LTRHLPPD---D--------------RKMITAGVAFHHL----PLTSSSRRDLRE--MYP-------------PE----
------AAWKKKFGFDP-----------DAGPGRPRVQVPAARHTALLR-------------------------WLAEQL
GV-------TA------LPSE-TRRLWELARDAF----ERV--QADWSS------------------------------P
VP-PEDGL-VAVLLQGAVTLADRSGSAQVPLQEH-------------MPLP--RDFIRT-L--------------VTPYP
HQQATADT-----------VGNLILCAPTGSGKTEAGLAWASRQLDDM----
PGRPRLVWVLPYRASIDAARDRFIRDLQ
APPGAAEPDIGVLHATAARTL--------LTRATADDCPPGPAEAR-------------KARDQANA-
MRLFAQRVRVAT
PHQLLRAAIAGPSHSS------------LLL-EQANAVIALDELHAYDPATFGRLCAAMRLWR-D--------
LGSRVAV
LSATLAPPMIELIRDTLA-DVTVH--R-----A-P------PGTAPDRHRLVLDDQPID----------------APDSL
ARLRAWLAEGH-
SVLAVANTVATAQRVYAELAPAAHHACPDDPDAAILLHSRFRAKDRAEIERRILRRHPERSVGEPGH
R
GGGLVVATQVLEVSLCLDFDRGASELAPIEALAQRAGRVNRRGRHPDGPVEFRVHLPE-----------

TARPYKEDA-
-----LHAALSALRRTPEPVISEQ------TIDTWLRH---AYDTDWGR---EWTRQATRHRD------EFF--------
----------------DSFLTFTDPFHDRTEH---------------------AAGLTEAFD----TVEVLLERDAE---
-----------EYFDTLNGS---------------DGDPLLATGLLIPVSYAQL-----HRLAKDGRARLDRDH--ARRA
RHPPLWLIDAPYSTETGLDLSTANSSPV-PVETVL---

>641613515.Kcr_0434

------------------------MGRVIS----YYK--------PPVTEYLSEHINSCLNLLEKLES---SRI------
----G--RVGLSL-D-RNFVDEVRLSVVFHDFGKALYQSKISPRK------------SSVSFAGHEIFSTYFLFK----
-
-FRKSIIK-----GADFNEY---IK-L-KPALFAVTFHHH----PMDIGRRLERVQQIKLN-------------------
-----------------------------------T--SSLEDLQA-------ELSFLRDDALSKEER--DLLDSVVSEL
KGK---------------IENGSIRI-DDVKRIF----RE-EICKGFYEKYTS-GKEEDVIL------------------
-------KKLSYLTLVSLVSVDYISA-----------SERRGE--------G-TKFGD-VIDEFY-----------KLYL
--------------------------DI--

>2730768369.Ga0173049_11410

----------------------------MT----CWA--------FYGMETFKQHSLGILNFFRDN-------FSYII--
PIISY--RTGI---DKETVRKSVEIGVSLHDIGKTSKY-------------------YDMSYFGHEFYSGYLVYK-----
-ILRECCD-----SE-----------LKPLIALAAMSHHQ----GMEGRTLNEMILKGNYT-------------------
---RI----------PSFYELREECR--NDIVEILGEI
GVK---------------VKD--------FPQKV----TR-SDVKSWFQKL-------NIKW------------------
-------KNLYVIILGPLMISDTVVA-----------NKNRGG--------D-QYNKI--IEEYE-----------KWIN
--------------------------VK--

>2524568206.NAG2ff85r04_01613

-------------------------M----SEECYSY-I---ED-GNPIEKYFEHIDAALIVWR-E-----IRQYYYDAV
-TRVL----------ELEEFDPVEFAIVVHDLGKLTRE--YKTH-------------RGKFFRHELFSAYSCYK-----
-ILKKAH---------I------EDQKALPITLSVLLHHE----PILL-SAY----AGNLGENY---------------
--------------------------------------VAVSNIKKILHE--SNLSLACNPAS--------FGKYCL---
--------------------------------GD----RINEFIDKWKGIGQSELKDDAFKLLKE-----IILKSTVGPQ
KQLTKIRA-KAAALLYPLTVCDSIAAEMVRGDCK--NVQREKK--------G-TWVTERAKSGAE---------
QIKYED
LKKKVVKEL-------------GLKCDG--

>2831516021.Ga0365486_1337

------------------------------LYSFKLL-SH-------PETLLVDHLEKVYNYGMEILK------------
-----ENKLFL---E---DRDFLKIILIGHDLGKATSY--FQDYIKK------IE--SHGFIKNHGFLSALFTYV-----
-ILQKKFDK--------------DK--ALKGFLIVKRHHGNIQNMYDELSMKKI----------------EVE------
-SQ-IEILDKQLKTIDFKELNSIL----------EK--FNLPCVKS-----------------------EEIKDIFIEL
F-------------------------------------DEMEAVIYY--------EDDMLL-------------------
--NIEEYF-KLKYFYSILIYSDKFSVIFDKKR------EKNKE------------IKIEKLEEFIEKLPK----CKNIIN
DKRNEAKIN-
VESKIENLNKKIFTLTLPTGMGKTLNSLNFALKLREKLYKEKGIHYNIIYTFPFTSIIDQTYEIFQKIF-
---GN---------RTSDVLKHHYLSKVEY-------KD--------------SE-DYFETEKSKFLIETWDSKIVVII
FIKLLNCIFSNKNSEL------------LKFNKLANSIVILDEVQNIPYKYWKLIRHSFKVLSNL--------
LNTYFIL
MTATQPLIFKGDVE------L---------------------VENT-REYFKIFKRT--KLNIDLKE-RS----IDEFI
EEIESIIKRAN-KIMIVLNTVKSAQEVYKQIKDIT-------DKKVVFLSASVIPKDRKE-RILEIKKL--------
---
DKYILVSTQVVEAGVDIDNDVVIRDIGPWDSIVQCAGRCNRNNEKD-IGNVYIYKLKG-------
EKSVFANIVYGRFL-
-----ISKTEKILKDNSYIFESDYFDYSKYYFEEINKDKSDDYSNRIIEDINQLN--FSEVDNQ------
FKLIE-----
---NNSLY----IMPVFIEKDKEAAY
--------IWTKYEELFDI-----KDRFKRMNKFLE-IKEKFLSYVININIK------------DFPFMRD-R--FYGK
I--PIENLDYYYSNEYGFITNNDQTMFF--

>644808596.TGAM_1295

---------------------------------MTLL--------AFKGQPLEEHVKAMLQAWNE------VKGKYIPSI
-IRAMK-AYGVEL-SREKVDRLMRALIILHDSGKGARI--YQDYL-K------DG--
EKLGGFRHELVSAYYALK-----

-----I--L--S--QLFEE----RTAFVG-S-LVVMLHHE----PILMGQIANF--------------DRDSLSAE----
-------VTLDKL-RNF------------------D--GVVSELDEFLRKSFK------------------------KYL
GAD---------------VE------------VP----NA--------------ETGEVVRT-------VVELSVKARHL
PDAGKLRL-IVGALLIPLVLCDYKGA------------EDREG-------------ETPKFAEVL-----------EAEW
--------------------MGVV--

>646944875.Ssol_2280

----------------------------MT----CWA--------FYGMETFKQHSLGILNFFRDN-------FSYII--
PIISY--RTGI---DKETVRKSVEIGVSLHDIGKTSKY-------------------YDMSYFGHEFYSGYLVYK-----
-ILRECCD-----SE-----------LKPLIALAAMSHHQ----GMEGRTLNEMILKGNYT-------------------
---RI----------PSFYELREECR--NDIVEILGEI
GVK---------------VKD--------FPQKV----TR-SDVKSWFQKL-------NIKW------------------
-------KNLYVIILGPLMISDTVVA-----------NKNRGG--------D-QYNKI--IEEYE-----------KWIN
-------------------------VK---

>2511627530.TTX_1254

-------------------------------MSCCAY-F---RG-RDCLQTYEDHITQALEACE-R-----LRPYYGRWM
-EKAF----------GT--ADAAALAVEFHDLGKLAKA--YIAG--------------NRARYRHEVLGAYFALK-----
-TLQTE--------------------ARYYVAAAVALHHE----PMIL-AAY----AGELGERA----------------
------------------------------------IHVSTLRAMLKD--SDLSLGCTPNY--------S----------
-------------------------------YRP----EVAAALREWASRPPT--ADDVADAFQE-----LAVYLSGGAP
EEARVKRL-RVAGLLHVLTVCDNWGAR-----------GRPGE--------G-TFISRYMTVSEL---------GL----
----

>643828671.M1425_0862

----------------------------MT----CWA--------FYGMETFKQHSLGILNFFRDN-------FSYII--
PIISY--RTGI---DKETVRKSVEIGVSLHDIGKTSKH-------------------YDRSYFGHEFYGGYLVYK-----
-ILRECCD-----SE-----------LKPLVALAAMSHHQ----GMGGRTLNEMILKGNYT-------------------
---RI----------PPTYELREECR--NDILKILVEI
GVE---------------IKD--------FPQKV----TR-SDVKSWFQKL-------NIKW------------------
-------KNLYVIILGPLMVSDTVVA-----------NKNRGG--------N-QYNRI--IEEYE-----------KWIN
-------------------------VQ---

>2730742759.Ga0036909_11970

----------------------------MT----CWA--------FYGMETFKQHSLGILNFFRDN-------FSYII--
PIISY--RTGI---DKETVRKSVEIGVSLHDIGKTSKH-------------------YDRSYFGHEFYGGYLVYK-----
-ILRECCD-----SE-----------LKPLVALAAMSHHQ----GMGGRTLNEMILKGNYT-------------------
---RI----------PPTYELREECR--NDILKILVEI
GVE---------------IKD--------FPQKV----TR-SDVKSWFQKL-------NIKW------------------
-------KNLYVIILGPLMVSDTVVA-----------NKNRGG--------N-QYNRI--IEEYE-----------KWIN
-------------------------VQ---

>2264867995.A471O8DRAFT_00996

--------MTLNSIY--LELCNERAW------------------------------KPRYFIEETLN-------------
---KLENL------LKENKRFVLIARLPTGYGKTTITNVLARAALKN----
NPYFCRVIHVLPMRSIADDVCLELQK---
--------DKM---ISNHVAVQHLLT--PGSPYFAKKCIVTT
LDTFLLNFFKIPTPELKKVFQYDTAHSEFPRAMIYLSIVIFDEFHLFAGLG-
DISNEGKALTSVIASMICLLKAKVPIII
MGAT----IPDVLIDKIREEISIVG----------------GEVESVNYVFDKDLEFDEK-----LRKKKKRIHIEEKDV
MEILNNIDSSK-SVLIVVNTVKKAKEIYNSITNKEEVG---------FLHGKIPEA-----IRAEIIKK-------
IKNS
KPRILIATQVIEAGVNISYDILITEPCPIDRLIQRAGRVCRFDEEEGD-----IYISKF----------
EKDYIYDENF-
-----VKTTLEVLNPNKGIERKES------NVLTF------EFANQAINEVYKI------------PIKELKEIDYDMKG
FLQELDALPILTSKYAK-------------------------------ELIIRYKGFTENFG----IVSCFNEKYLNKLY
AIPVSEKEGWELIKKHGKL-----IDANYRLIDFKAKLR-EVESLSIYL----------LDKGYQGIA------------
------
IPEEEYIEIIGFRPKIDIKTSASASKNISSSPSHHEISKPCAFIGQSLIKHTENTLEKALKMKYSFETMSK
RFE
ALGININAERLEKLIKAACILHDLGKAADEYQEDFYNSCECKG--
KVSFYLHEVASAYYAYKKLKSIEDYELDTKERQLI
TLAILFHIAGKNLYDLKDGVKEKKMKWTFNKYWKSFEHLLNKHRIKVDYKSSSINTEEAMNFLEE
IEEIIRKRESYYLKL
YNLIYSAICVGDNLDSYEFRRTDISESRKIFIEELKEVIANA
>2620073532.Ga0070342_10331

------------------------------------M--------ASRHETLEEHLIACLQFLKS---------HFI-DL
-GYASHVAYSFGI-DEKEAVKALNATVIFHDYGKAANE--YQKKES-----------
KKLSFPKHEYFSATAAHK-----
-----S--I--K--ETVWR----DECL-----MAIGWHHM----AMKGPSLLDD--------------FTTWKKF-----
-------GAPEKA-TYP------------------A--EFVETFRKIAKD---------------------------NEL
GDM---------------LVN---------DPPL----EI--------------TLPEVRKTLE-------HISARLLSL
QTGHTFYR-RTLKLLRPLLIVDNLAA-----------AEKRDG-------------VEKIFVKDL-----------PDPA
E----------------IHQIRDVLRGLC---

>643842666.M1627_0927

----------------------------MT----CWA--------FYGMETFKQHSLGILNFFRDN-------FSYII--
PIISY--RTGI---DKETVRKSVEIGVSLHDIGKTSKH-------------------YDRSYFGHEFYGGYLVYK-----
-ILRECCD-----SE-----------LKPLVALAAMSHHQ----GMGGRTLNEMILKGNYT-------------------

---RI----------PPTYELREECR--NDILKILVEI
GVE---------------IKD--------FPQKV----TR-SDVKSWFQKL-------NIKW-----------------
-------KNLYVIILGPLMVSDTVVA-----------NKNRGG--------N-QYNRI--IEEYE-----------KWIN
--------------------------VQ--

>2682561660.Ga0128453_100555

--------------------------MNPL---QRVWAKSAPP-GTTRGELLTDHLDATLDALELLRS----------RV
----GR-I-A--G-LPERFWTWATLACLFHDTGKIPDG--FQRMVGNPR----PA---
QPWGLRHEIYSLGFVDH-----
-VLTHLSED---E--------------RRWVGLGVLTHHR----PLTGGAQSIKKTEELPP-------------HP----
------QGDSGHFW---TGRRA
DS--

>2730041368.Ga0180936_10651

------------------------MSEAYA----YLY--------GDRKQTLREHIYSCLEALDKIKS---SKI------
----W--NLVKKL-CGSSAERYIKIAVIFHDSGKILYQKNFKTKN----------DVKYLSFYGHEIYSTYFISE--
---
-FLNAKRR-----AIENVKQLDVLRLFKLAVIASVLYHHH----AMGVRDRIKRLSKISYE-------------------
-----------------------------------N--VFVKEFDKLIEDLLENPIFLSDILKNKSE-------------
---------------------GHLSP-NNVSNVV----SD-LNREIWIEXVKS-K-----KF------------------
-------RKTMLLFTNILLIADYMGS-----------QDRDGT--------K-TEFAK-VLDEFL-----------ELYS
-------------------------TSTHPAP---

>2999171050.Ga0490602_01_2047145_2047654

------------------------------MLPSQLY-SH-------PGKLLEEHLISTQKLIVHYLS------------
-----EMPDDL---AESALGITAKIVGLTHDLGKATDF--FQKHLKG------ER--VPKKLSRHSLFSALITYH-
----
-ILKEQFQN--------------NEMPMLG-YMTVLRHHGDLENPETEAYLEDE----EID----------LVK------
-KQ-IDNIDQEKWSILI----DNL----------YK--YGLPTIPTV----YC----------------LMINPVV---
----

>2682505067.Ga0128351_101735

------------------------------MNVCYSY-N---KE-GGKVERYENHIKSTVAAWS-V-----IKPYYEKVL
-DRVL----------GKLEFDPIAVALAIHDLGKLTNA--YEMS---------------PRNFRHEIFSGYAMYK-----
-ILGKR-----------------PEDVKMILSLAVTLHHE----NILMGGIY----VGELGERY----------------
--------------------------------------LTISNIRRILDTFKDKLKPNCDVKQ--------TFNELLKNF
LVR------------KGLIND----E-----FVN----DIMEFIDHWLNDGIK--IDDIIYSIKD-----MIAWASVGPL
EDMLIRRA-KVASVLHLITLADSIAANVLRS------GDMKDE--------G-NWIVERVSKGAE---------
PVDINE
IKDAINKVM-------------GNAP--

>2923402884.Ga0039428_01_777900_778748

-----------------------MS----RVACAYI-IRKATGEHECCETLEEHVQRSLEVWLGR-----LKKCYSSAV
-NRIIY-SQGSQT-LASVLGDPVAVAIVLHDIGKAVPA--YQKKRIM---------
RGRVQYRHELVSGALSLL-----
-AIEKAADM---AGIGRND----RGLYVFEVPLAVMMHHE----SISM-GVL----AGELGERF---------------
-------------------------------------VTLSVASLMLEDYYDGELWGLERLA--------RT---VK--
---------------------NMGA-----VGD----AVVDVLKILSGSPPS--RKEVLIALGR-----LKAYSLSGDT
LSRLSARA-RVAGILHPLVVADSIAANEGRKRCN------GDG--------G-TWIVKRALGRDS---------
QEPAEP
CLS--

>2621225351.Ga0061022_102028

-----------------------------LYSFKLL-SH-------PETLLVDHLENVYNCGIRILE------------
-----ENKLFL---E---DKEFLKIILIGHDLGKATLY--FQDYIKK------IE--SHGSIKNHGFLSALFTYS-----
-ILQKKFDK--------------DK--ALKGFLVVKRHHGNIQNMYDELSMKKI----------------EVE------
-SQ-LETLDKQLKTIDFRELNLIL----------EK--FNLPYVKS-----------------------KEIKDRFMEL
F------------------------------------DEMEAVIYY--------EDDMLL------------------
--NIEEYF-KLKYFYSILIYSDKFSVIFDKKR------EKNKE------------IKIEKLEEFIEKLPK----CKNIIN
DKRNEARIN-
VESKIENLNKKIFTLTLPTGMGKTLNSLNFALKLREKLYKEEGIYYNIIYTFPFTSIIDQTYEIFQKIF-
---GN---------RTSDVLKHHYLSKVEY-------KD--------------DE-DYFETEKSKFLIETWDSKIVVTT
FVKLLQCIFSNKNSEL------------LKFNKLANCIVILDEIQNIPYKYWKLIRHSFEVLSDI--------
LNTYFVL
MTATQPLIFKGDIE------L---------------------VENT-KEYFKIFKRT--KLNIDLKE-RS----IDEFI
DEIKPIIEKVN-KIMIVLNTVKSAQEVYKQIKNIA-------DKKVIFLSASVIPKDRKK-RILEIKKL--------
---
DKYILVSTQVVEAGVDIDNDVVIRDIGPWDSIVQCAGRCNRNNERD-IGDVYIYKLKG-------
EKSIFANIVYGRFL-
-----ISKTEKILKNNSHIFEHDFFDYSKDYFEEINKDKSDDYSNRIIEDINQLN--FNEVDNQ------
FKLIE-----
---NNSLY----IMPVFIEKDEEATY
--------IWTKYEELFDI-----KDRFERMNKFLE-IKEKFLSYVININIK------------DFSFVRD-R--FYGK
I--PIENLDYYYSNEYGFITNNDQTMFF--

>638201689.MJ0384

---------------------------------MEVL--------AFKNQSLIDHVNDMVKYWER------IKYRYLKTI
-KRALE-ALNIKL-DIEKVDEFMKILIKLHDIGKASKI--YQRAIIN------DQ--

EKLMGFRHELVSAYYTYH-----
-----I--L--L--KKFGDK---NLAFIG-A-LTVMLHHE----PIIMGQIRNL--------------KKKELTAE----
-------VVLDKL-KKF------------------D--GMIEDFEDLIKKLIG------------------------YSI
GDI---------------IK-------------N----DS--------------NKDDIIRF-------VIEMSVRARHT
PNSEKLRF-IVGTLLLPLVMCDYKGA------------ESREG-------------KAPKFAEVL-----------EVES
--------------------YVI--

>641668284.Tneu_1133

-------------------------------MSCCAY-F---RG-RDCLQTYEDHITQALEACE-R-----LRPYYGRWM
-EKAF----------GT--ADAAALAVEFHDLGKLARA--YTAG--------------NRARYRHEVLGAYFALR-----
-SVQTE--------------------ARYYVAAAVALHHE----PMIL-AAY----AGELGERA----------------
---------------------------------------IHVSTLRAMLKD--SDLSLGCTPNY--------S-------
-------------------------------YRP----EVAAALREWASRPPT--ADDVADAFQE-----LAVYLSGGAP
QEVRVKRL-RVAGLLHVLTVCDNWGAR-----------GRPGE--------G-TFISRYMAASEL---------GL----
----

>2771144415.Ga0254923_10253

------------------------MS----NTMCISF-S---RGNCRRSEPYEVHIKASLIAWS-V-----LKPFYYGAI
-NRIL----------GKLPFDPVSIALTLHDLGKLTKS--YEMN---------------RREYRHELFSAYATYE-----
-ILENMSKN---G-VPI------PNDAVRLISLAVMLHHE----PIIM-GMY----VGRYGEEY----------------
--------------------------------------LTISNLRRVIELNVSNLSPNCDVEN--------AMNNLIN-Y
LVT------------ENFITKDSGNS-----FRT----AVNDFIKKWRSS-LN--DEEIFNVLKR-----VIVYSTVGAP
NKLLTIRA-KAAALLHLVVVSDSIAANIVRSELE--CSDERDG--------G-IWIVERIRSGAE---------
PVSYKK
IREEIEKRV-------------LLNIG--

>2587808271.EJ24DRAFT_01427

------------------------------LYSFKLL-SH-------PETLLVDHLENVYNCGIGILE------------
-----ENRLFL---E---DKEFLKIILIGHDLGKATSY--FQDYIKK------IE--SHGSIKNHGFLSALFTYS-----
-ILQKKFDK--------------DK--ALKGFLIVKRHHGNIQNMYDELSMKKI-----------------EVE------
-SQ-LETLDKQLKTIDFRELNLIL----------EK--FNLPYVKS-----------------------EEIKDRFIEL
F-------------------------------------DEMEAVIYY--------EDDMLL-------------------
--NIEEYF-KLKYFYSILIYSDKFSVIFDKKR------EKSKE------------IKIEKLEEFIEKLPK----CKNIIN
DKRNEARIN-
VESKIENLNKKIFTLTLPTGMGKTLNSLNFALKLREKLYKEEGIYYNIIYTFPFTSIIDQTYEIFQKIF-
---GN---------RTSDVLKHHYLSKVEY-------KD---------------DE-DYFETEKSKFLIETWDSKIVVTT
FVKLLQCIFSNKNSEL------------LKFNKLANCIVILDEVQNIPYKYWKLIRQSFEVLSDV--------
LNTYFIL
MTATQPLIFKGDVE------L----------------------VENT-KEYFKIFKRT--KLNIDLKE-RS----IDEFI
DEIEPIIKRAN-KIMIVLNTVKSAQEVYKQIKSIA-------DKKVIFLSASVIPKDRKE-RILEIKKL---------
--
DKYILVSTQVVEAGVDIDNDVVIRDIGPWDSIVQCAGRCNRNNERD-IGNVYIYKLKG-------
ERNIFANIVYGRFL-
-----ISKTEKILKDNSHIFEHDFFDYSKYYFEEINKDKSDDYSNRIIEDINQLN--FNEVNNQ------
FKLIE-----
---NNSLY----IMPVFIEKDEEATY
--------LWTKYEELFDI-----KDRFERMNKFLE-IKEKFLSYVININIK-------------DFPFVRD-R--FYGK
I--PIENLDYYYSDEYGFITDNDQTMFF--

>2682526968.Ga0128353_10275

--VEDTEPFIKGMMK------------------------EEF
GVA---------------PE------------VP----SP--------------TREDVLRE-------VIRLSVLARHR
PDSSRLRM-VVGALLIPLVLCDYKGG-----------AEKREG-------------EAPKFAEVL-----------GVEM
--------------------TL--

>2549777118.M1630DRAFT_00957

----------------------------MT----CWA--------FYGMETFKQHSLGILNFFRDN-------FSYII--
PIISY--RTGI---DKETVRKSVEIGVSLHDIGKTSKH-------------------YDRSYFGHEFYGGYLVYK-----
-ILRECCD-----SE-----------LKPLVALAAMSHHQ----GMGGRTLNEMILKGNYT------------------
---RI----------PPTYELREECR--NDILKILVEI
GVE---------------IKD--------FPQKV----TR-SDVKSWFQKL-------NIKW------------------
-------KNLYVIILGPLMVSDTVVA-----------NKNRGG--------N-QYNRI--IEEYE-----------KWIN
--------------------------VQ--

>646418632.Tcur_0935
-
MPDERRPIEAVRVQLYAPVASFRDPMFPGVTRCLPVPPPSTLRGMLAAATGRPAEPVVLGVCAYAE
GRGVDTETYHPIA
ADGSNPAIGGRVRPGKGGMTIRERPFLTGVHITLWVPMPDGERIATALRRPTWGLRLGRSQDLVHV
RSITRVELVPTDRA
RIGHALAPRGGHQAPQANLLRLADHVSTDRLRTDYGDYLWCAEPPTDVRPVAGAYRD--
GDQAVWLQAPPAPET------
-------------------------GDDGEL---AGVFGKSKAASKLGRPELLIEHSVTVRSAAQAVAE----------RI
----GS-P-GILA-DRPGFWTQVEIAALLHDAGKVAEG--FQNQLRP------GG---
EPWGERHEVLSLAYADL-----
-LTRHLPES---D--------------RTMIAVGVAFHHR----PLISASGRDLRE--MYP-------------PE----
------AAWERKFGRDP-----------DAPPTRPRVQVTPARHAALLR-------------------------WLAAQL
GI-------PP------PEPD-GRRLWELARDAF----ARL--HAAWRG------------------------------R
VP-PEDGL-VAVLLQGAVTLADRSGSAHVPLQTH-------------MPLP--RRYITT-L--------------PAPYP
HQRAAAET-----------TGHLVLCAPTGSGKTEAGLAWASRQLDDM----
PGRPRLLWVLPYRASIDAARDRFIKSLL
PAPGQKKPDIGVLHATAARTL--------LARATTDDCPPGPRQAR-------------KARDQAAA-
MRLFAQRIRVAT
PYQLLRGAIAGPSHSS------------VLL-EQANALIALDELHAYDPATFGRLCAAMRLWQ-M--------
LGSRVVV
LSATLAPPMIDLLKDTLD-QVTVH--R-----A-P------PGTAPDRHRLVLDDQPIT----------------APQSL
DRIRRWLTEGH-
SVLVVANTVATAQRLYRELAGHARAVLPDDPDAAILLHSRFRAEDRAAIERRISTRHPERAVGENTR
-
RGGLVVATQVLEVSLCLDFDRGVSELAPIEAVAQRAGRVNRRGRHPDGVVEFRVHMPE-----------
SALPYEEGA-
-----LDAALSALRNTPSPVISEQ------TVETWLRH---AYDTEWGR---QWASQAERYRK------EFF--------
----------------ESFLTFCDPFHDRTEH--------------------ATGLDEAFD----SIEVLLESDAE---
-----------EYSLRSSGP---------------HGDPLQAAGLLIPLRKAQI-----HRLHRNGRAHLDRER--ARRE
RRPPLWLINAPYSEETGLDLSAEATSPR-QPETVL--

>2265120379.A471L13DRAFT_00465

--------MTLNSIY--LELCNERAW-----------------------------KPRYFIEETLN-------------
---KLENL------LKENKRFVLIARLPTGYGKTTITNVLARAALKN----
NPYFCRVIHVLPMRSIADDVCLELQK---
--------DKM--------------------------------------ISNHVAVQHLLT--PGSPYFAKKCIVTT
LDTFLLNFFKIPTPELKKVFQYDTAHSEFPRAMIYLSIVIFDEFHLFAGLG-
DISNEGKALTSVIASMICLLKAKVPIII
MGAT----IPDVLIDKIREEISIVG----------------GEVESVNYVFGKDLEFDEK-----LRKKKKRIHIEEKDV
MEILNNIDSSK-SVLIVVNTVKKAKEIYNSITNKEEVG---------FLHGKIPEA-----IRAEIIKK-------
IKNS
KPRILIATQVIEAGVNISYDILITEPCPIDRLIQRAGRVCRFDEEEGD-----IYISKF----------
EKDYIYDENF-
-----VKTTLEVLNPNKGIERKES------NVLTF------EFANQAINEVYKI------------PIKELKEIDYDMKG
FLQELDALPILTSKYAK------------------------------ELIIRYKGFTENFG----IVSCFNEKYLNKLY
AIPVSEKEGWELIKKHGKL-----IDANYRLIDFKAKLR-EVESLSIYL----------LDKGYQGIA------------
------
IPEEEYIEIIGFRPKIDIKTSASASKNISSSPSHHEISKPCAFIGQSLIKHTENTLEKALKMKYSFETMSK
RFE
ALGININAERLEKLIKAACILHDLGKAADEYQEDFYNSCECKG--
KVSFYLHEVASAYYAYKKLKSIEDYELDTKERQLI
TLAILFHIAGKNLYDLKDGVKEKKMKWTFNKYWKSFEHLLNKHRIKVDYKSSSINTEEAMNFLEE
IEEIIRKRESYYLKL
YNLIYSAICVGDNLDSYEFRRTDISESRKIFIEELKEVIANA
>2904614814.Ga0450666_06_78715_81162

---------------------------------MQLLGKRAEINGSLRFQTLAGHTKDVLWVLKAIME---QP--SFSLF
----CR-R--WGL-NEAETKEILAAIAALHDIGKATET--FQQAIRE------GR---
HLHDVPHALVALPVAHEVWNCL
SLPRLLSEN---Q-------------LPIVELLAIVSHHA----LLYEDLYQ---------------------VA----
------IRHNEKLRFCP-----------EA------------------H-------------------------WVLSDI
FAWAKQQGLLRTSALPNLPF-SEWSGWELKKCAQ-----ALSNLRELI-RKIK-----------------------GQC
VETERLKA-LYAFPLAYLKFADQWASRKFSEQAAQ---LTTGIVDELLPDPPNWWLPDDAKQRVW---
QKLTENGKQPYR
FQEQLART----------EAERVVLLAPCGRGKTEGALLWFLNQRTLG-----
KCDRLVIAMPTQVTSNAMRERLANLF-
---G--DQCVGLYHGRSSLEHRELVRLQLAKEGEGDDLNPDIEREL---------------
AHSENFWSEVFAKPVTVTT
ADHLLFTFVHGFRQAD------------FALGCLQTAAIVFDEVHCYDRKMLSELRELFKLLR-K--------
MGIPHLV
MSGTLPEFLVKEGHLTD--YEPIV--D-----D--------EGLERRPFILRKREQPLFNKTDSET--
AGSKWQPEESVV
EEVLEGFRKGL-RQFVIVNTVRKAQAFYRALKRHIVE-----
PERLWCLHSRFCYDHRRKKEQRLMELL-------RADF
RPLILVATQVIEVSLDISCDRMFTELAPIDALGQRAGRLHRGASEPDGHELLVFRIEDP------------
QPYFLPHTK

QPLPELERTWEALLDN--FAVSYG------WLRERCDE---VYADARLG---MAQL-----PN------L-F--------
----------------EACTLFGLNYDEVRFSEEE-------G--------KAYCPRDIVMP----TVDVIPQSILD---
-----------QLGDKG-----------------------CHPLYLAPVPVWWI-----GKSNREGLGLFYS-H----QV
GKHEWLICRIPYDQDLGFDEEQIGVPPKGVIVD---

>2265121156.A471L14DRAFT_00233

--L----------LDKGYQGIA------------

IPEEEYIEIIGFRPKIDIKTSASASKNISSSPSHHEISKPCAFIGQSLIKHTENTLEKALKMKYSFETMSK
RFE
ALGININAERLEKLIKAACILHDLGKAADEYQEDFYNSCECKG--
KVSFYLHEVASAYYAYKKLKSIEDYELDTKERQLI
TLAILFHIAGKNLYDLKDGVKEKKMKWTFNKYWKSFEHLLNKHRIKVDYKSSSINTEEAMNFLEE
IEEIIRKRESYYLKL
YNLIYSAICVGDNLDSYEFRRTDISESRKIFIEELKEVIANA
>2522052361.Py04_0801

---MEAHIKEGLKFIEKIY----IKRNYGKFL
-------SQVLGL-KKENAEDLLRKAYAIHDVGKCLEE--FQKTKK--------------GFQFHEFYSALIAKE-----
-----V--L--R--QFGKA----GS-I---ATVAIFLHHHDWIR--------E----------------KPPRKPE----
----------------N------------------L--KIHPDCAEIIKELAE------------------------IQV
PMN---------------VP------WIAP-----------NEFKDWMAD------------------------------
--IFASNIRAVYALLLPISLADNYSA-----------IRNREG-------------EPTMLGREIM----------EVIS
--------------------THEEVKACLR---------YSQ--------------------------------------
----

>2771197259.Ga0254949_1178

------------------------MDLKLS----KPY--------AYRDQSLVDHIMGCLGKLKDFLR---ANPNYVRVV
HTRLL--SAGINVAKQEDVRRMLKLGVVAHDLGKAYKY--YQDRV----------
ERYGGGFEGHEILSAVSCYK-----
-MIGGLRD-----QN----P------LKVLLLMAVLNHHQ----ALRE--SIPELLM-----------------------
---DKASPLIKK-------IRDVARSGLCASVYNLEPVLGEV
GLS---------------IEEAFARDYPEFESIF----DE--IRGLLAFLLRK-NFDENKRW------------------
-------LKLYSLLMFPMVLVDNLDA-----------CEKRGG--------S-AGDRL-IIRELR-----------RVIE
--------------------------DE--

>650831767.PNA2_1817

------------------------MSDNVL---KECE--------AFPGQTLKEHVEEMLSAWDM------VKSKYILSI
-IRVMR-AVGIEL-KEKDADRFMRALIILHDIGKCSRI--YQNYL-E------GK--
EELGGFRHELVSAYYTYE-----
-----I--L--K--RTFESE---TLAFIG-A-LVVMMHHE----PILMGQILSL--------------QKENLSPE----
-------VVIDKL-RNF------------------D--GIVDGTESLLKELME------------------------KYL
NVD---------------LD------------VP----KP--------------TKEDILDR-------ITSLSVLARHG
SEADKLRL-VVGALLIPLVLCDYKGA------------ESREG-------------ETPKFAEIL-----------EVEM
--------------------IV--

>2648418494.Ga0097871_10223

--------MTLNSIY--LELCNERAW-------------------------------KPRYFIEETLN-------------
---KLENL------LKENKRFVLIARLPTGYGKTTITNVLARAALKN----
NPYFCRVIHVLPMRSIADDVCLELQK---
--------DKM--ISNHVAVQHLLT--PGSPYFAKKCIVTT
LDTFLLNFFKIPTPELKKVFQYDTAHSEFPRAMIYLSIVIFDEFHLFAGLG-
DISNEGKALTSVIASMICLLKAKVPIII
MGAT----IPDVLIDKIREEISIVG----------------GEVESVNYVFDKDLEFDEK-----LRKKKKRIHIEEKDV
MEILNNIDSSK-SVLIVVNTVKKAKEIYNSITNKEEVG---------FLHGKIPEA-----IRAEIIKK-------
IKNS
KPRILIATQVIEAGVNISYDILITEPCPIDRLIQRAGRVCRFDEEEGD-----IYISKF----------
EKDYIYDENF-
-----VKTTLEVLNPNKGIERKES------NVLTF------EFANQAINEVYKI------------PIKELKEIDYDMKG
FLQELDALPILTSKYAK-------------------------------ELIIRYKGFTENFG----IVSCFNEKYLNKLY
AIPVSEKEGWELIKKHGKL-----IDANYRLIDFKAKLR-EVESLSIYL----------LDKGYQGIA------------
------
IPEEEYIEIIGFRPKIDIKTSASASKNISSSPSHHEISKPCAFIGQSLIKHTENTLEKALKMKYSFETMSK
RFE
ALGININAERLEKLIKAACILHDLGKAADEYQEDFYNSCECKG--
KVSFYLHEVASAYYAYKKLKSIEDYELDTKERQLI
TLAILFHIAGKNLYDLKDGVKEKKMKWTFNKYWKSFEHLLNKHRIKVDYKSSSINTEEAMNFLEE
IEEIIRKRESYYLKL
YNLIYSAICVGDNLDSYEFRRTDISESRKIFIEELKEVIANA
>2637142231.Ga0081679_11484

---------------------------MT----CWA--------FYGMETFKQHSLGILNFFRDN-------FSYII--
PIISY--RTGI---DKETVRKSVEIGVSLHDIGKTSKY-------------------YDMSYFGHEFYSGYLVYK-----
-ILRECCD-----SE-----------LKPLIALAAMSHHQ----GMEGRTLNEMILKGNYT-------------------
---RI----------PSFYELREECR--NDIVEILGEI
GVK---------------VKD--------FPQKV----TR-SDVKSWFQKL-------NIKW------------------
-------KNLYVIILGPLMISDTVVA-----------NKNRGG--------D-QYNKI--IEEYE-----------KWIN
--------------------------VK--

>2829975503.Ga0373264_988

--------------------------MDF---SVYKAKSDDL-TFALCMTLEEHTRRVVRAGIELVN----------RL
----PF-T---ED-ERNKWLKKAIRCAVWHDIGKLHKS--FQANLKK------GT---TVVSIRHELISLWFCEQ-
----
-FLNLP-ID---E------------------LFAIATHHK----GIVSDGFKKLSL---YE-------------IG----
------ADLACHLSDDP-----------ELV----------------K-------------------------NAEEIV
KAWGTLFNDVS-PITNNVSP---KDAYSDEI-------QFL---------LMN------------------------DKQ
QSQPTDRL-QLARMRGLLMAADHIGSAKTESDLPV---WKR------LELT--DFYPTT-LNN------
KNKIVRVPFRP

FQERMQRY-----------IGDVILHAPTGSGKTEAALSWVYANQT-------
ENARLFYLLPYTASINAMVRRLKGVY-
---G--KERVTALHSKALDFF--------YEEAINETNNFQQSEEI---------------ARNKRSASRELFYPVKVAT
LHQVLKHALHGKGWDM------------SLF-DYQNALFIIDEFHTYDAHLTGMMLATLKWLKRF--------
CNAKILL
MSATIPKFLLDRIVNELFDGDTSRILR-----PNPGNPADADILKRIRHKLICQPK-KT----------------
VIDST
EQITQILEEGKKTVLIIVNNVSTCQKLSQTFD----------KFNPKLLHGGFHRKDRKEIEKSITAD--------
DRKE
RPRLLIATQAVEVSLDIDYDVAFIENAPIDALIQRFGRVNRAGKRKKPSEIYLFELSI----------
GNTKRIYDQAI-
-----VDETWRVLLKLDGQELSEQ------HLVDACDE---VYEKGYTE---EQERNFQI--G------FNH--------
----------------ERIVNFESTFHAG-DW--------------------ADWVEDVIDKTNLKIDILCENLRS---
-----------EFEAK------------R-----KEGKYIEASQLLVQAYP-----WE-----------CPKEHQIKLED
KQNTLVATNLEYTKGVGYQRIERSPDFHFL--

>2682544996.Ga0128346_11021

----------------------------MT----CWA--------FFGQETFKDHALGTLDCFRRN-------FAYIN--
PILSH--RTGV---EISTVKKNIEIAVAFHDVGKASKA-------------------YTTSYYGHEFYSGYIVSS-----
-MIRGCCN-----SE-----------LRDIAALSSMSHHQ----AMVERGLELI-RSEEY--------------------
--KK----------LHNFEFNEECL--DDINEVAKEI
GIS---------------VNL--------DRKLI----TP-EDVASWFMKI-------NNEK------------------
-------FYLYPIILGPLMVCDTYTA-----------NAHREG--------G-TGTY----------------------
----

>2828765395.Ga0392504_448

---------------------------------MTLL--------AFKGQPLEEHVKGMLQAWDE------VKGKYIPSI
-IRAMK-AYGVKL-GWDDADRLMRALIILHDSGKGARI--YQDYL-K------SG--
EKLRGFRHELVSAYYALK-----
-----I--L--S--QLFEE----RTAFVG-S-LVVMLHHE----PILMGQIANL--------------DRDSLSAE----
-------VALDKL-RNF------------------D--GVVSELDEFLRKSFK------------------------KYL
DVD---------------VE------------VP----NA--------------ETGEVVRT-------VVELSVKARHL
PDAGKLRL-IVGALLIPLVLCDYKGA------------EDREG-------------ETPKFAEVL-----------EVEW
--------------------MGVEGDGKY---

>2774029930.Ga0263258_11175

---------------------------------MK---YEIY--------AYKGQSLKEHVESMLKAWEI------AKKKYISSI
-ARVMS-SENIKI-SHSEVDRFMKSLIILHDTGKGAKI--YQENI-E------TG---
EFRGFRHELLSAFYTKE-----
-----I--L--E--QIFDI----KKAFIG-A-LVVMIHHE----PILMGQIENI--------------QKEELSPE----
-------VIVDKL-RNF------------------N--GVVPALKDFIVDSFK------------------------DFL
NVP---------------VN------------VP----DV--------------DLDALMQT-------VMELSVRARWL
PNSDRLRV-VVGCILLPLVLCDYKGA------------EERGG-------------EAPEFSKVL-----------ELEW
--------------------IGLR---

>2752495578.Ga0213453_10323

--------MTLNSIY--LELCNERAW------------------------------KPRYFIEETLN-------------
---KLENL------LKENKRFVLIARLPTGYGKTTITNVLARAALKN----
NPYFCRVIHVLPMRSIADDVCLELQK---
--------DKM---ISNHVAVQHLLT--PGSPYFAKKCIVTT
LDTFLLNFFKIPTPELKKVFQYDTAHSEFPRAMIYLSIVIFDEFHLFAGLG-
DISNEGKALTSVIASMICLLKAKVPIII
MGAT----IPDVLIDKIREEISIVG----------------GEVESVNYVFDKDLEFDEK-----LRKKKKRIHIEEKDV
MEILNNIDSSK-SVLIVVNTVKKAKEIYNSITNKEEVG---------FLHGKIPEA-----IRAEIIKK-------
IKNS
KPRILIATQVIEAGVNISYDILITEPCPIDRLIQRAGRVCRFDEEEGD-----IYISKF----------
EKDYIYDENF-
-----VKTTLEVLNPNKGIERKES------NVLTF------EFANQAINEVYKI------------PIKELKEIDYDMKG
FLQELDALPILTSKYAK------------------------------ELIIRYKGFTENFG----IVSCFNEKYLNKLY
AIPVSEKEGWELIKKHGKL-----IDANYRLIDFKAKLR-EVESLSIYL----------LDKGYQGIA------------
------
IPEEEYIEIIGFRPKIDIKTSASASKNISSSPSHHEISKPCAFIGQSLIKHTENTLEKALKMKYSFETMSK

RFE
ALGININAERLEKLIKAACILHDLGKAADEYQEDFYNSCECKG--
KVSFYLHEVASAYYAYKKLKSIEDYELDTKERQLI
TLAILFHIAGKNLYDLKDGVKEKKMKWTFNKYWKSFEHLLNKHRIKVDYKSSSINTEEAMNFLEE
IEEIIRKRESYYLKL
YNLIYSAICVGDNLDSYEFRRTDISESRKIFIEELKEVIANA
>2729863776.Ga0181037_105274
-
MTRPSAPIEAVRIELYAPAASFRDPMFPGVTRCLPVPPPSTVRGMLAAATGRAGEPVPVGMCAHAD
GRGTDAETYHPIA
ADGSNPAVAGRVREGKGGMTIRDRPFLVGVHVTLWIPAPHADRVAAALRCPVWGLRLGRSQDLVH
LRSVTPVTLYPADEA
VVGHALAPLGGHVAVQATSVRLADTISTDRLRTSFTDYLWCAEPA-
GRQPVSGAYRDPTDDQAVWLHTAGTTD-------
-----------------------AGDPEL---AQVWAKSAERSPTGRPELLTEHSTLVHDAAGTVAD----------RI
----GS-P-GLLA-HRPWFWSCVKTAALLHDTGKVGEG--FQLQVRP------GG---
EYWGERHEVLSLAYVDL-----
-LTRDLPEQ---D--------------RAMIKAGVVFHHR----PLVSTSGKELRL--DYP-------------PD----
------ADWQRKFGRDP-----------DPPPGRPRIQVPAKRHRALLR-------------------------WLTAHS
GL-------VA------A-EE-TRKLWELARDAF----AQV--SADWFH------------------------------P
VP-DEDGL-VAVLLQGAVTLADHAGSAHVPLQTH-------------MPLP--RSYLST-L--------------AAPYP
HQKAAADT-----------AGHLVLCSPTGSGKTEAALAWASSQLETM----
PGRPRLVWVLPYRASIDAARERFRDNLT
PPPGAKKPDIGVLHATAAQTL--------LSDAVQDDCASEKTPDG---------
AAADQARSRAHAMRTLFVQRVRVAT
PHQLLRAAIAGPRYSS------------VLL-EQANALMVLDELHAYDPVTFGRICAAMRLWE-R--------
LGSRIAV
LSATLAPPMLDLVAETLEQDVTVH--R-----A-P------PGTAPDRHRLALDELPLT---------------HPDSL
ARVRCWLADGH-
SVLLVANTVATAQQLYTDLAPHARTVRPEDPDAAVLLHSRFRGRDRAAIERRIKRRHPERKPGESG
R-
RGGLVVATQALEVSLCLDFDRGASELAPVEALAQRAGRVNRRGHHPEGPVEFRVHRTD------------
EHRPYDPGA-
-----ITAAWEALQTAPGPVISEQ------TIEDWLRL---AYDTDWGR---TWHAEARRARD------EFT--------
----------------DTFLTFTDPFHDRSEF---------------------AEGLDKSFD----TVEVLHHDDIA---
-----------DYRRQ---R---------------AEHPLLASGLLIPVRYTQL-----KVLESAGHADFDS--------
-KLRLWTTKAPYNTETGLDLTAGRAPAA-PKDTIL---

>2549780190.M1646DRAFT_01038

----------------------------MT----CWA--------FYGMETFKQHSLGILNFFRDN-------FSYII--
PIISY--RTGI---DKETVRKSVEIGVSLHDIGKTSKH-------------------YDRSYFGHEFYGGYLVYK-----
-ILRECCD-----SE-----------LKPLVALAAMSHHQ----GMGGRTLNEMILKGNYT-------------------
---RI----------PPTYELREECR--NDILKILVEI
GVE---------------IKD--------FPQKV----TR-SDVKSWFQKL-------NIKW------------------
-------KNLYVIILGPLMVSDTVVA-----------NKNRGG--------N-QYNRI--IEEYE-----------KWIN
--------------------------VQ--

>2264896305.A3IWDRAFT_00522

--KKALRMKYSFETMSRRFK
ALGLNINAEKIEKLITVACVLHDLGKVADEYQEFFDERCECKS--
ESSFYLHEIPSAYYVYKQLKDIEDYKLDFNAKRVL
TLAILFHIAGRDIDDLMDSIEN--RKWNFKKYWKDYEAFISKYKIKLDYKS-
SINTQEALDFLKEIKNIMTNKSYKYLKL
YNLIYGAICIGDNLDSYENRRTSISESRKTFIRELYEAIGNE
>2684057382.Ga0124707_10678

---------------------------------MSLL--------AFQGQTLRKHVNAMLNAWES------VKGKYIPSI
-IRAMK-AWGVEL-SREDADRLMKALIILHDSGKGAEL--YQDYL-E------GK--
ARLSGFRHELVSAYYALK-----
-----I--L--P--QIFDE----KTAFVG-S-LVVMLHHE----PILMGQVANL--------------DRDSLSAE----
-------VALDRL-KNF------------------D--GVVPELNEFLENSFR------------------------EHL
GVE---------------IT------------VL----SA--------------SPDEVVRA-------VVELSVRARHL
PDAGRLRL-IVGALLIPLVLCDYKGA------------EEREG-------------ETPKFAEVL-----------EAEW
--------------------LGVV--

>2923984953.Ga0477847_59_25057_27366

--------------------------------MTLLAKSKDSKSNISAQTLTEHINACLLVWERIKF----------AF
----PE-VATVSG-IGGRFWDILRQCIIFHDLGKAHIE--FQKLLHE-K----PN---
QWKNQRHELFSIPFVDA-----
-LTSED-QE---I--------------LSLIRFVILGHHK----DYETLRNAISFYEI---------------------
----------ESFGGLS-----------GL-------DETEN----FSA------------------------AFENHV
DI-------DG------VKQ--VLNEFEITIETV----MPKS-VSKWIHQYIS------------------------RPC
RLDNDKSL-LLFLLFGGLKWCDHLGSAMVSNIPVL------------HMSD--FSFLDS-KRA------
RLCQQGYDFYA
HQLVCSQT-----------VGNVVLTAPTGSGKTEAALLWARKQMEQS----
GQTGRVYYILPFTASINAMYERLSEYF-
---GKEKEMVGMLHGKLSDYL--------NNYFEDAQYDLGTKKEE--------------
IQQIRSKFRSLTTSVKVVT
PFQLLKHIFGLKGYEQ------------GFF-EMAGSYLIFDEIHAYSPDVFAQIKVLLEFAQKY--------
LNSHVLI
MTATMPKFLQAELEQSIKGPKLVQ--A-----DID------LYKKFVRHRVVLQEGLLA------------------DST
SNIIDCLQRDM-KVLVVCNTVLSAQNVFRDLCKYVG------KDEAVLLHSSFTGKDRSLKEQMLKL--
----------N
DIRLLVGTQAIEVSLDIDYDIIFTEPAPIDALIQRFGRVNRKREKEICDCIVFREANNS-----------
DKFIYPTEI-
-VYK-TIQALEQIIHEKGGVVDEA------LLQNSIDF---VYD-QWGE---DGKTKFDK-QY------FYL--------
----------------NEAIKLLAPMQKNKVQ--------------------EEDFYKQFD----GVKILPQSLVG---
-----------EYTTCMNQF-----------------DYISAESYKVSISSRRFKYWIMNDFVRLGSVHTSKKD----KI
IENRYYMTNKTYSSELGLLMDEEDY---WVESDFL---

>2913344159.Ga0451167_03_44250_44966

--------------------------------MSVL--------AFQGQTLGKHVEAMLKAWES------VKEKYIPSI
-IRAMR-AYGVEL-SRGDADRLMKALIILHDSGKETRL--YQDYL-A------GK--
AKLSGFRHELVSAYYTLK-----
-----I--L--P--QLFDG----KTAFIG-S-LVVMLHHE----PILMGQISGL--------------DRDSLSAE----
-------VALDKL-RNF------------------D--GVVPELEEFLKESFR------------------------EHL
GVE---------------IT------------VP----EA--------------SSEEVVRA-------VVELSVRARHL
PDAGRLRL-IVGALLIPLVLCDYKGA------------EEREG------------ETPKFAQVL-----------EAEW
-------------------QGVV---

>2643433219.Ga0097913_10255

-----------------------MSEVLNFDEGETTSK-------------------NVLKGFKHEVLGSIYTFK-----
-VLRDLKL---------------DKEIPYIASWAVLLHHE----AMRR-KIKPEHLLTGIDDDV----------------
--------------------------------------IDQNAVILLRSLLKDNLHLSLNANT--------L--------
--------------------------------NT---D----------------KNEIGKIIDW-----LFK--YIYQN
QAKHILRM-KISSLQHIVCFCDVRAANRIRKGEV--T--------------S-PYFREVMRVFID---------P-----
----

>2533542706

-----------------------------M---IQEYAKPCAG----ERGTYTYHVSECYRLWEEIFQ---SRAVALACF
----CN-S--INI-SLKEFQEKSKKAVLFHDAGKLLPT--FQQQMQRLVEKQAPD---
PALHFRHELASALLLFA-----
-MEWKTLKN---NE-----------TLIPYEILAVLGHHK----PLQPDWQSFEREKICQE-------------SP----
------GLDEKAIQRIV--------------------QVAETYGLCCDL-------------------------DITATV
KR-FTFAKWPTLLLENGL-G----ERLQPT---------TL---GNI-------------------------------PA
SERRKLRL-AYALTKGLLMTCDWLASAKSEDQLPI---NHK------MTS---AVLRKK-LKEK----
VEQEGRKYEKRP
FHRACEEV-----------QGNILAIAPTGAGKTEAALLWATNG---------
EPSKIIFLMPTMVTSNSLYERMCKHYF
---E--EKECGLIHSGADTYF--------AQQDKFDQDNTV------------------NDFRLALYQFRAFMPPVMVAT
VDQMLTSGFNIGSWCQ------------KEW-ALVGSRVIFDEIHAYQSYTLGLITAAIEKIK-L--------
LGGTVCL
MSATMPAFLREHFQSLLEVSAPIV--A-----E--------ELMQRQKCHWKYRETPIN------------------EMA
GEIEDYLRQGK-KVALIFNTVRAAQEAYREWRKILG------
KERVLCYHSQFIMRDRQDKETALLMKDEK-----GRPN
NYDLVITTQVIEVSLDISFDVMYSECAPLDSLIQRAGRCNRYNADG-DYYFIVFPASEI-----------AVK-
YVYKG-
-AKAVLEKTAQI-VRQNQKRLSED------ELGQMLEY---VYDGYKLG---ADNTEYQEGYR------
LYDEIA-----
------------TSIDSKGFIFDLSISEE-TV---------------------TR-KFEDYL----KVNIIPQIFYG---
-----------EVEKLWA-----------------ERKYYLLRLYEVPISEYRR----------------RK-L--KSVD
NPMQLPIYEAPYTQEEGVLDSEDDFPTRLL--

>2732265458.Ga0128527_100911
MTHDERIPIDAVRIELYAPVASFRDPMFPGVSRCLPVPPPSTVRGMLAAATGRPTESVVLGMSAHAT
GRGTDVETYHLVW
ADGGDPPIGGRGGKRKGSTTVRERPFLTGVHVTLWIPLPDGARIAAALRRPVWGLRLGRSQDLVH
VRSVTNVTLHPAEDA
HIGHALAPVGGHDAPQGTLLRLADHVSSDRLRTGFADFLWCAEPPEHSRPVTGAYRD--
GDQAVWLLAPSSVDPADTLEP
EGDDEPPDVGREPHQSGQEQS--RLDEAEL---
TQVLGKSKGASKLGRPELLTEHSETVRDAARAVAG---------RI

----AS-P-GVLA-ARPGFWRQVETAALLHDAGKVAEG--FQRQLRP------GG---
EPWGERHEVLSLAYVDL-----
-LTRHLPPD---D-------------RKMITAGVAFHHL----PLTSSSRRDLRE--MYP------------PE----
------AAWKKKFGFDP-----------DAGPGRPRVQVPAARHTALLR------------------------WLAEQL
GV-------TA------LPSE-TRRLWELARDAF----ERV--QADWSS-----------------------------P
VP-PEDGL-VAVLLQGAVTLADRSGSAQVPLQEH------------MPLP--RDFIRT-L-------------VTPYP
HQQATADT-----------VGNLILCAPTGSGKTEAGLAWASRQLDDM----
PGRPRLVWVLPYRASIDAARDRFIRDLQ
APPGAAEPDIGVLHATAARTL--------LTRATADDCPPGPAEAR------------KARDQANA-
MRLFAQRVRVAT
PHQLLRAAIAGPSHSS------------LLL-EQANAVIALDELHAYDPATFGRLCAAMRLWR-D--------
LGSRVAV
LSATLAPPMIELIRDTLA-DVTVH--R-----A-P------PGTAPDRHRLVLDDQPID---------------APDSL
ARLRAWLAEGH-
SVLAVANTVATAQRVYAELAPAAHHACPDDPDAAILLHSRFRAKDRAEIERRILRRHPERSVGEPGH
R
GGGLVVATQVLEVSLCLDFDRGASELAPIEALAQRAGRVNRRGRHPDGPVEFRVHLPE-----------
TARPYKEDA-
-----LHAALSALRRTPEPVISEQ------TIDTWLRH---AYDTDWGR---EWTRQATRHRD------EFF--------
---------------DSFLTFTDPFHDRTEH--------------------AAGLTEAFD----TVEVLLERDAE---
-----------EYFDTLNGS---------------DGDPLLATGLLIPVSYAQL-----HRLAKDGRARLDRDH--ARRA
RHPPLWLIDAPYSTETGLDLSTANSSPV-PVETVL----------------------------------
----

>638173162.PF0639

-------------------------------MSCK--------AFQGQTLREHIEAMLAAWEI------VKNKYIPSI
-IRVMK-TVGVKF-TEEDADKFMKTLIILHDVGKCSEV--YQKHL-S------NN--
EPLRGFRHELVSAYYAYN-----
-----I--L--K--DMFKDE---TIAFIG-A-LVVMMHHE----PILMGQIRSL-------------DKEELTPE----
-------VVLDKL-RTF-----------------N--GVMEGTESFIKSMIK----------------------EKL
GVI---------------PK------------VP----SP-------------TQEDVLRE-------VIRLSVLARHR
PDSGKLRM-VVGALLIPLVLCDYKGA------------KEREG-------------ESPKFAEVL-----------RVEM
------------------MK---

>2523845491.H528DRAFT_01836

------------------------------MKYLA-RL-------PEELLSFHINKVAKLMKEMA------------
-----TSSQVP---I---DSEIIKLTGLFHDIGKYTEY--FQEHLLE------GK--SFGGKERHSFISALIAYE-----
-FLGNIYPN--------------AKQELFGAFTAIRHHHSDPQDFGVELNDTEPVWYQNVR----------HVK------
-AQ-VDSLLKASD-----EIRAIY----------PQ--EIVEAISS-----------------------LKSTHNIDEI

L-------------------------------------EKLRNVLF----------------SG----------------
--NISLYF-TTNFLLGMLVDADIRAVIGLKAN------EKRHE------------IPEDIVDRYINNLPK----NSPIYP
LRQEFYRTVINNIQRLGLENKFLSLTAPTGIGKTFAGFSAAVRLRSMINKETGRLPRIIYVLPFTSIIDQ
NFDEIAKVI-
---KHAG------
LPKNVLLKHHFRASPSESISGLKAEDAWKLLEEESLLRERDAQKLIKHYEQAHTRVETWDGEVVV
TT
FVRFYETLFTNRRSEM------------RRLHRLAGSIVILDEVQNIPVEYWEATEEALKFLAEK--------
WDARFIL
MTATRPAFVPEAYE------L----------------------TEPRKKHFFKILSRT--ALYVENNP-YD----YRDIE
SWLLPKIDGVQ-SFMVVMNTVRAAQDVYQMLKEAS-------DFDLCFLSASLIPKHREA-RIKDIRKS-
------LKNG
ARIGLVATQVVEAGVDLDFDLVVRDLAPFDSIVQAAGRCNRNARGENKGRVFLVKLINP----
EEHGRRLATYIYDSVL-
-----IGTTEELLQNQGIIPEKEYLRQVEEYFHKLRMEGRKAQDKNLLLSLKTMK--YDEVAM-------
FD-LL-----
---SQKIF----QVPVFVELDEEAEA
--------LIDKLKELEGLPTKTYDDRLKRRAMFKA-IAPEIWGYVINIPLKVA-----QGAGLEALPYAAS-
F--LWLR
R--ERLDFGDLYREETGFVRKIEHGAFFL---

>2587762900.EJ92DRAFT_02629

-----------------------------M---IQEYAKPCAG----ERGTYTYHVSECYRLWEEIFQ---SRAVALACF
----CN-S--INI-SLKEFQEKSKKAVLFHDAGKLLPT--FQQQMQRLVEKQAPD---
PALHFRHELASALLLFA-----
-MEWKTLKN---NE-----------TLIPYEILAVLGHHK----PLQPDWQSFEREKICQE-------------SP----
------GLDEKAIQRIV--------------------QVAETYGLCCDL-------------------------DITATV
KR-FTFAKWPTLLLENGL-G----ERLQPT---------TL---GNI-------------------------------PA
SERRKLRL-AYALTKGLLMTCDWLASAKSEDQLPI---NHK------MTS---AVLRKK-LKEK----
VEQEGRKYEKRP
FHRACEEV-----------QGNILAIAPTGAGKTEAALLWATNG---------
EPSKIIFLMPTMVTSNSLYERMCKHYF
---E--EKECGLIHSGADTYF--------AQQDKFDQDNTV------------------NDFRLALYQFRAFMPPVMVAT
VDQMLTSGFNIGSWCQ------------KEW-ALVGSRVIFDEIHAYQSYTLGLITAAIEKIK-L--------
LGGTVCL
MSATMPAFLREHFQSLLEVSAPIV--A-----E--------ELMQRQKCHWKYRETPIN------------------EMA
GEIEDYLRQGK-KVALIFNTVRAAQEAYREWRKILG------
KERVLCYHSQFIMRDRQDKETALLMKDEK-----GRPN
NYDLVITTQVIEVSLDISFDVMYSECAPLDSLIQRAGCNRYNADG-DYYFIVFPASEI------------AVK-
YVYKG-
-AKAVLEKTAQI-VRQNQKRLSED------ELGQMLEY---VYDGYKLG---ADNTEYQEGYR------
LYDEIA-----
------------TSIDSKGFIFDLSISEE-TV---------------------TR-KFEDYL----KVNIIPQIFYG---
-----------EVEKLWA-----------------ERKYYLLRLYEVPISEYRR----------------RK-L--KSVD
NPMQLPIYEAPYTQEEGVLDSEDDFPTRLL--

>2504153610.Desac_0091

------------------------------MFDLLNY--------SAPGEIYEDHVKFCLKAWAG------FKERMIPTL
-ARL------FDT-QIHEVKATVEMMIVCHDVGKLSAR--WQDYIKS------PK-
EKRSKGPPHATLGAPYVLY-----
-----F------NERSK------HDLYYA-GALAILMHHTDSG--LAQGNLEHP--------------AEDAINRR----
--------LVEYG-TER------------------------IRWAAGAEESFR------------------------RSV
GFSSLTEKLPEL---TEAITLGS---------LE----NMAKDLRQWARC---------------------------PRE
IDRHRRRL-QALALHHILKVCDWRAASQRPQQE-------------------------PDEDE-------------EGEK
IEKKCKQSL-----------LSVFLDGGLLP---

>638162626.SSO1403

----------------------------MT----CWA--------FYGMETFKQHSLGILNFFRDN-------FSYII--
PIISY--RTGI---DKETVRKSVEIGVSLHDIGKTSKY-------------------YDMSYFGHEFYSGYLVYK-----
-ILRECCD-----SE-----------LKPLIALAAMSHHQ----GMEGRTLNEMILKGNYT-------------------
---RI----------PSFYELREECR--NDIVEILGEI
GVK---------------VKD--------FPQKV----TR-SDVKSWFQKL-------NIKW------------------
-------KNLYVIILGPLMISDTVVA-----------NKNRGG--------D-QYNKI--IEEYE-----------KWIN
--------------------------VK--

>2775399572.Ga0226170_111640

---------------------------------MSLL--------AFQGQTLRKHVNAMLNAWES------VKGKYIPSI
-IRAMK-AWGVEL-SREDADRLMKALIILHDSGKGAEL--YQDYL-E------GK--
ARLSGFRHELVSAYYALK-----
-----I--L--P--QIFDE----KTAFVG-S-LVVMLHHE----PILMGQVANL--------------DRDSLSAE----
-------VALDRL-KNF------------------D--GVVPELNEFLENSFR------------------------EHL
GVE---------------IT------------VL----SA--------------SPDEVVRA-------VVELSVRARHL
PDAGRLRL-IVGALLIPLVLCDYKGA------------EEREG-------------ETPKFAEVL-----------EAEW
--------------------LGVV--

>2636760083.Ga0081690_11484

----------------------------MT----CWA--------FYGMETFKQHSLGILNFFRDN-------FSYII--
PIISY--RTGI---DKETVRKSVEIGVSLHDIGKTSKY-------------------YDMSYFGHEFYSGYLVYK-----
-ILRECCD-----SE-----------LKPLIALAAMSHHQ----GMEGRTLNEMILKGNYT------------------
---RI----------PSFYELREECR--NDIVEILGEI
GVK---------------VKD--------FPQKV----TR-SDVKSWFQKL-------NIKW------------------
-------KNLYVIILGPLMISDTVVA-----------NKNRGG--------D-QYNKI--IEEYE-----------KWIN
--------------------------VK--

>2682523895.Ga0128321_10830

------------------------------MNICYSY-N---KE-GGKVERYENHIKSAVAAWS-V-----IKPYYEKVL
-DRVL----------GKLEFDPIAVALAIHDLGKLTNA--YEMS---------------PRNFRHEIFSGYAMYK-----
-ILGKR-----------------PEDVKMILSLAVTLHHE----NILM-GVY----VGELGERY----------------
--------------------------------------LTISNIRRILDTFKDKLKPNCDVKR--------TFNELLKNF
LVR------------KGLIND----E-----FVN----DIMEFIDHWLNDGIK--IDDIIYSIKD-----MIAWASVGPL
EDLLIRRA-RVASVLHLITLADSIAANVLRS------GDMKDE--------G-NWIVERVSKGAE---------
PVDINE
IKDAINKVM-------------GNAP--

>2717094174.Ga0170752_1046

----------------------------------MIY--------AAPDETLEDHVRVALEFLYGDRL---SLAKCSKAL
-ARASKINLGIGV-NEEEARDVINLAIALHDIGKAYAE--YQQMILK------
AKSSKDFSVPRHEVYSAFAINK-----
-----L--L--D--KRVNEL---WECM---I-LSVLWHHY----PTRGENIAVI--------------FRTMREY-----
-------LRIGKV-TIS------------------R--EAVEEVRSIVEASLE-------------------------GV
GVR---------------VDRYNLQ-WNAFPREV----EL--------------GDRELFDFLN--------GLNDKFAL
PHPERIYA-TALSILYPLQLCDIYSA-----------SKNRGL--------K-SWGEFPMYLEEL-----------PSYM
--------------------FRENLRVLLA--

>646612990.Ferp_1863

------------------------MNEQILVWTDEKD--------LSKRQTYEEHVNGMIECWNK--Y---LKKKYAKSL
-TRVLR-VN------SSLLDEIVKFTLILHDAGKCTLY--YQQNIT------------SPKRYRHEIVSAYLAYR-----
-----L--S--E--DVFEKR---IRHVISG---SIFIHHE----PILMGSISFQ--------------RERGVTLTDIRG
RIEYPSSSRLEKV-EST------------------K--IMHNNFKSLLESLLI------------------------NHL
NSN---------------TIDKMLDNLRSI--KT----EL--------------SVDDIVNTIGN----IVSTLALAGDD
TWRHMARI-QVSALQYILVVCDYYGS------------KNRDS-------------EESKFQEEI-----------KWEW
--------------------GEET--

>2940372979.Ga0505229_12_24308_26617

---------------------------------MTLLAKSKDSKSNISAQTLTEHINACLLVWERIKF----------AF
----PE-VATVSG-IGGRFWDILRQCIIFHDLGKAHIE--FQKLLHE-K----PN---
QWKNQRHELFSIPFVDA-----
-LTSED-QE---I--------------LSLIRFVILGHHK----DYETLRNAISFYEI----------------------
----------ESFGGLS-----------GL-------DETEN----FSA-------------------------AFENHV
DI-------DG------VKQ--VLNEFEITIETV----MPKS-VSKWIHQYIS------------------------RPC

RLDNDKSL-LLFLLFGGLKWCDHLGSAMVSNIPVL------------HMSD--FSFLDS-KRA------
RLCQQGYDFYA
HQLVCSQT-----------VGNVVLTAPTGSGKTEAALLWARKQMEQS----
GQTGRVYYILPFTASINAMYERLSEYF-
---GKEKEMVGMLHGKLSDYL--------NNYFEDAQYDLGTKKEE---------------
IQQIRSKFRSLTTSVKVVT
PFQLLKHIFGLKGYEQ------------GFF-EMAGSYLIFDEIHAYSPDVFAQIKVLLEFAQKY--------
LNSHVLI
MTATMPKFLQAELEQSIKGPKLVQ--A-----DID------LYKKFVRHRVVLQEGLLA------------------DST
SNIIDCLQRDM-KVLVVCNTVLSAQNVFRDLCKYVG------KDEAVLLHSSFTGKDRSLKEQMLKL--
----------N
DIRLLVGTQAIEVSLDIDYDIIFTEPAPIDALIQRFGRVNRKREKEICDCIVFREANNS-----------
DKFIYPTEI-
-VYK-TIQALEQIIHEKGGVVDEA------LLQNSIDF---VYD-QWGE---DGKTKFDK-QY------FYL--------
----------------NEAIKLLAPMQKNKVQ---------------------EEDFYKQFD----GVKILPQSLVG---
-----------EYTTCMNQF-----------------DYISAESYKVSISSRRFKYWIMNDFVRLGSVHTSKKD----KI
IENRYYMTNKTYSSELGLLMDEEDY---WVESDFL---

>2264868846.A471B5DRAFT_00560

--------MTLNSIY--LELCNERAW------------------------------KPRYFIEETLN-------------
---KLENL------LKENKRFVLIARLPTGYGKTTITNVLARAALKN----
NPYFCRVIHVLPMRSIADDVCLELQK---
--------DKM---ISNHVAVQHLLT--PGSPYFAKKCIVTT
LDTFLLNFFKIPTPELKKVFQYDTAHSEFPRAMIYLSIVIFDEFHLFAGLG-
DISNEGKALTSVIASMICLLKAKVPIII
MGAT----IPDVLIDKIREEISIVG----------------GEVESVNYVFDKDLEFDEK-----LRKKKKRIHIEEKDV
MEILNNIDSSK-SVLIVVNTVKKAKEIYNSITNKEEVG---------FLHGKIPEA-----IRAEIIKK-------
IKNS
KPRILIATQVIEAGVNISYDILITEPCPIDRLIQRAGRVCRFDEEEGD-----IYISKF----------
EKDYIYDENF-
-----VKTTLEVLNPNKGIERKES------NVLTF------EFANQAINEVYKI------------PIKELKEIDYDMKG
FLQELDALPILTSKYAK-------------------------------ELIIRYKGFTENFG----IVSCFNEKYLNKLY
AIPVSEKEGWELIKKHGKL-----IDANYRLIDFKAKLR-EVESLSIYL----------LDKGYQGIA------------
------
IPEEEYIEIIGFRPKIDIKTSASASKNISSSPSHHEISKPCAFIGQSLIKHTENTLEKALKMYSFETMSK
RFE
ALGININAERLEKLIKAACILHDLGKAADEYQEDFYNSCECKG--
KVSFYLHEVASAYYAYKKLKSIEDYELDTKERQLI
TLAILFHIAGKNLYDLKDGVKEKKMKWTFNKYWKSFEHLLNKHRIKVDYKSSSINTEEAMNFLEE
IEEIIRKRESYYLKL
YNLIYSAICVGDNLDSYEFRRTDISESRKIFIEELKEVIANA
>2839666852.Ga0335788_2663

--------------------------VNPL---NRIWAKSASP-GQSWGELLTEHLDATLTALDLLRH----------RV
----GR-I-A--A-VPDRFWTWAALACLFHDAGKIPEG--FQRMVGNPR----PA---

QVWGLRHEIYSLGFVDH-----
-VLAHLDED---E--------------RQWIALGVLTHHR----PLSGGARSIRKQKGSLR-------------TP----
------QAVTDAFG-----------------------PVDEQTANALTA-------------------------WLAQRC
YA-------PV------PKPVTATDLGKATHRLL----TTV--LDHWAE------------------------------E
SPDNEAGL-HAVLLQGAVTLADHVASAHTTLLTD-------------HPLD--AEYPER-LRK------
RLTNQGATLFP
HQEAAARA-----------
SGHLLLRAPTGKGKTEASLLWALTQIDQVRATTGGQPRLFYTLPYLASINAMADRLGEELD
DPE---RQSIGVTHSKAADYH--------LRRAINDDHDETEPLEH-----------ATRAVAKANA-
SRLFRELVRVTT
PYQLMRAALAGPAHSS------------TLI-DSVNSVFVFDELHAYDTHRLGIILAMTGMWA-R--------
LGGRIGV
VSATLPDALAELIEKTLGEPLAEV--A-----PDS------GQAWPRRHRLHLDETHLT----------------STESI
AAITEQLVQGN-SVLVVANNVADAQNIYDTLAPTARELYGD--
DGAILLHARFRAKDRGEIERRILERYGT-----NQKH
HPGLVVATQVVEVSLDLDFDILHTSAAPLEALIQRFGRVNRLNGRDQ-
SAPVVVHRPDYAPRARGGGDEYADKVYAAEP-
-----TRLGWDILIRHDGDPLDER------LFTDWLNE---VYTSPWGQ---RWRSDVERVRT------EFT--------
----------------RRFLTFDPPFDDRSDL---------------------AAAFDQMFD----GAEGILAEDLE---
-----------AYREALDQGR----DPQT-----RKAARLLASGYLISLPDH------------------ARRL--GRWD
KEFGLIVIDADYTEEKGLGALNRDDRTSYVMGEVL--

>2559588888.OMM_04547

----------------------------------MII-SHPATH-QAPAKTLKDHLTTVADKSRRQIM------------
-----RMKLNLSLITSVELADLSYLIGLFHDFGKLSTF--FQNYINQ------QG--
SRSALTHHSLISAFVCFH-----
-VLESLYPE--------------DVWPMIG-YLIIKRHHGNLETLDTETIPAVK-----------------NIF------
-VQ-LNDILDNAS----DEIQHIY----------M---GTIPNISEILSTISF-----------------DRYADIIDDI
PDR-----------------------------L----------------EDLLDEF-NSCA-------------------
--AIELFF-IVNLLFSVLIDSDKKDAARLDNTYFKENLEETHN---------------DVFAFFKALSDRK---------
----

>2730044867.Ga0180935_10143

-----------------------MSEAYA----YLY--------GDRKQTLREHIYSCLEALDKIKS---SKI------
----W--NLVKKL-CGSSAERYIKIAVIFHDSGKILYQKNFKTKN----------DVKYLSFYGHEIYSTYFISE--
---
-FLNAKRR-----AIENVKQLDVLRLFKLAVIASVLYHHH----AMGVRXRIKRLSKISYE-------------------
----------------------------------N--VFVKEFDKLIEDLLENPIFLSDILKNKSE------------

---------------------GHLSP-NNVSNVV----SD-LNREIWIEFVKS-K-----KF------------------
-------RKTMLLFTNILLIADYMGS-----------QDRDGT--------K-TEFAK-VLDEFL-----------ELYS
--------------------------TSTHPAP---

>2688750946.Ga0133540_111254

----------------------------MS---RQYWAKP--------DQTYEEHITAAYRAWRETVA---GKQALIRRT
----GR-T--CGF-EPDRFLQSSLLTVVLHDIGKNILP--FQRMMDTIRAGKRAD---
PRENYRHEIVSYAFTLH-----
-AGFALQQQ---EGSL-------LPVPLPIEALAVLGHHK----PVNPNMEGFHRELTAD--------------AP----
------IVCDDGVARA-------------------------------I------------------------DLARDI
FA-EEGYTLPRFRLPK---G----NPMDPVKRLI----GPG---GGFPKFY-------------------------EQF
NDPDLLRS-PYALLKGILHYADWNGSAGTPICYSL---QAR------ESD-----LLTH-IQARC---
KAKKIQFTGLRP
FQEACAST-----------LGHCIAIAPTGSGKTEGSLLWALRNLEEM-----
GDGKVIYLLPTMVTANSIFHRLE-DYF
---G--KGNVGLTHSTASLLF--------AEEEEDR----------------------TAVRHA-LFDKTFMTPATVAT
VDQLLFAGFNTGKWPV-----------VEA-NAANSAIVVDEIHAYDPWTLGLLLEAIRHYS-R--------
LGARFML
MSATLPAYVLDLLTQALPDATIVR--D-----E--------TLLASARNRFQTVDAPLE-----------------EAV
DAIIEAVEGGA-KTLVVANSVGACQDLYTKFKKV--------GLDPLCYHSKFTLQDRAEKEQKINSDS---
-------E
SPGLLIATQVVEVSLDIDFDLMFSECAPPDAIVQRAGRVNRRREKD-DTCITLYRPSDV----------
SEKIYDPD--
-RTGLLDRTFEA-FRDAPFRPTEA------DLTKIVEL---VYSGMDIA---KDSR-FREASA------QYA--------
-----------LSQGQRMAIFDNPQRDPHYE--------------------VT-RKVDYP----QVSVIPLTFRD---
-----------AVTSLSPL----------------ERRWYE-----VKMPAWYV---------------KK-R--REVR
G--DVVFCEMEYDAGLGARFTDTPELAYMMT---

